# Associations among circulating sphingolipids, β-cell function, and risk of developing type 2 diabetes: A population-based cohort study in China

**DOI:** 10.1371/journal.pmed.1003451

**Published:** 2020-12-09

**Authors:** Huan Yun, Liang Sun, Qingqing Wu, Geng Zong, Qibin Qi, Huaixing Li, He Zheng, Rong Zeng, Liming Liang, Xu Lin

**Affiliations:** 1 CAS Key Laboratory of Nutrition, Metabolism and Food Safety, Shanghai Institute of Nutrition and Health, University of Chinese Academy of Sciences, Chinese Academy of Sciences, Shanghai, China; 2 CAS Key Laboratory of Systems Biology, Shanghai Institute of Biochemistry and Cell Biology, Center for Excellence in Molecular Cell Science, Chinese Academy of Sciences, Shanghai, China; 3 Department of Epidemiology and Population Health, Albert Einstein College of Medicine, Bronx, New York, United States of America; 4 Department of Epidemiology, Harvard T.H. Chan School of Public Health, Harvard University, Boston, Massachusetts, United States of America; 5 Department of Biostatistics, Harvard T.H. Chan School of Public Health, Harvard University, Boston, Massachusetts, United States of America; 6 Key Laboratory of Systems Biology, Hangzhou Institute for Advanced Study, University of Chinese Academy of Sciences, Chinese Academy of Sciences, Hangzhou, China; 7 Shanghai Institute of Nutrition and Health, Chinese Academy of Sciences, Shanghai, China; University of Texas Health Science Center at Houston, UNITED STATES

## Abstract

**Background:**

Animal studies suggest vital roles of sphingolipids, especially ceramides, in the pathogenesis of type 2 diabetes (T2D) via pathways involved in insulin resistance, β-cell dysfunction, and inflammation, but human studies are limited. We aimed to evaluate the associations of circulating sphingolipids with incident T2D and to explore underlying mechanisms.

**Methods and findings:**

The current study included 826 men and 1,148 women who were aged 50–70 years, from Beijing and Shanghai, and without T2D in 2005 and who were resurveyed in 2011. Cardiometabolic traits were measured at baseline and follow-up surveys. A total of 76 sphingolipids were quantified using high-coverage targeted lipidomics. Summary data for 2-sample Mendelian randomization were obtained from genome-wide association studies of circulating sphingolipids and the China Health and Nutrition Survey (*n* = 5,731). During the 6-year period, 529 participants developed T2D. Eleven novel and 3 reported sphingolipids, namely ceramides (d18:1/18:1, d18:1/20:0, d18:1/20:1, d18:1/22:1), saturated sphingomyelins (C34:0, C36:0, C38:0, C40:0), unsaturated sphingomyelins (C34:1, C36:1, C42:3), hydroxyl-sphingomyelins (C34:1, C38:3), and a hexosylceramide (d18:1/20:1), were positively associated with incident T2D (relative risks [RRs]: 1.14–1.21; all *P* < 0.001), after multivariate adjustment including lifestyle characteristics and BMI. Network analysis further identified 5 modules, and 2 modules containing saturated sphingomyelins showed the strongest associations with increased T2D risk (RR_Q4 versus Q1_ = 1.59 and 1.43; both *P*_trend_ < 0.001). Mediation analysis suggested that the detrimental associations of 13 sphingolipids with T2D were largely mediated through β-cell dysfunction, as indicated by HOMA-B (mediation proportion: 11.19%–42.42%; all *P* < 0.001). Moreover, Mendelian randomization evidenced a positive association between a genetically instrumented ceramide (d18:1/20:1) and T2D (odds ratio: 1.15 [95% CI 1.05–1.26]; *P* = 0.002). Main limitations in the current study included potential undiagnosed cases and lack of an independent population for replication.

**Conclusions:**

In this study, we observed that a panel of novel sphingolipids with unique structures were positively associated with incident T2D, largely mediated through β-cell dysfunction, in Chinese individuals.

## Author summary

**Why was this study done?:**

- Ceramides are members of the sphingolipid family that have been reported to be pathophysiologically relevant for the development of impaired glucose homeostasis and type 2 diabetes (T2D) through inducing insulin resistance, impairing β-cell function, and promoting inflammation.
- Sphingolipids represent a class of structurally and functionally diverse lipids that may exert different impacts on T2D outcome. However, the majority of available studies covered only 4 to 15 species and may have missed certain T2D-associated sphingolipid species or subclasses.
- To our knowledge, no epidemiological study has examined the relationships between genetically predicted sphingolipids and T2D risk so far.
- Most previous studies of sphingolipid–T2D associations were conducted in Western populations. It remains largely unknown whether the associations differ between Western and Asian populations, and to what extent they are mediated by β-cell dysfunction or insulin resistance.

**What did the researchers do and find?:**

- By applying high-coverage targeted lipidomics to measure 76 sphingolipids in 1,974 Chinese men and women, 11 novel (3 monounsaturated ceramides, 7 sphingomyelins, and 1 hexosylceramide) and 3 previously reported sphingolipids were identified to be positively associated with incident T2D, independent of conventional risk factors including lifestyle characteristics and BMI.
- We conducted a network analysis to determine the collective effects of the sphingolipids on the onset of T2D. Of 5 identified groupings (modules) of sphingolipids, 2 modules containing saturated sphingomyelins showed the strongest associations with incident T2D.
- Mediation analysis showed that the detrimental associations of 13 out of the 14 sphingolipids with risk of T2D were largely mediated through β-cell dysfunction.
- We performed genome-wide association studies for the sphingolipids and also conducted 2-sample Mendelian randomization analysis. A positive association between a genetically instrumented ceramide (d18:1/20:1) and T2D was evidenced.

**What do these findings mean?:**

- Our study is the first to our knowledge that simultaneously discovered the largest number of T2D-associated sphingolipid species—which covered 3 subclasses, including ceramides, sphingomyelins, and a hexosylceramide—and also identified predominantly monounsaturated ceramides associated with T2D in the Chinese population, rather than saturated ceramides as reported in Western populations.
- High-coverage targeted lipidomics in our study revealed comprehensive details about how and to what extent altered absolute concentrations of sphingolipids with specific structures were associated with T2D development, raising attention to structurally diverse sphingolipids and the fact that they might have different associations with T2D.
- Our results highlight a stronger mediation effect of β-cell dysfunction than of insulin resistance in the sphingolipid–T2D associations in Chinese individuals.
- We found a positive association between a genetically instrumented ceramide (d18:1/20:1) and T2D. Together, our findings underscore potential applications of sphingolipids as early biomarkers or intervention targets for prevention and control of T2D.

## Introduction

The recent epidemic of type 2 diabetes (T2D) and related complications have contributed enormously to global burdens of mortality and disability [1]. Though diabetes affects approximately 8.3% of the world population, a large fraction of patients is undiagnosed, accounting for 21.4% out of 34.1 million and 63.5% out of 114.4 million American and Chinese adult diabetes cases, respectively [2]. Due to its heterogeneous nature, it is still not fully clear whether β-cell dysfunction or insulin resistance plays a primary role in the pathogenesis of T2D, particularly in Asian populations with relatively lower β-cell function [3]. In this regard, it is key to restrain the progress of T2D long before clinical diagnosis through early prediction and intervention.

Sphingolipids represent a class of structurally and functionally diverse lipid molecules (S1 Fig), including ceramides (Cers), sphingomyelins (SMs), and glycosphingolipids (GSLs) [4]. Cers, commonly combining a sphingoid base with a fatty acid residue, can be de novo synthesized from dietary fatty acids and serine in the endoplasmic reticulum. Subsequently, Cers can form SMs by adding a polar head group or form GSLs by introducing a sugar group in the Golgi complex [5]. Recently, the pivotal roles of sphingolipids in a variety of metabolic disorders have attracted growing attention [6–8]. Studies in animal models indicated that elevated Cer levels dysregulated glucose homeostasis and accelerated T2D progression by antagonizing insulin-receptor-stimulated serine/threonine–protein kinase signaling [5,9], while high SM levels in vivo induced insulin resistance via enhancing mitochondrial dysfunction, reactive oxygen species production, and inflammation [9]. Moreover, GSLs in rodents could induce metabolic toxicity [9].

With recently advanced lipidomic technology, over 600 sphingolipids were detected in humans [4]. However, only a handful of human studies have investigated the effects of sphingolipids on fasting glucose [10], insulin resistance, and β-cell dysfunction [11]; even fewer prospective cohorts have studied T2D per se, and these yielded controversial results [12–15]. For instance, the association between SM(d18:1/18:0) and incident T2D was inverse in the PREDIMED Trial [12], but positive in a Singapore cohort [13].Notably, Cers and SMs with distinct chain lengths, numbers of double bonds, and numbers of hydroxyls on the sphingoid base and/or fatty acid residue may influence metabolic outcomes differently, and distinctive structures may reflect genetic and dietary modifications in a given population [9,16,17]. However, the majority of available studies generally covered 4 to 15 species and focused on sphingolipids combining an unsaturated sphingoid base (e.g., d18:1) with a saturated fatty acid (SFA), not those combining a saturated sphingoid base with a SFA or monounsaturated fatty acid (MUFA) [12–15]. Thus, it is important to elucidate how sphingolipids with different structures affect T2D susceptibility.

By adopting a high-coverage targeted lipidomic approach in a well-characterized Chinese cohort, we aimed to investigate (1) the associations of different sphingolipids with incident T2D, (2) specific sphingolipid networks linked to incident T2D, and (3) possible mediators and relationships of genetically instrumented sphingolipids with T2D.

## Methods

### Study population

The current study was based on the Nutrition and Health of Aging Population in China (NHAPC) study, a well-designed prospective cohort investigating environmental and genetic factors and their interactions with cardiometabolic diseases. The study design has been described in detail elsewhere [18,19]. Briefly, this cohort study was initiated in 2005, and a total of 3,289 participants (1,458 men and 1,831 women) aged 50–70 years from Beijing and Shanghai were enrolled by multistage sampling at baseline. In 2011, 2,529 participants were revisited to complete the follow-up survey. At the baseline and 6-year surveys, a face-to-face interview was conducted to collect information on demographics, lifestyle characteristics, and health status using a standardized questionnaire with minor modification for the 6-year survey. Family history of diabetes was defined as a parent or sibling having diabetes [20]. Body weight, height, waist circumference, and blood pressure were measured following a standardized protocol [18]. Body mass index (BMI) was calculated as weight (kg)/height squared (m^2^).

Final analyses included 1,974 individuals (826 men and 1,148 women) after excluding those with diabetes at baseline (*n* = 274) or without baseline sphingolipid profile (*n* = 281). The analysis plan was drafted in August 2018 (S1 Text), and our cohort study was reported according to the Strengthening the Reporting of Observational Studies in Epidemiology (STROBE) guideline (S1 STROBE Checklist).

### Ethics statement

The study protocol was approved by the Institutional Review Board of the Institute for Nutritional Sciences. All participants provided written informed consent.

### Laboratory measurements

Peripheral venous blood samples were collected after overnight fasting at both the baseline and follow-up visits. Fasting glucose, glycohemoglobin (HbA_1c_), insulin, total cholesterol, low-density lipoprotein cholesterol (LDL-C), high-density lipoprotein cholesterol (HDL-C), triglycerides (TGs), high-sensitivity C-reactive protein (hsCRP), and adiponectin were measured as previously described [18,19]. Homeostatic model assessment of insulin resistance (HOMA-IR) was calculated as fasting glucose (mmol/l) × fasting insulin (μU/ml)/22.5. Homeostatic model assessment of β-cell function (HOMA-B) was calculated as 20 × fasting insulin (μU/ml)/(fasting glucose [mmol/l] − 3.5).

### Genotyping, quality control, and imputation

Genotyping was performed using Illumina Human660W [21]. Samples with call rate < 97% or single nucleotide polymorphisms (SNPs) with call rate < 95% were excluded. Imputation was conducted from the 1000 Genomes phase 3 reference panels using IMPUTE2. SNPs were further removed by PLINK if minor allele frequency < 1%, Hardy–Weinberg equilibrium *P* < 10^−6^, or info measure ≤ 0.5. Finally, 7,118,257 SNPs were obtained for the following genetic analyses.

### Lipidomic measurement

Plasma lipid profiles were quantified by liquid chromatography electrospray ionization mass spectrometry [22,23]. Details on lipid extraction, chromatographic separation, mass spectrometry analysis, and data quantification are provided in S2 Text. Briefly, lipids were extracted following a modified methyl tert-butyl ether (MTBE) protocol and then analyzed on a Shimadzu Nexera X2 LC-30AD system coupled to a SCIEX 5500 QTRAP mass spectrometer [4]. Analyst 1.6.3 software (Sciex, Foster City, CA) was used for data acquisition. Plasma samples were analyzed in random order, and quality control samples were inserted every 10 samples to ensure repeatability. Finally, a total of 728 lipids were quantified.

Current analyses used data of 76 sphingolipid species, including 12 Cers, 9 dihydroceramides (dhCers), 43 SMs, and 12 GSLs. dCer(d16:0) was used as the internal standard for Cers, dhCers, and GSLs, while dSM(24:1) was used for SMs. As shown in S1 Table, the average coefficient of variation was 18.3%. Eight out of 76 sphingolipids had missing values (<1%). The SM subclass was further classified as (1) saturated SMs, with no double bond in the sphingoid base and fatty acid; (2) unsaturated SMs, with 1 or more double bonds in the sphingoid base or fatty acid; (3) hydroxyl-SMs having 1 additional hydroxyl; and (4) hydroxyl-SMs having 2 additional hydroxyls (S1 Fig).

### Ascertainment of incident T2D

Incident T2D was defined as presenting 1 or more of the following: (1) self-reported doctor-diagnosed diabetes, (2) taking antidiabetic medications, or (3) fasting glucose ≥ 7.0 mmol/l in the follow-up survey.

### Statistical analyses

Baseline characteristics were compared between participants with and without incident T2D by ANCOVA. Missing values for 8 sphingolipids were imputed as half of the minimum value, due to their concentrations being below the detection limit [24]. Baseline sphingolipid concentrations (mg/l) were natural log transformed and scaled to SD of 1. Spearman correlation coefficients (*r*_s_) were calculated among sphingolipids, as well as their associated cardiometabolic traits, adjusting for age, sex, region (Beijing or Shanghai), and residence (urban or rural). Due to high T2D incidence in our cohort population, a log-Poisson model was used to calculate the relative risk (RR) and 95% confidence interval (CI) for T2D according to quartile and per SD increment of each sphingolipid, after adjustment for age, sex, region, residence, educational attainment (0–6 years, 7–9 years, or ≥10 years), current smoking (yes or no), current alcohol drinking (yes or no), physical activity (low, moderate, or high), family history of diabetes (yes or no), and BMI in the full model [25]. Significance levels were corrected for multiple testing with Bonferroni correction. In a sensitivity analysis, incident T2D was redefined by adding HbA_1c_ ≥ 6.5%, and the log-Poisson model was used to estimate the RR (95% CI) of T2D associated with the sphingolipids. Stratified analyses were performed for different baseline values of age, sex, region, residence, smoking, alcohol drinking, physical activity, BMI, and HOMA-B. *P*_interaction_ was calculated by likelihood ratio test.

#### Network inference analysis

Weighted gene co-expression network analysis (WGCNA) was used to determine modules of highly interconnected sphingolipids (R package WGCNA, version 1.63). Neighborhoods of interconnected sphingolipids were defined by topological overlap measure (TOM). The modules were represented by the first principal component of the metabolites included in the modules. Finally, network plotting was performed using Cytoscape (version 3.7.1).

#### Multiple mediation analysis

Mediation models were performed using the SPSS macro for simple and multiple mediation analysis by Preacher and Hayes (see [26]). Different paths were produced in the model: Path a represents effects of sphingolipids and/or module eigengenes on mediators, path b represents effects of mediators on T2D, path c represents effects of sphingolipids on T2D not through mediators, and path a*b represents effects of sphingolipids on T2D through mediators. The bootstrapping method was applied, with coefficients estimated from 1,000 bootstrap samples.

#### Mendelian randomization (MR)

Before MR analysis, genome-wide association studies (GWASs) for individual sphingolipids were conducted (S2 Text). The relationships of genetically predicted sphingolipids with T2D were analyzed using 2-sample MR analysis (R package MendelianRandomization, version 0.3.0). Summary statistics of the relationships of SNPs with T2D were from the China Health and Nutrition Survey (http://mohlke.web.unc.edu/data/) [27]. The overall instrumental estimate of the effect of any given exposure on outcome was calculated using the penalized robust inverse variance weighting (IVW) method. In sensitivity analyses, the weighted median test, mode-based estimation, MR-Egger regression, and leave-one-out validation were also conducted. More details are provided in S2 Text.

All analyses were performed with SPSS version 25.0 (IBM, Armonk, New York) and R version 3.4.4 (http://www.R-project.org). Two-sided *P* value < 0.05 was considered statistically significant unless specified otherwise.

## Results

### Baseline characteristics

Over 6 years, 26.8% (529/1,974) of participants developed T2D. Compared with non-cases, cases were more likely to be Beijing residents and to have a family history of T2D. They also had higher baseline values of BMI, waist circumference, blood pressure, fasting glucose, HbA_1c_, insulin, and HOMA-IR, but lower HOMA-B (all *P* < 0.001), as well as unfavorable profiles of LDL-C, HDL-C, TGs, hsCRP, and adiponectin (all *P* < 0.05; Table 1). Among the 3 sphingolipid subclasses, SMs had the highest concentrations. Within the Cer subclass, Cer(d18:1/24:1) had the highest mean level and Cer(d18:1/26:1) had the lowest mean level (3.03 [95% CI 2.90, 3.15] versus 0.041 [95% CI 0.038, 0.045]; *P* < 0.001). Levels of saturated and unsaturated SMs were much higher than levels of hydroxyl-SMs. Compared with non-cases, cases had significantly higher concentrations of 34 sphingolipids, including 6 Cers, 5 dhCers, 20 SMs, and 3 GSLs after Bonferroni correction (all *P* < 0.001; S2 Table).

**Table 1:** Baseline characteristics of study participants with and without incident T2D.

Characteristic	Incident T2D		*P* value
	Non-cases (*n* = 1,445)	Cases (*n* = 529)	
Age (years)	58.0 (6.0)	58.3 (6.0)	0.13
Male	593 (41.0%)	230 (43.5%)	0.72
Beijing residents	617 (42.7%)	317 (59.9%)	<0.001
Urban residents	596 (41.3%)	222 (42.0%)	0.63
Educational attainment ≥ 10 years	263 (18.2%)	109 (20.6%)	0.92
Current smoking	400 (27.7%)	146 (27.6%)	0.15
Current alcohol drinking	343 (23.7%)	138 (26.1%)	0.82
High physical activity level	787 (54.5%)	290 (54.8%)	0.67
Family history of diabetes	138 (9.6%)	67 (12.7%)	0.05
BMI (kg/m^2^)	23.9 (3.3)	25.5 (3.7)	<0.001
Waist circumference (cm)	81.7 (9.9)	86.5 (11.0)	<0.001
Systolic blood pressure (mm Hg)	136.5 (21.3)	144.6 (22.6)	<0.001
Diastolic blood pressure (mm Hg)	78.9 (10.5)	82.0 (10.6)	<0.001
Fasting glucose (mmol/l)	5.2 (0.3)	5.7 (0.6)	<0.001
HbA_1c_ (%)	5.7 (0.4)	5.9 (0.5)	<0.001
Fasting insulin (pmol/l)	91.2 (66.2, 125.4)	99.6 (73.1, 135.1)	<0.001
HOMA-IR^a^	3.0 (2.2, 4.2)	3.5 (2.6, 4.8)	<0.001
HOMA-B^a^	155.0 (109.4, 218.4)	140.3 (101.0, 181.6)	<0.001
Total cholesterol (mmol/l)	4.6 (0.9)	4.8 (1.0)	0.06
LDL-C (mmol/l)	3.2 (0.9)	3.3 (1.0)	0.04
HDL-C (mmol/l)	1.3 (0.3)	1.3 (0.3)	<0.001
Triglycerides (mmol/l)	1.0 (0.7, 1.5)	1.1 (0.8, 1.8)	<0.001
hsCRP (mg/l)	0.6 (0.3, 1.2)	0.8 (0.4, 1.7)	0.01
Adiponectin^b^ (μg/ml)	14.9 (9.1, 23.3)	12.5 (8.0, 20.6)	<0.001

Data are mean (SD), median (interquartile range), or *n* (%). Percentages may not sum to 100% because of rounding. *P* values were calculated adjusted for age, sex, region (Beijing or Shanghai), and residence (urban or rural).

^a^Data are missing for 2 participants.

^b^Data are missing for 53 participants.

HbA_1c_, glycohemoglobin; HDL-C, high-density lipoprotein cholesterol; HOMA-IR, homeostatic model assessment of insulin resistance; HOMA-B, homeostatic model assessment of β-cell function; hsCRP, high-sensitivity C-reactive protein; LDL-C, low-density lipoprotein cholesterol; T2D, type 2 diabetes.

### Sphingolipids and cardiometabolic traits

Sphingolipids were highly intercorrelated (*r*_s_ > 0.5) within the same subclasses. As precursors of Cers, dhCers showed moderate to strong correlations (*r*_s_ = 0.27 to 0.90; all *P* < 0.001) with Cers and GSLs carrying the same fatty acid (S2 Fig). Regarding the cardiometabolic traits, most Cers, dhCers, and SMs with long-chain fatty acids were positively correlated with fasting glucose (*r*_s_ = 0.11 to 0.54), HbA_1c_ (*r*_s_ = 0.09 to 0.22), insulin (*r*_s_ = 0.10 to 0.16), HOMA-IR (*r*_s_ = 0.09 to 0.22), total cholesterol (*r*_s_ = 0.11 to 0.49), LDL-C (*r*_s_ = 0.11 to 0.50), and TGs (*r*_s_ = 0.10 to 0.46), but inversely correlated with HOMA-B (*r*_s_ = −0.26 to −0.09) (all *P* < 0.001; S3 Fig).

### Sphingolipids and incident T2D

As demonstrated in Tables 2 and S3, 14 sphingolipids (Cers: d18:1/18:1, d18:1/20:0, d18:1/20:1, d18:1/22:1; saturated SMs: C34:0, C36:0, C38:0, C40:0; unsaturated SMs: C34:1, C36:1, C42:3; hydroxyl-SMs: C34:1, C38:3; and a hexosylceramide [HexCer]: d18:1/20:1) were positively associated with incident T2D after multivariate adjustment including lifestyle characteristics and BMI (RR_per SD_: 1.14–1.21; all *P* < 0.001). Of note, 3 out of 4 of the abovementioned Cers were monounsaturated Cers (with MUFA in the acyl chain). The sphingolipid–T2D associations did not change when BMI was replaced with waist circumference, but slightly attenuated when baseline hsCRP and adiponectin were also adjusted for in the model. Further controlling for fasting glucose abolished the significant associations (S4 Table).

**Table 2:** Associations (RRs [95% CIs]) between baseline sphingolipids and incident T2D.

Sphingolipid	No. of carbons	No. of dbs	No. of -OHs	Quartiles of sphingolipids				*P*_trend_	RR per SD	*P* value
				Q1	Q2	Q3	Q4			
Cer(d18:1/18:1)	36	2	2	1	1.09 (0.87, 1.37)	1.20 (0.97, 1.49)	1.40 (1.14, 1.72)	<0.001	1.14 (1.06, 1.22)	<0.001
Cer(d18:1/20:0)	38	1	2	1	1.18 (0.94, 1.47)	1.24 (1.00, 1.54)	1.50 (1.22, 1.84)	<0.001	1.14 (1.06, 1.22)	<0.001
Cer(d18:1/20:1)	38	2	2	1	1.08 (0.86, 1.35)	1.18 (0.95, 1.47)	1.42 (1.16, 1.75)	<0.001	1.18 (1.10, 1.26)	<0.001
Cer(d18:1/22:1)	40	2	2	1	1.16 (0.90, 1.49)	1.35 (1.05, 1.73)	1.60 (1.25, 2.05)	<0.001	1.17 (1.08, 1.27)	<0.001
SM C34:0	34	0	2	1	0.97 (0.76, 1.24)	1.12 (0.88, 1.43)	1.38 (1.08, 1.76)	<0.001	1.16 (1.06, 1.26)	<0.001
SM C36:0	36	0	2	1	1.35 (1.07, 1.70)	1.39 (1.10, 1.75)	1.60 (1.28, 2.00)	<0.001	1.17 (1.08, 1.26)	<0.001
SM C38:0	38	0	2	1	1.37 (1.07, 1.76)	1.50 (1.17, 1.92)	1.69 (1.32, 2.16)	<0.001	1.18 (1.10, 1.26)	<0.001
SM C40:0	40	0	2	1	1.08 (0.84, 1.38)	1.27 (1.00, 1.62)	1.56 (1.23, 1.97)	<0.001	1.14 (1.07, 1.22)	<0.001
SM C34:1	34	1	2	1	1.20 (0.93, 1.56)	1.41 (1.09, 1.82)	1.64 (1.26, 2.13)	<0.001	1.18 (1.09, 1.27)	<0.001
SM C36:1	36	1	2	1	0.89 (0.71, 1.13)	1.05 (0.84, 1.31)	1.23 (1.00, 1.52)	0.01	1.17 (1.08, 1.26)	<0.001
SM C42:3	42	3	2	1	1.06 (0.83, 1.36)	1.24 (0.97, 1.57)	1.40 (1.10, 1.79)	0.002	1.14 (1.06, 1.23)	<0.001
SM (2OH) C34:1	34	1	4	1	1.24 (0.97, 1.59)	1.53 (1.20, 1.94)	1.65 (1.30, 2.08)	<0.001	1.21 (1.12, 1.30)	<0.001
SM (OH) C38:3	38	3	3	1	1.25 (0.97, 1.62)	1.38 (1.06, 1.80)	1.61 (1.24, 2.09)	<0.001	1.19 (1.09, 1.29)	<0.001
HexCer(d18:1/20:1)	38	2	2	1	1.05 (0.84, 1.32)	1.22 (0.98, 1.53)	1.46 (1.18, 1.80)	<0.001	1.17 (1.08, 1.26)	<0.001

Model was adjusted for age, sex, region (Beijing or Shanghai), residence (urban or rural), educational attainment (0–6 years, 7–9 years, or ≥10 years), current smoking (yes or no), current alcohol drinking (yes or no), physical activity (low, moderate, or high), family history of diabetes (yes or no), and BMI. Only sphingolipids associated with incident T2D after Bonferroni correction for multiple testing of the 76 metabolites are shown.

-OH, hydroxyl; Cer, ceramide; db, double bond; HexCer, hexosylceramide; No., number; RR, relative risk; SM, sphingomyelin; SM (OH), hydroxyl-sphingomyelin with 1 additional hydroxyl; SM (2OH), hydroxyl-sphingomyelin with 2 additional hydroxyls; T2D, type 2 diabetes.

In a sensitivity analysis of the log-Poisson model, longitudinal associations between the 14 sphingolipids and incident T2D were not materially altered (RR_per SD_: 1.11–1.22; all *P* < 0.001) by additionally including HbA_1c_ ≥ 6.5% to define T2D (S4 Fig). In stratified analyses, the associations were slightly stronger in individuals with overweight or obesity (BMI ≥ 24 kg/m^2^) and nonsmokers. No significant interactions were observed when analyses were stratified by sex, smoking, alcohol drinking, physical activity, or BMI (all *P*_interaction_ > 0.05; S5 Table).

### Sphingolipids network analysis

**Fig 1:**
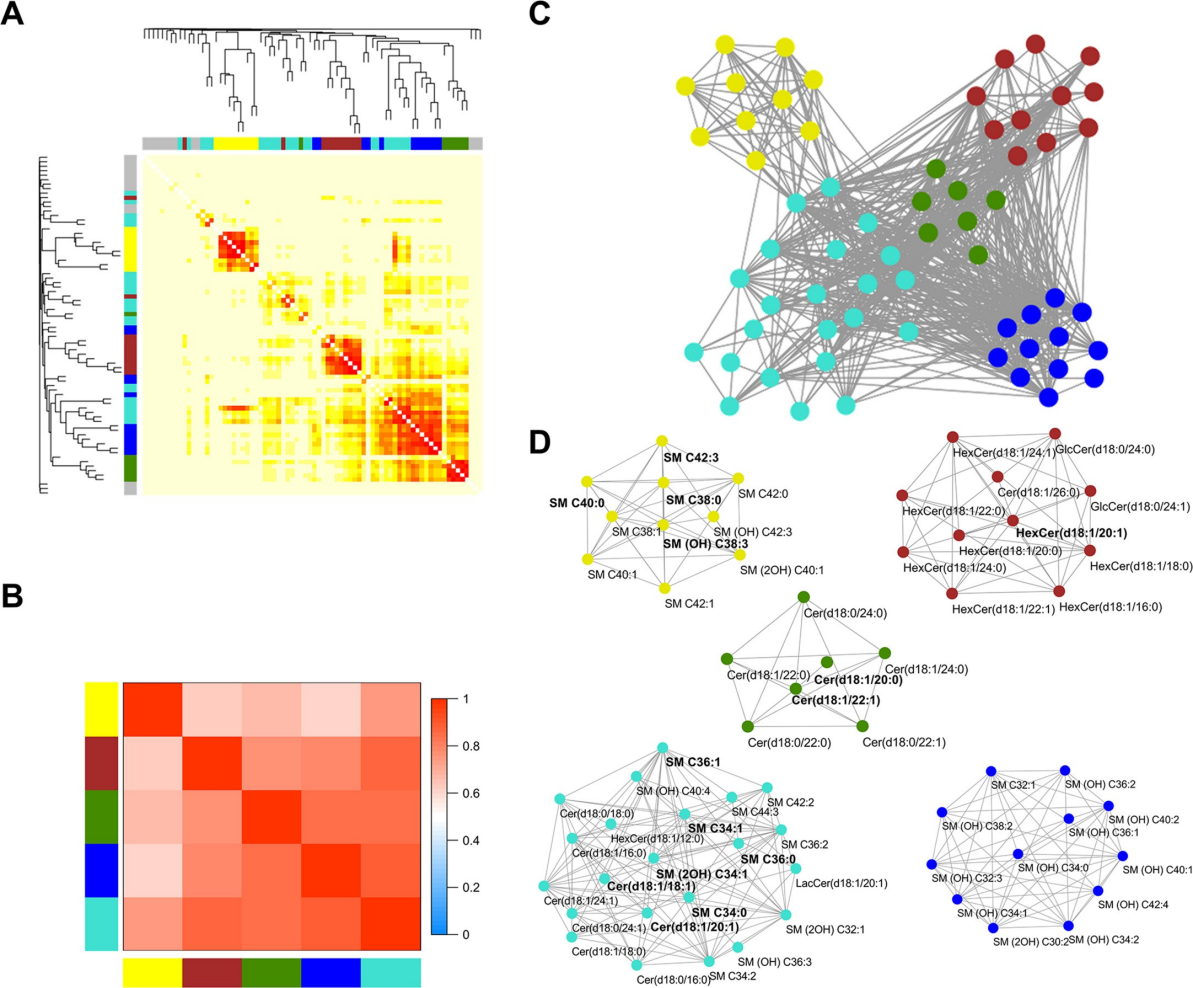
Weighted gene co-expression network analysis (WGCNA) of sphingolipid profile. (A) Cluster dendrogram and heatmap correlation of 76 sphingolipids. Color bars on the left and at the top represent 5 identified modules. Sphingolipids that could not be clustered to any 1 module are labeled gray. (B) Correlation heatmap among the 5 modules. Color bars on the left and at the bottom represent modules, and grid squares indicate Pearson correlation coefficients among module eigengenes. (C) Colors indicate the modules detected by topological overlap measure (yellow: very long-chain SMs, including saturated SMs; turquoise: long-chain Cers and SMs, including saturated SMs; green: very-long-chain Cers and dhCers; brown: GSLs; blue: hydroxyl-SMs). (D) Five sub-networks are shown as node and edge graphs. Sphingolipids are presented as nodes. Within each module, those nodes significantly associated with incident type 2 diabetes are labeled bold. Cer, ceramide; dhCer, dihydroceramide; GlcCer, glucosylceramide; GSL, glycosphingolipid; HexCer, hexosylceramide; LacCer, lactosylceramide; SM, sphingomyelin; SM (OH), hydroxyl-sphingomyelin with 1 additional hydroxyl; SM (2OH), hydroxyl-sphingomyelin with 2 additional hydroxyls.

WGCNA analysis generated 5 modules: module yellow contained very-long-chain SMs, module turquoise was composed of long-chain Cers and SMs, module green included very-long-chain Cers and dhCers, module brown comprised GSLs, and module blue consisted of hydroxyl-SMs (Fig 1). As shown in S5 Fig, all modules were positively correlated with fasting glucose (*r*_s_ = 0.44 to 0.51; all *P* < 0.05). Compared with the lowest quartile, the highest quartile of all modules except blue significantly increased the risk of incident T2D by percentages that ranged from 30% to 59%. Among them, the modules turquoise and yellow, containing saturated SMs, showed the highest associations with increased T2D risk (both *P* < 0.001; Table 3). Similarly as for individual sphingolipids, the significant associations between the 4 modules yellow, turquoise, green, and brown and incident T2D were abolished when fasting glucose was additionally adjusted for in the model (S4 Table).

**Table 3:** Associations (RRs [95% CIs]) between module eigengenes and incident T2D.

Module	Quartiles of module eigengenes				*P*_trend_	RR per SD	*P* value
	Q1	Q2	Q3	Q4			
**Module yellow (very-long-chain SMs, including saturated SMs); number of molecules = 10**							
Cases/total	86/493	111/494	145/494	187/493			
Model 1	1	1.26 (0.97, 1.63)	1.58 (1.24, 2.02)	1.85 (1.45, 2.37)	<0.001	1.21 (1.13, 1.29)	<0.001
Model 2	1	1.16 (0.90, 1.50)	1.40 (1.09, 1.79)	1.59 (1.24, 2.04)	<0.001	1.15 (1.07, 1.24)	<0.001
**Module turquoise (long-chain Cers and SMs, including saturated SMs); number of molecules = 23**							
Cases/total	97/493	102/494	145/494	185/493			
Model 1	1	0.97 (0.76, 1.25)	1.29 (1.02, 1.62)	1.60 (1.27, 2.00)	<0.001	1.23 (1.14, 1.34)	<0.001
Model 2	1	0.97 (0.76, 1.24)	1.22 (0.97, 1.54)	1.43 (1.14, 1.79)	<0.001	1.18 (1.09, 1.27)	<0.001
**Module green (very-long-chain Cers and dhCers); number of molecules = 7**							
Cases/total	91/493	110/494	139/494	189/493			
Model 1	1	1.15 (0.88, 1.50)	1.36 (1.04, 1.78)	1.78 (1.36, 2.34)	<0.001	1.23 (1.12, 1.35)	<0.001
Model 2	1	1.08 (0.83, 1.41)	1.20 (0.91, 1.58)	1.52 (1.16, 2.00)	<0.001	1.16 (1.06, 1.27)	0.002
**Module brown (GSLs); number of molecules = 11**							
Cases/total	105/493	110/494	149/494	165/493			
Model 1	1	0.99 (0.78, 1.25)	1.23 (0.98, 1.53)	1.29 (1.03, 1.62)	0.006	1.13 (1.04, 1.22)	0.003
Model 2	1	1.01 (0.80, 1.27)	1.26 (1.01, 1.56)	1.30 (1.05, 1.63)	0.004	1.13 (1.05, 1.22)	0.002
**Module blue (hydroxyl-SMs); number of molecules = 12**							
Cases/total	103/493	127/494	135/494	164/493			
Model 1	1	1.05 (0.83, 1.34)	1.03 (0.80, 1.34)	1.19 (0.92, 1.55)	0.19	1.11 (1.01, 1.22)	0.03
Model 2	1	1.02 (0.81, 1.29)	1.00 (0.78, 1.28)	1.09 (0.85, 1.41)	0.50	1.08 (0.98, 1.18)	0.11

Model 1 adjusted for age, sex, region (Beijing or Shanghai), residence (urban or rural), educational attainment (0–6 years, 7–9 years, or ≥10 years), current smoking (yes or no), current alcohol drinking (yes or no), physical activity (low, moderate, or high), and family history of diabetes (yes or no). Model 2 further adjusted for BMI.

Cer, ceramide; dhCer, dihydroceramide; GSL, glycosphingolipid; hydroxyl-SM, hydroxyl-sphingomyelin; RR, relative risk; SM, sphingomyelin; T2D, type 2 diabetes.

### HOMA-B-mediated detrimental associations of sphingolipids with T2D

Thirteen out of the 14 abovementioned sphingolipids were inversely associated with HOMA-B (all *P* < 0.001; S6 Table). Notably, adjustment for HOMA-B abolished significant associations for 10 of the sphingolipids, while substituting HOMA-B with HOMA-IR also eliminated significant associations for 5 of the sphingolipids (S4 Table). Mediation analysis further confirmed that significant associations of the 13 sphingolipids with T2D were largely mediated by HOMA-B (mediation proportion: 11.19%–42.42%; Table 4), rather than HOMA-IR, hsCRP, and adiponectin (S7 Table). Consistently, the module–T2D associations were also largely mediated by HOMA-B (mediation proportion: 20.00%–36.84%).

**Table 4:** Multiple mediation models of β-cell function and insulin resistance in the associations between sphingolipids and incident T2D.

Sphingolipid or module	SP on mediator (X→M)	*P* value	Direct effect (X→Y, adjusted M)	*P* value	Indirect effect (X→M→Y)	*P* value	Proportion mediated (%)
**HOMA-B**							
Cer(d18:1/18:1)	−0.096 (−0.139, −0.054)	<0.001	0.159 (0.051, 0.267)	0.004	0.030 (0.015, 0.050)	<0.001	15.87
Cer(d18:1/20:0)	−0.132 (−0.174, −0.090)	<0.001	0.146 (0.038, 0.253)	0.008	0.040 (0.023, 0.064)	<0.001	21.51
Cer(d18:1/20:1)	−0.174 (−0.216, −0.132)	<0.001	0.183 (0.075, 0.292)	<0.001	0.051 (0.030, 0.077)	<0.001	21.79
Cer(d18:1/22:1)	−0.210 (−0.257, −0.162)	<0.001	0.156 (0.034, 0.279)	0.01	0.062 (0.039, 0.092)	<0.001	28.44
SM C34:0	−0.281 (−0.328, −0.235)	<0.001	0.114 (−0.011, 0.238)	0.07	0.084 (0.049, 0.123)	<0.001	42.42
SM C36:0	0.011 (−0.033, 0.054)	0.64	0.223 (0.111, 0.334)	<0.001	−0.004 (−0.020, 0.011)	0.64	0.00
SM C38:0	−0.181 (−0.225, −0.136)	<0.001	0.196 (0.086, 0.307)	<0.001	0.053 (0.032, 0.079)	<0.001	21.29
SM C40:0	−0.101 (−0.145, −0.056)	<0.001	0.165 (0.056, 0.274)	0.003	0.031 (0.016, 0.048)	<0.001	15.82
SM C34:1	−0.277 (−0.324, −0.229)	<0.001	0.167 (0.044, 0.290)	0.008	0.079 (0.048, 0.115)	<0.001	32.11
SM C36:1	−0.193 (−0.236, −0.149)	<0.001	0.165 (0.050, 0.280)	0.005	0.057 (0.034, 0.087)	<0.001	25.68
SM C42:3	−0.101 (−0.148, −0.053)	<0.001	0.169 (0.051, 0.287)	0.005	0.031 (0.015, 0.052)	0.001	15.50
SM (2OH) C34:1	−0.098 (−0.142, −0.054)	<0.001	0.238 (0.124, 0.352)	<0.001	0.030 (0.014, 0.050)	<0.001	11.19
SM (OH) C38:3	−0.194 (−0.243, −0.144)	<0.001	0.189 (0.062, 0.317)	0.004	0.057 (0.035, 0.083)	<0.001	23.17
HexCer(d18:1/20:1)	−0.228 (−0.270, −0.186)	<0.001	0.156 (0.046, 0.266)	0.005	0.065 (0.038, 0.094)	<0.001	29.41
Module yellow	−0.082 (−0.108, −0.055)	<0.001	0.172 (0.058, 0.286)	0.003	0.043 (0.025, 0.068)	<0.001	20.00
Module turquoise	−0.131 (−0.156, −0.107)	<0.001	0.165 (0.049, 0.282)	0.005	0.067 (0.040, 0.098)	<0.001	28.88
Module green	−0.090 (−0.120, −0.061)	<0.001	0.150 (0.018, 0.282)	0.03	0.049 (0.027, 0.078)	<0.001	24.62
Module brown	−0.118 (−0.143, −0.094)	<0.001	0.108 (−0.005, 0.221)	0.06	0.063 (0.039, 0.092)	<0.001	36.84
**HOMA-IR**							
Cer(d18:1/18:1)	0.066 (0.025, 0.108)	0.002	0.175 (0.068, 0.282)	0.001	0.010 (0.002, 0.026)	0.05	5.41
Cer(d18:1/20:0)	0.025 (−0.017, 0.066)	0.25	0.180 (0.073, 0.286)	0.001	0.004 (−0.003, 0.016)	0.31	2.17
Cer(d18:1/20:1)	0.047 (0.005, 0.089)	0.03	0.226 (0.119, 0.332)	<0.001	0.007 (0.0004, 0.024)	0.10	3.00
Cer(d18:1/22:1)	0.099 (0.052, 0.147)	<0.001	0.203 (0.082, 0.324)	0.001	0.015 (0.004, 0.035)	0.03	6.88
SM C34:0	0.072 (0.025, 0.120)	0.003	0.190 (0.070, 0.311)	0.002	0.011 (0.002, 0.028)	0.05	5.47
SM C36:0	0.101 (0.058, 0.144)	<0.001	0.201 (0.090, 0.313)	<0.001	0.015 (0.002, 0.032)	0.03	6.94
SM C38:0	0.144 (0.100, 0.188)	<0.001	0.226 (0.116, 0.335)	<0.001	0.020 (0.004, 0.041)	0.03	8.13
SM C40:0	0.168 (0.124, 0.212)	<0.001	0.171 (0.061, 0.281)	0.002	0.024 (0.004, 0.049)	0.03	12.31
SM C34:1	0.133 (0.086, 0.181)	<0.001	0.226 (0.107, 0.346)	<0.001	0.019 (0.002, 0.040)	0.03	7.76
SM C36:1	0.052 (0.009, 0.095)	0.02	0.213 (0.101, 0.326)	<0.001	0.008 (0.0004, 0.022)	0.09	3.62
SM C42:3	0.134 (0.087, 0.180)	<0.001	0.177 (0.058, 0.295)	0.003	0.020 (0.003, 0.040)	0.04	10.15
SM (2OH) C34:1	0.073 (0.030, 0.116)	0.001	0.256 (0.142, 0.370)	<0.001	0.011 (0.002, 0.026)	0.05	4.12
SM (OH) C38:3	0.164 (0.115, 0.213)	<0.001	0.226 (0.099, 0.352)	<0.001	0.023 (0.004, 0.050)	0.03	9.24
HexCer(d18:1/20:1)	0.060 (0.018, 0.103)	0.005	0.211 (0.104, 0.318)	<0.001	0.009 (0.001, 0.024)	0.06	4.09
Module yellow	0.090 (0.066, 0.114)	<0.001	0.189 (0.075, 0.303)	0.001	0.024 (0.003, 0.05)	0.03	11.27
Module turquoise	0.037 (0.013, 0.060)	0.002	0.221 (0.107, 0.335)	<0.001	0.011 (0.002, 0.024)	0.05	4.74
Module green	0.066 (0.039, 0.093)	<0.001	0.179 (0.047, 0.311)	0.008	0.019 (0.005, 0.040)	0.02	9.60
Module brown	0.023 (0.000, 0.046)	0.05	0.165 (0.054, 0.276)	0.004	0.007 (0.001, 0.020)	0.12	4.07

Model was adjusted for age, sex, region (Beijing or Shanghai), residence (urban or rural), educational attainment (0–6 years, 7–9 years, or ≥10 years), current smoking (yes or no), current alcohol drinking (yes or no), physical activity (low, moderate, or high), family history of diabetes (yes or no), and BMI. In the model, X denotes the sphingolipid, M denotes the mediator, and Y denotes incident T2D.

Cer, ceramide; HexCer, hexosylceramide; HOMA-B, homeostatic model assessment of β-cell function; HOMA-IR, homeostatic model assessment of insulin resistance; SM, sphingomyelin; SM (OH), hydroxyl-sphingomyelin with 1 additional hydroxyl; SM (2OH), hydroxyl-sphingomyelin with 2 additional hydroxyls; SP, sphingolipid; T2D, type 2 diabetes.

**Fig 2:**
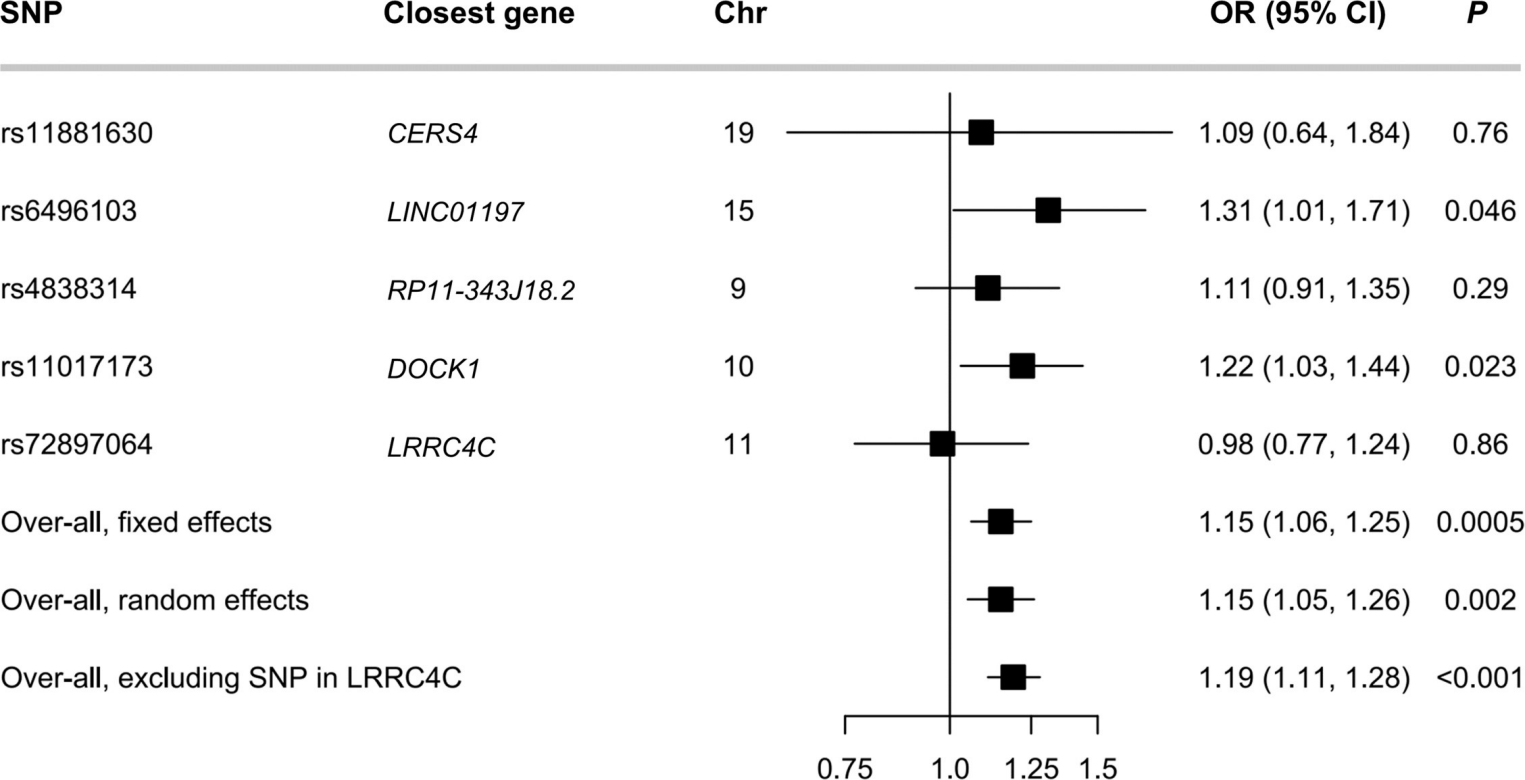
Association between genetically instrumented level of Cer(d18:1/20:1) and type 2 diabetes. Odds ratio (OR) was scaled genetically per 1-SD increase in plasma Cer(d18:1/20:1) level. Estimated effect size was calculated by inverse variance weighting (IVW) analysis using a fixed-effects or random-effects model. *P* for heterogeneity between estimates from individual SNPs was 0.28 in the analysis including all 5 SNPs and 0.51 in the analysis excluding rs72897064 near *LRRC4C*. Chr, chromosome; SNP, single nucleotide polymorphism.

In addition, GWASs for the 14 sphingolipids were performed among 1,976 participants of Chinese ancestry. Significant genome-wide associations were identified for Cer(d18:1/20:0) and Cer(d18:1/20:1) at *CERS4*, for SM C34:0 at *LASP1*, for SM C34:1 at *PCDHGA12*, for SM C36:0 at *LRRC4C*, for SM C42:3 at *MYRF*, and for HexCer(d18:1/20:1) at *ATP10D* (S8 Table; S6 Fig). Five significant SNPs (including 1 genome-wide significant and 4 less stringently significant SNPs) for Cer(d18:1/20:1) were obtained, and the mean *F*-statistic was 24 (S8 Table). The odds ratio for T2D per SD of genetically increased Cer(d18:1/20:1) was 1.15 (95% CI 1.05–1.26; *P* = 0.002). No strong evidence of heterogeneity and unbalanced pleiotropy existed (*P*_heterogeneity_ = 0.28; intercept = −0.003, *P* = 0.80) (Fig 2; S9 Table). In a leave-one-out sensitivity analysis, the effect size of Cer(d18:1/20:1) on T2D ranged from 1.12 to 1.19, showing small fluctuation (S7 Fig). However, nonsignificant associations with T2D were detected for other sphingolipids, including Cer(d18:1/20:0), SMs (C34:0, C34:1, C36:0, C42:3), and HexCer(d18:1/20:1).

## Discussion

By applying a high-coverage targeted lipidomic approach to measure 76 sphingolipids out of 728 lipids, we observed that 11 novel and 3 previously reported sphingolipids were significantly associated with elevated risk of T2D, independent of established risk factors. The deleterious associations of sphingolipids with T2D were largely mediated by β-cell function, and a positive association between genetically instrumented Cer(d18:1/20:1) and T2D was also evidenced.

To the best of our knowledge, this is the first study to show such a large number of T2D-associated sphingolipid species, including 11 novel and 3 reported sphingolipids, implying that sphingolipid perturbations could precede and precipitate the onset of T2D. Of the 14 identified sphingolipids, we confirmed the positive associations of SM C34:1 and SM C36:1 with T2D incidence as reported in a Singapore Chinese population [13], and of Cer(d18:1/20:0) with T2D incidence as reported in a French population [28]. Beyond the associations for individual species, our network analysis further supported collective effects: 4 modules containing SMs, Cers, and GSLs were positively associated with T2D incidence, with the strongest associations in the 2 modules containing saturated SMs. To date, only limited longitudinal studies have investigated sphingolipid–T2D associations, and these were mainly conducted in European and American populations and covered 4–15 sphingolipid species [12,14,15], except the Singapore study [13]. The species of Cer and SM studied were predominantly those carrying the d18:1 sphingoid base and a SFA, and yielded mixed findings [12,14,15]. For instance, when quantifying 4 Cers, Hilvo et al. reported a positive association of Cer(d18:1/18:0) with incident T2D in a Finnish population [15], whereas the Dallas Heart Study found null association in a multi-ethnic cohort [29]. Notably, only SM C34:1 and SM C36:1 out of 80 sphingolipids were found to be positively associated with T2D incidence in the Singapore study among 2,302 ethnically Chinese individuals [13]. Though both the Singapore study and our study were conducted in ethnically Chinese populations, the inconsistencies between the studies might be ascribed to the differences in methodologies, such as lipid extraction (liquid–liquid extraction versus protein precipitation extraction) and mass spectrometry setups, population characteristics (older individuals with higher levels of BMI, glucose, and T2D incidence in our study), statistical methods such as adjusted covariates, and dietary and lifestyle characteristics. While waiting for further studies, the high-coverage lipidomics used in our study certainly provide a unique opportunity to explore the associations of more novel sphingolipids with T2D development.

One of the important findings in our study was that monounsaturated Cers were significantly associated with elevated risk of T2D, rather than saturated Cers, as reported in Western populations [15,28]. The distinct fatty acids in Cers might reflect substrate abundancy during Cer synthesis. Presumably, higher intake of SFAs in Western populations than in Chinese populations (11.5%–11.9% versus 7.3%–7.5%) might enhance de novo synthesis of saturated Cers [28,30,31], while the high carbohydrate-to-fat ratio of the diet (60.8%:27.0%) in our cohort was correlated with upregulated levels of fatty acids in the de novo lipogenesis (DNL) pathway, mainly 16:1n-7, 16:1n-9, and 18:1n-9, which are significantly associated with incident metabolic syndrome and T2D [19]. Likewise, the plentiful DNL fatty acids might facilitate monounsaturated Cer synthesis, and this notion was further supported by positive correlations between baseline Cers and DNL fatty acids (*P* < 0.001; S8 Fig). Notably, we also documented a significant association between genetically instrumented Cer(d18:1/20:1) and T2D using the *CERS4* locus as a genetic instrument. *CERS4* encodes LAG1 longevity assurance homologue 4, which catalyzes sphinganine into dihydroceramide. A previous GWAS showed significant association between *CERS4* variants and Cer(d18:1/20:0) [32]. In addition, our study found that adjustment for baseline fasting glucose removed significant Cer–T2D associations, suggesting their glucose-dependent nature. Indeed, recent studies in mice indicated that decreased Cer levels improved glucose homeostasis after genetic ablation of dihydroceramide desaturase 1 (*DES1*) or ceramide synthase 6 (*CerS6*), 2 critical enzymes in the de novo Cer synthesis pathway [7,8]. Collectively, our results, including genetic analysis, comprehensively shed light on the detrimental associations of specific Cers with T2D, providing further supportive evidence for Cers as early biomarkers or intervention targets in glycemic control and T2D prevention.

Our study also highlighted another 2 subclasses, namely SMs (4 saturated, 3 unsaturated, and 2 hydroxyl-SMs) and GSLs (1 HexCer), that were significantly associated with elevated T2D incidence. As the most abundant subclass, SMs constitute roughly 87% of plasma sphingolipids and were reported to be associated with higher risks of atherosclerosis and coronary heart disease [5]. However, the SM–T2D associations remained controversial. For instance, both our study and the Singapore study found positive associations of SM C34:1 (or d16:1/18:0; S2 Text) and SM C36:1 (or d18:1/18:0) [13], while a German cohort (EPIC-Potsdam) and a Spanish cohort (PREDIMED Trial) showed inverse associations of SM(d18:1/16:1) and a SM score derived from d18:1 SMs with T2D incidence [12,14]. These discrepancies could be explained by the following: (1) distinct SM structures (saturated SMs in our study versus unsaturated SMs in Western populations) that may exert distinctive impacts on T2D-related traits [16], (2) the lack of saturated SM and hydroxyl-SM data [12,14], or (3) effects of age, vitamin D, and obesity status [33]. For instance, Lemaitre et al. reported in an American cohort study that SMs (e.g., C36:1) were inversely associated with insulin, HOMA-IR, and HOMA-B in individuals with BMI ≤ 30 kg/m^2^, but the associations became positive with BMI >30 kg/m^2^ [11]. Accordingly, the mean BMI in the aforementioned studies ranged from 25.9 to 30.8 kg/m^2^, BMI values at which SMs were more likely to be associated with lower T2D risk [12,14,34]. In contrast, the mean BMI was 24.3 kg/m^2^ in our study population and 22.3 kg/m^2^ in the study of Singapore Chinese individuals, BMI values at which SMs were already associated with unfavorable metabolic traits, like increased levels of fasting glucose, HbA_1c_, and HOMA-IR. East Asian populations are known to have relatively higher accumulation of visceral fat even at a relatively lower BMI than white populations [3], which might partially elucidate the different SM–T2D associations between East Asian and Western populations. In terms of the effects of GSLs on T2D, studies in this regard were scarce owing to low circulating GSL concentrations [13,28]; nonetheless, our study evidenced a novel association between HexCer and incident T2D. Although the underlying mechanism is not entirely clear, studies in animal models showed that lowering GSL levels using a pharmacological inhibitor significantly improved glucose homeostasis [35]. Thus, the current findings suggested that the 2 subclasses SMs and GSLs were also associated with future T2D, and the effects of adiposity status on the associations for SMs may need to be addressed in multi-ethnic populations.

Interestingly, we also found that the detrimental sphingolipid–T2D associations appeared to be largely mediated by β-cell dysfunction instead of insulin resistance (mediation proportion 11.19%–42.42% for HOMA-B versus 2.17%–12.31% for HOMA-IR), although the sphingolipids in these detrimental sphingolipid–T2D associations were also positively associated with HOMA-IR, consistent with other studies [13,28]. In particular, this finding implies that the observed association between genetically instrumented Cer(d18:1/20:1) and T2D is possibly mediated by impaired β-cell function. Mechanistic studies in rodents revealed that stimulating de novo Cer synthesis promoted reduced β-cell function via enhancing reactive oxygen species production, mitochondrial dysfunction, and consequent β-cell apoptosis [36]. Interestingly, we also observed positive associations between Cers and acylcarnitines, which may reflect dysregulated fatty acid oxidation and mitochondrial stress (*P* < 0.001; S8 Fig). Our previous study showed that acylcarnitines were positively associated with incident T2D and considerably improved predictive ability for T2D [20]. SMs were found to be involved in β-cell apoptosis and regulating insulin secretion [37]. *Sphingomyelin synthase 2* (*SMS2*)–deficient mice had diminished plasma SM levels and attenuated nuclear factor kappa-B (NF-κB) activation, accompanied by improved insulin sensitivity and glucose tolerance [9]. Moreover, inhibiting GSL concentrations in Zucker rats with diabetes prevented β-cell function failure and improved glycemic control [35]. Though impaired β-cell function and insulin resistance are the 2 major causes in the pathogenesis of T2D, East Asian populations tend to display relatively lower β-cell function and insufficient insulin secretion, so they are considered as having higher T2D predisposition than white populations [3]. Overall, our findings highlighted a stronger mediation effect of β-cell dysfunction than of insulin resistance in the sphingolipid–T2D associations in Chinese individuals (S9 Fig).

The strengths of our study included the following: a prospective cohort design, collection of a multitude of covariates to minimize confounding, application of a high-coverage targeted lipidomic approach for a wide range of sphingolipids, and application of multi-omics to underscore potential mechanisms underlying sphingolipid–T2D associations. Admittedly, there were also some limitations. First, most of incident T2D cases were diagnosed by fasting glucose in the follow-up visit; therefore, potentially undiagnosed cases may exist, and more detailed information on the timing of the onset of T2D during the 6-year follow-up period could not be obtained. Second, our findings could not be validated in an independent population, although we conducted a cross-region validation and obtained similar associations in Beijing and Shanghai, subpopulations with somewhat different genetic background and lifestyle characteristics (S5 Table) [38]. Third, about 23% of participants were lost in the follow-up period, though this rate was comparable with that of other cohort studies [39,40]. Fourth, due to its observational nature, our study cannot make causal inference for most of the identified sphingolipids. Although we conducted a MR analysis and found a positive association of genetically predicted Cer(d18:1/20:1) with T2D, replication in future studies is warranted, given the relatively small number of summary-level data for the associations between genetic variants and sphingolipids in the current study. Finally, our findings from the middle-aged and elderly Chinese population may not be generalizable to other age and ethnic populations.

In conclusion, we identified a panel of novel sphingolipids with unique structures that were significantly associated with elevated risk of T2D, and these associations appeared to be largely mediated through β-cell dysfunction among Chinese individuals. Our findings suggested that specific sphingolipids could be promising early biomarkers and intervention targets beyond traditional ones in T2D prevention and control in future clinical settings. Certainly, more studies are merited to confirm our findings and further identify their determinants and potential applications in other populations.

## Supporting information

S1 STROBE Checklist (DOCX)

S1 Fig Chemical structure of sphingolipids. For each graph, *n* denotes the number of sphingolipids identified in the current study. Sphingoid base denotes sphingosine, generally defined as a carbon chain, di-hydroxylated at positions 1 and 3 and with a double bond at position 4, which varies in chain length (C16–C19), number of double bonds (0–2), and number of hydroxyls (0–3). The fatty acid residue also varies substantially with respect to chain length (C14–C26), number of double bonds (saturated or unsaturated), and number of hydroxyl groups (0–1).
(TIF)

S2 Fig The Spearman correlations among baseline concentrations of sphingolipids. (TIF)

S3 Fig The Spearman correlations between sphingolipids and cardiometabolic traits adjusted for age, sex, region (Beijing or Shanghai), and residence (urban or rural). (TIF)

S4 Fig Sensitivity analysis of T2D incidence including HbA_1c_ ≥ 6.5% as T2D definition. M1: incident T2D was defined with self-reported doctor-diagnosed diabetes, taking antidiabetic medications, or fasting glucose ≥ 7.0 mmol/l. M2: further adding HbA_1c_ ≥ 6.5% to define T2D. Model was adjusted for age, sex, region (Beijing or Shanghai), residence (urban or rural), educational attainment (0–6 years, 7–9 years, or ≥10 years), current smoking (yes or no), current alcohol drinking (yes or no), physical activity (low, moderate, or high), family history of diabetes (yes or no), and BMI.
(TIF)

S5 Fig Weighted gene co-expression network analysis (WGCNA) of sphingolipid profile. Scale-free topology parameters in WGCNA are shown in (A) and (B). The smallest soft power (7) with *R*^2^ ≥ 0.80 was chosen. (C). Spearman correlations were calculated between module eigengenes and metabolic traits.
(TIF)

S6 Fig Manhattan plots and Q-Q plots for genome-wide associations with circulating sphingolipids (*n* = 1,976). The −log10 *P* values calculated using linear regression analysis under an additive genetic model are presented in the figure. (A) Cer(d18:1/20:0); (B) Cer(d18:1/20:1); (C) SM C34:0; (D) SM C36:0; (E) SM C34:1; (F) SM C42:3; (G) HexCer C20:1. The red lines in Manhattan plots represent genome-wide significant level (*P* < 5 × 10^−8^).
(TIF)

S7 Fig Forest plot of sensitivity analysis. (TIF)

S8 Fig The Spearman correlations of sphingolipids with acylcarnitines and fatty acids in the de novo lipogenesis pathway. (TIF)

S9 Fig Sphingolipid metabolism and underlying mechanisms in relation to T2D. Ceramides can be produced by (a) a de novo pathway initiated from serine and palmitoyl precursors, (b) synthesis via sphingosine-1-phosphate (S-1-P), namely the “salvage pathway,” and (c) degradation of sphingomyelins through sphingomyelinase. As important signal molecules, ceramides can (d) induce pancreatic β-cell apoptosis through increasing endoplasmic reticulum stress, producing reactive oxygen species, (e) promote the development of insulin resistance via activating either protein phosphatase 2 or protein kinase C ζ, leading to attenuated serine/threonine protein kinase, and (f) activate NLR family 3 inflammasome and produce more cytokines. Solid lines represent a 1-step or certain process, whereas dotted lines represent multiple-step or uncertain processes. CDase, ceramidase; CS, ceramide synthase; DES, dihydroceramide synthase; GlcCer, glucosylceramide; GCase, glucosylceramidase; GCS, glucosylceramide synthase; HexCer, hexosylceramide; LacCer, lactosylceramide; S1PP, S-1-P phosphatase; SK, sphingosine kinase; SMase, sphingomyelinase; SMS, sphingomyelin synthase; T2D, type 2 diabetes.
(TIF)

S1 Table Tandem mass spectrometry parameters for analyzing human plasma sphingolipids. (DOCX)

S2 Table Baseline sphingolipids in incident T2D cases and non-cases. (DOCX)

S3 Table Individual sphingolipids and risk of incident T2D. (DOCX)

S4 Table Conditional analysis of plasma sphingolipids with incident T2D. (DOCX)

S5 Table Stratified analyses of the associations between plasma sphingolipids and incident T2D. (DOCX)

S6 Table Associations of sphingolipids with insulin resistance, β-cell function, hsCRP, and adiponectin. (DOCX)

S7 Table Multiple mediation model of fasting glucose, hsCRP, and adiponectin in the associations between sphingolipids and incident T2D. (DOCX)

S8 Table Characteristics of genetic instruments for sphingolipids. (DOCX)

S9 Table Associations of genetically instrumented sphingolipids with T2D using Mendelian randomization. (DOCX)

S1 Text Analysis plan. (DOCX)

S2 Text Supplementary methods. (DOCX)

## Abbreviations

Cer: ceramide
dhCer: dihydroceramide
GSL: glycosphingolipid
GWAS: genome-wide association study
HbA_1c_: glycohemoglobin
HDL-C: high-density lipoprotein cholesterol
HexCer: hexosylceramide
HOMA-B: homeostatic model assessment of β-cell function
HOMA-IR: homeostatic model assessment of insulin resistance
hsCRP: high-sensitivity C-reactive protein
LDL-C: low-density lipoprotein cholesterol
MR: Mendelian randomization
MUFA: monounsaturated fatty acid
RR: relative risk
SFA: saturated fatty acid
SM: sphingomyelin
SNP: single nucleotide polymorphism
T2D: type 2 diabetes
TG: triglyceride
WGCNA: weighted gene co-expression network analysis
