# Supplementary figures and images for: Associations among circulating sphingolipids, β-cell function, and risk of developing type 2 diabetes: A population-based cohort study in China
Source: PLoS Med. 2020 Dec 9;17(12):e1003451. doi: 10.1371/journal.pmed.1003451 (PMC7725305; doi:10.1371/journal.pmed.1003451)

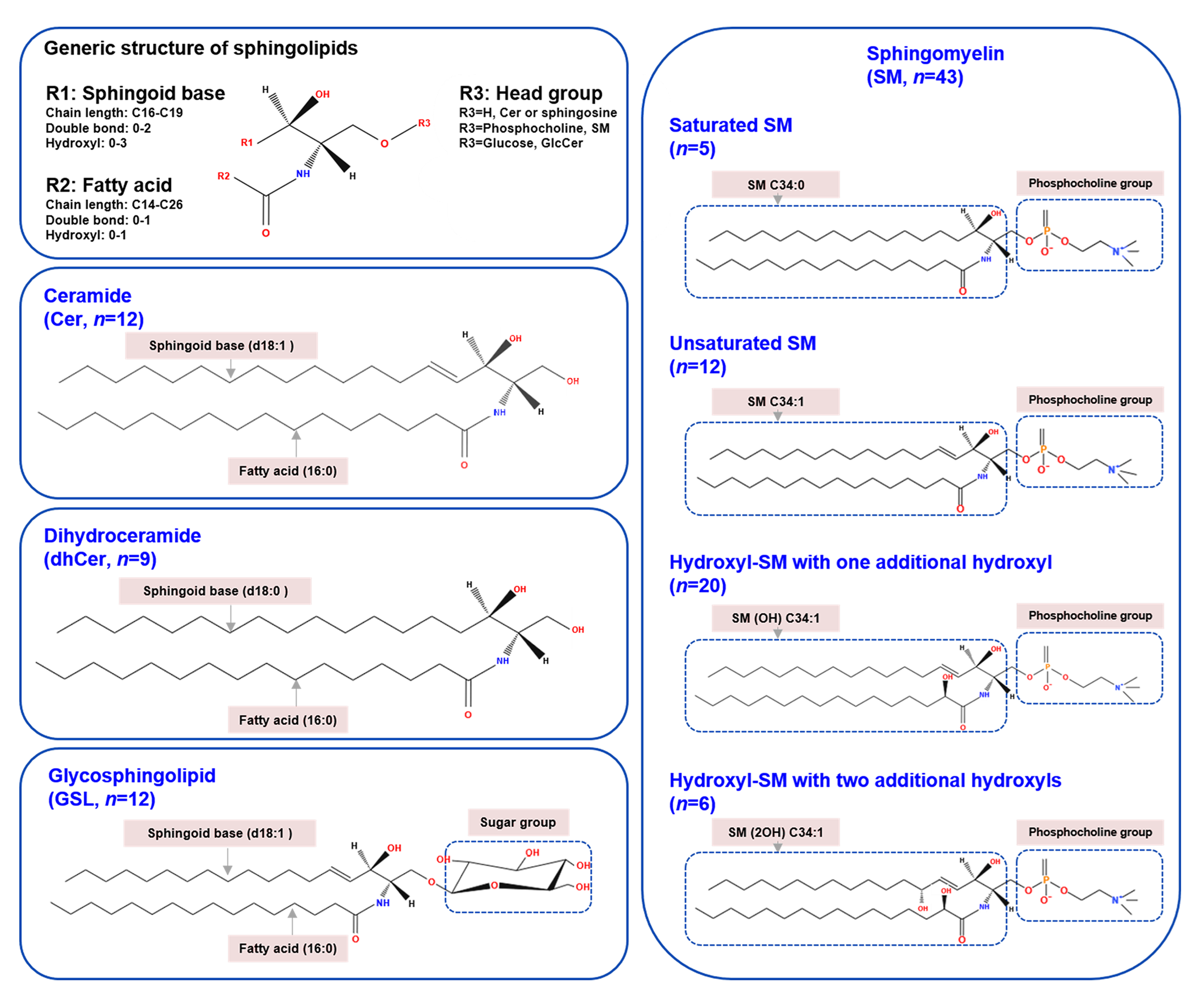

Supplement: S1 Fig — For each graph, n denotes the number of sphingolipids identified in the current study. Sphingoid base denotes sphingosine, generally defined as a carbon chain, di-hydroxylated at positions 1 and 3 and with a double bond at position 4, which varies in chain length (C16–C19), number of double bonds (0–2), and number of hydroxyls (0–3). The fatty acid residue also varies substantially with respect to chain length (C14–C26), number of double bonds (saturated or unsaturated), and number of hydroxyl groups (0–1). (TIF) [file pmed.1003451.s002.tif]

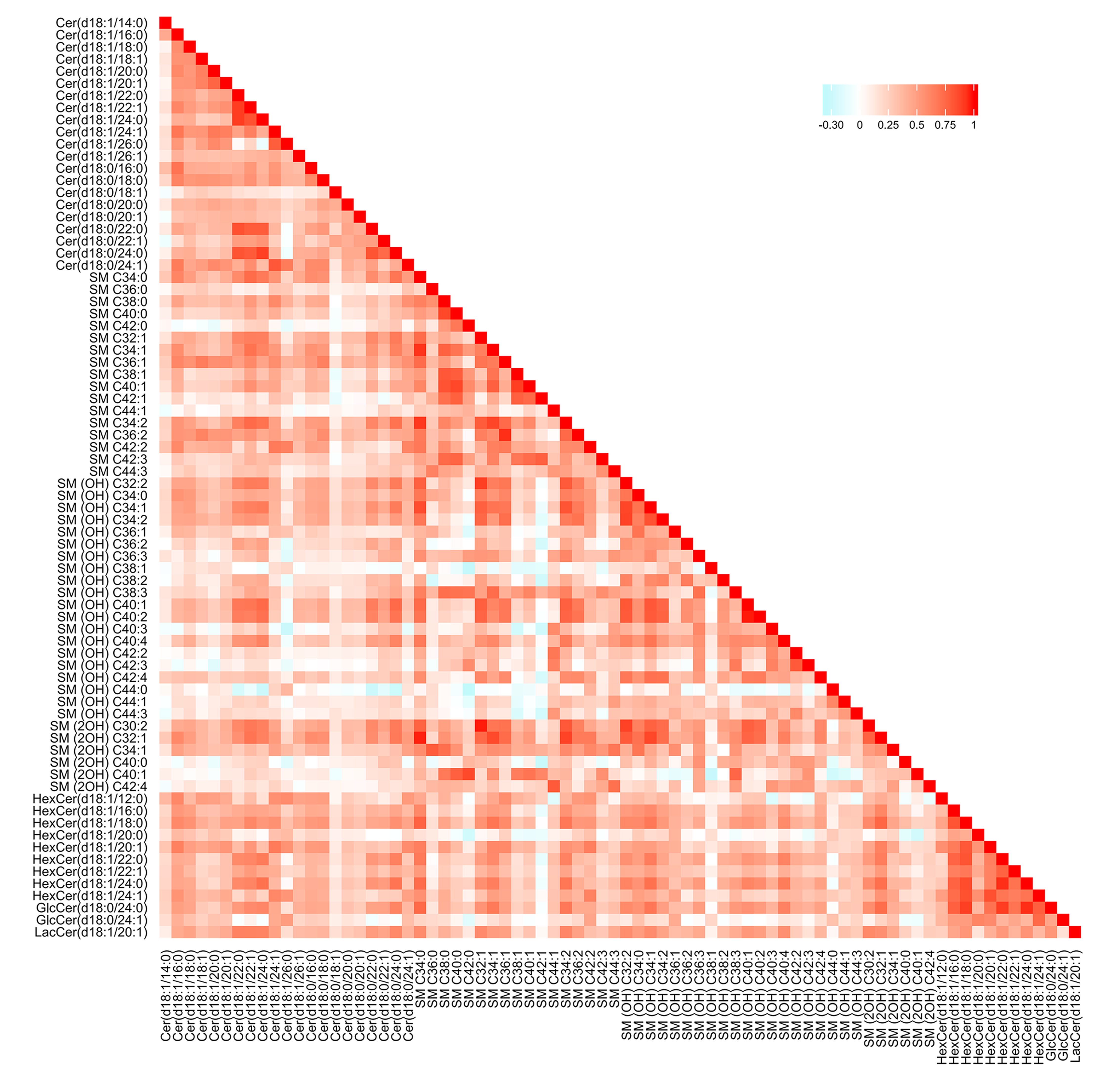

Supplement: S2 Fig — (TIF) [file pmed.1003451.s003.tif]

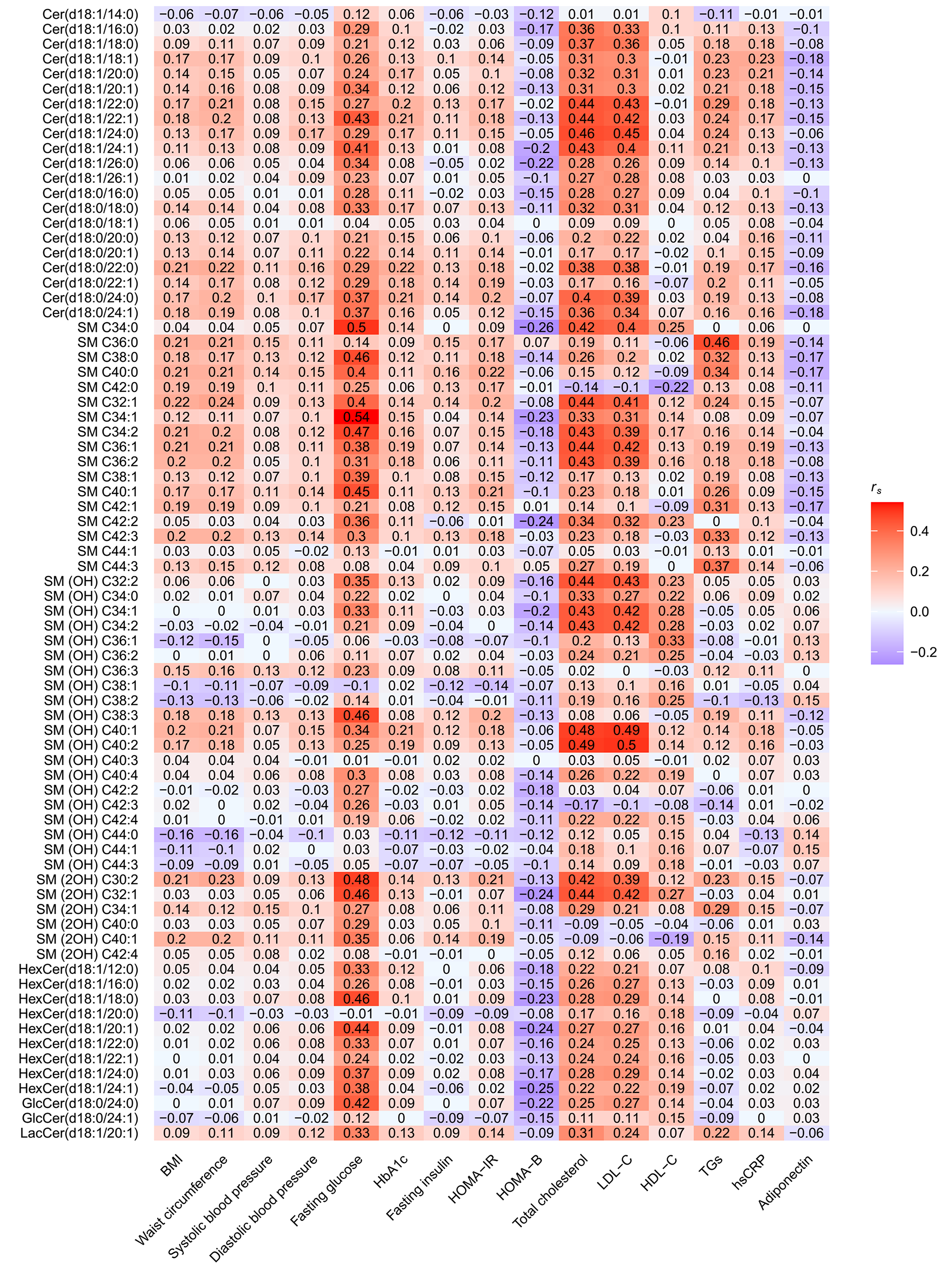

Supplement: S3 Fig — (TIF) [file pmed.1003451.s004.tif]

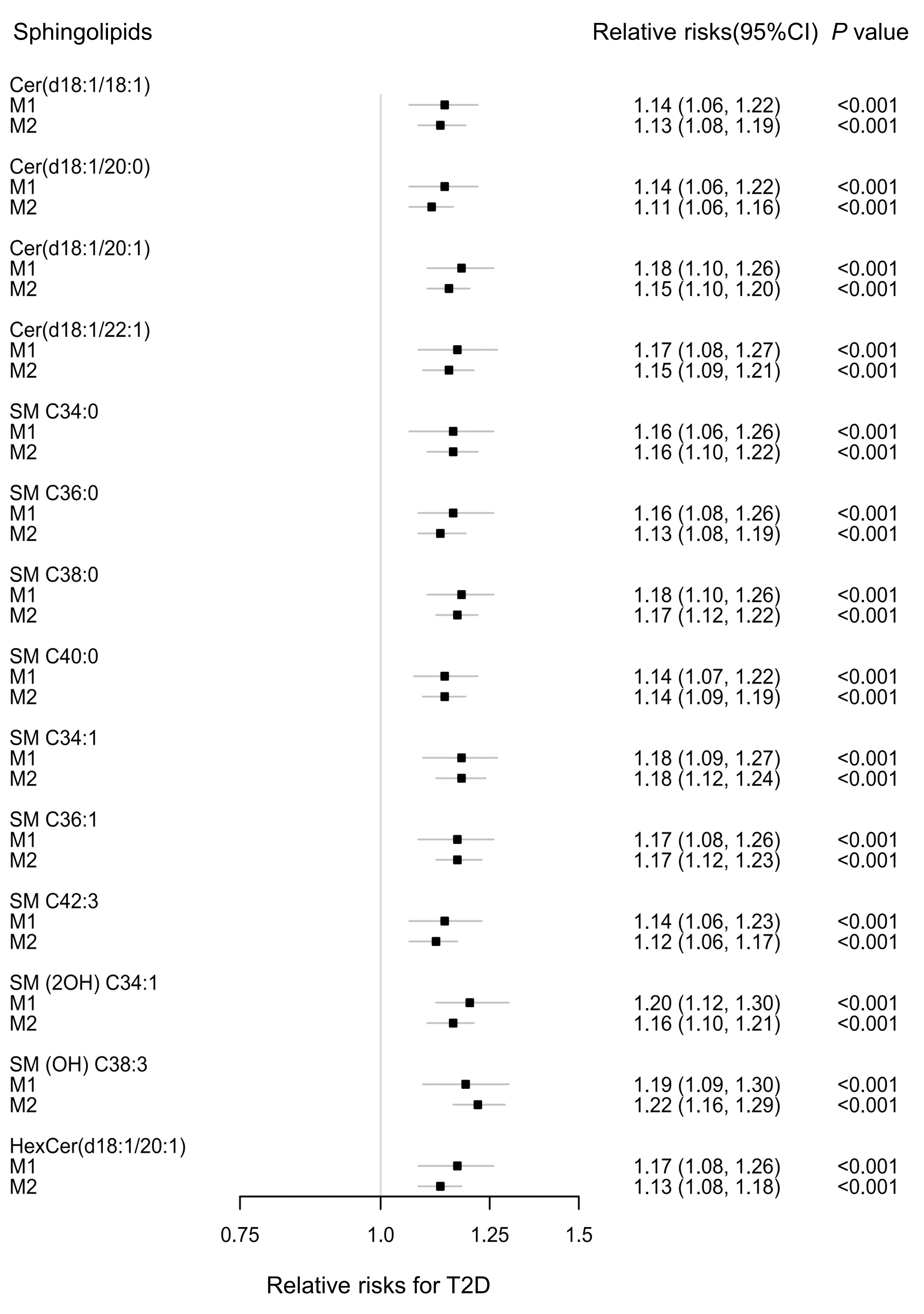

Supplement: S4 Fig — M1: incident T2D was defined with self-reported doctor-diagnosed diabetes, taking antidiabetic medications, or fasting glucose ≥ 7.0 mmol/l. M2: further adding HbA1c ≥ 6.5% to define T2D. Model was adjusted for age, sex, region (Beijing or Shanghai), residence (urban or rural), educational attainment (0–6 years, 7–9 years, or ≥10 years), current smoking (yes or no), current alcohol drinking (yes or no), physical activity (low, moderate, or high), family history of diabetes (yes or no), and BMI. (TIF) [file pmed.1003451.s005.tif]

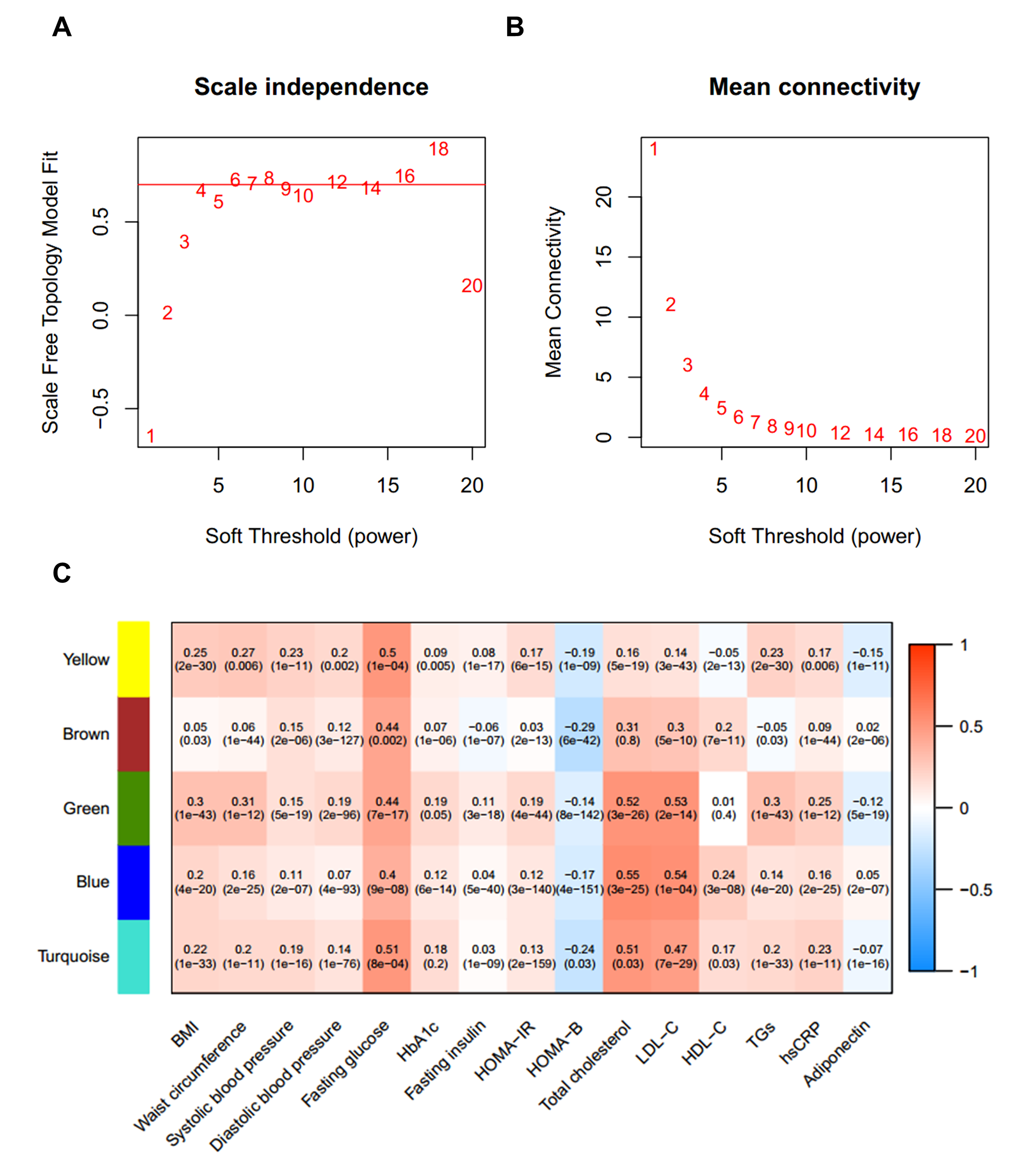

Supplement: S5 Fig — Scale-free topology parameters in WGCNA are shown in (A) and (B). The smallest soft power (7) with R2 ≥ 0.80 was chosen. (C). Spearman correlations were calculated between module eigengenes and metabolic traits. (TIF) [file pmed.1003451.s006.tif]

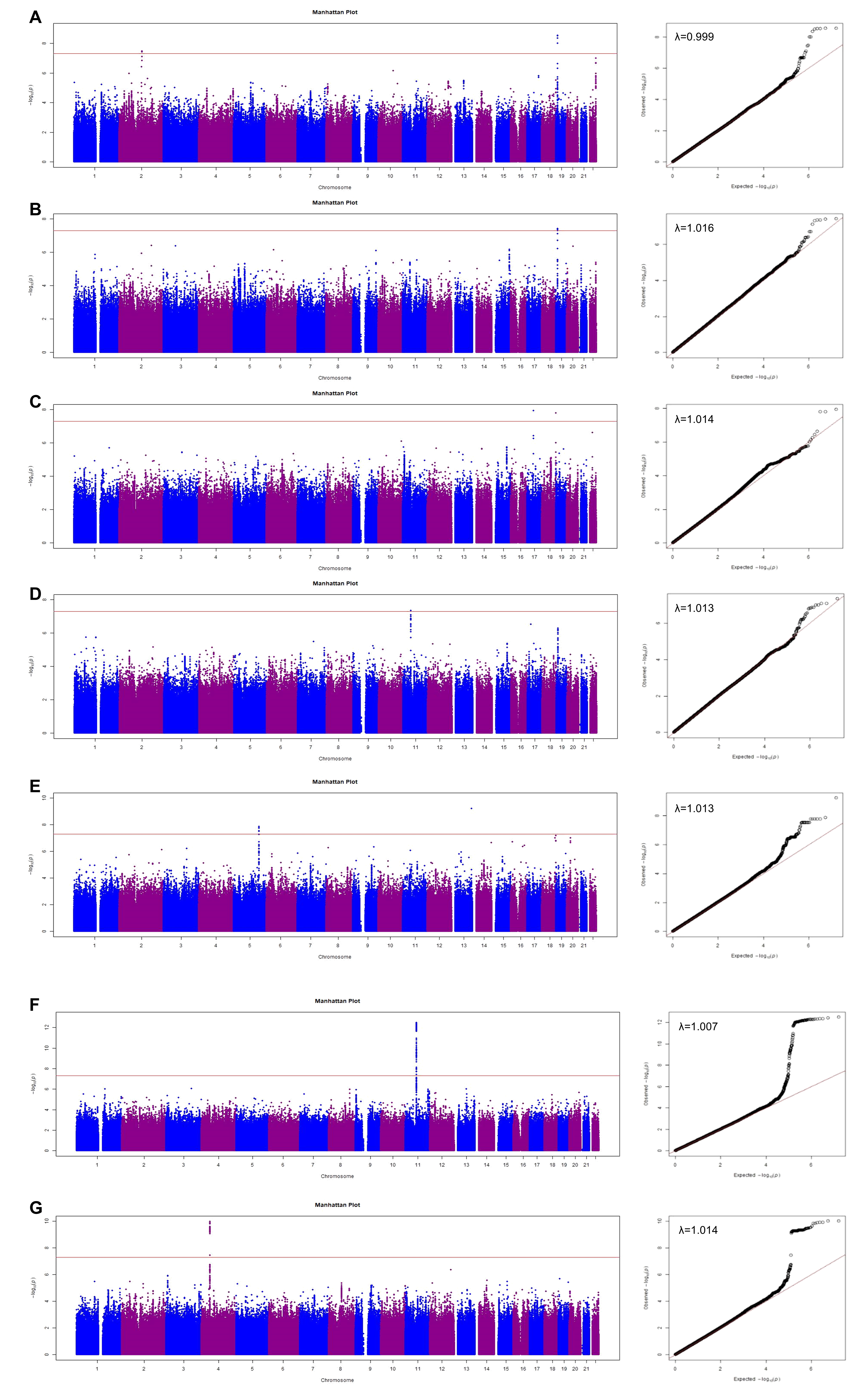

Supplement: S6 Fig — The −log10 P values calculated using linear regression analysis under an additive genetic model are presented in the figure. (A) Cer(d18:1/20:0); (B) Cer(d18:1/20:1); (C) SM C34:0; (D) SM C36:0; (E) SM C34:1; (F) SM C42:3; (G) HexCer C20:1. The red lines in Manhattan plots represent genome-wide significant level (P < 5 × 10^−8^). (TIF) [file pmed.1003451.s007.tif]

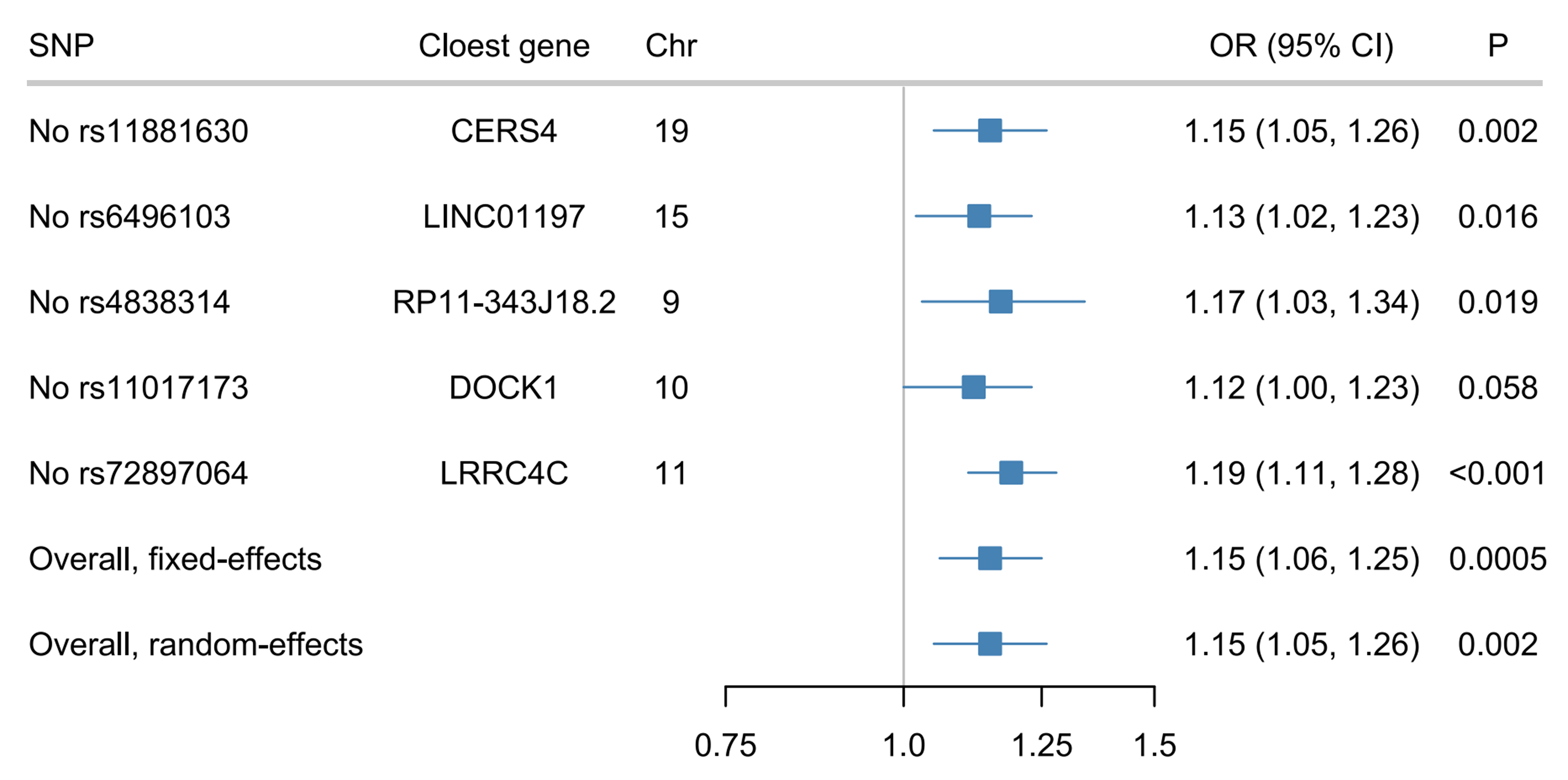

Supplement: S7 Fig — (TIF) [file pmed.1003451.s008.tif]

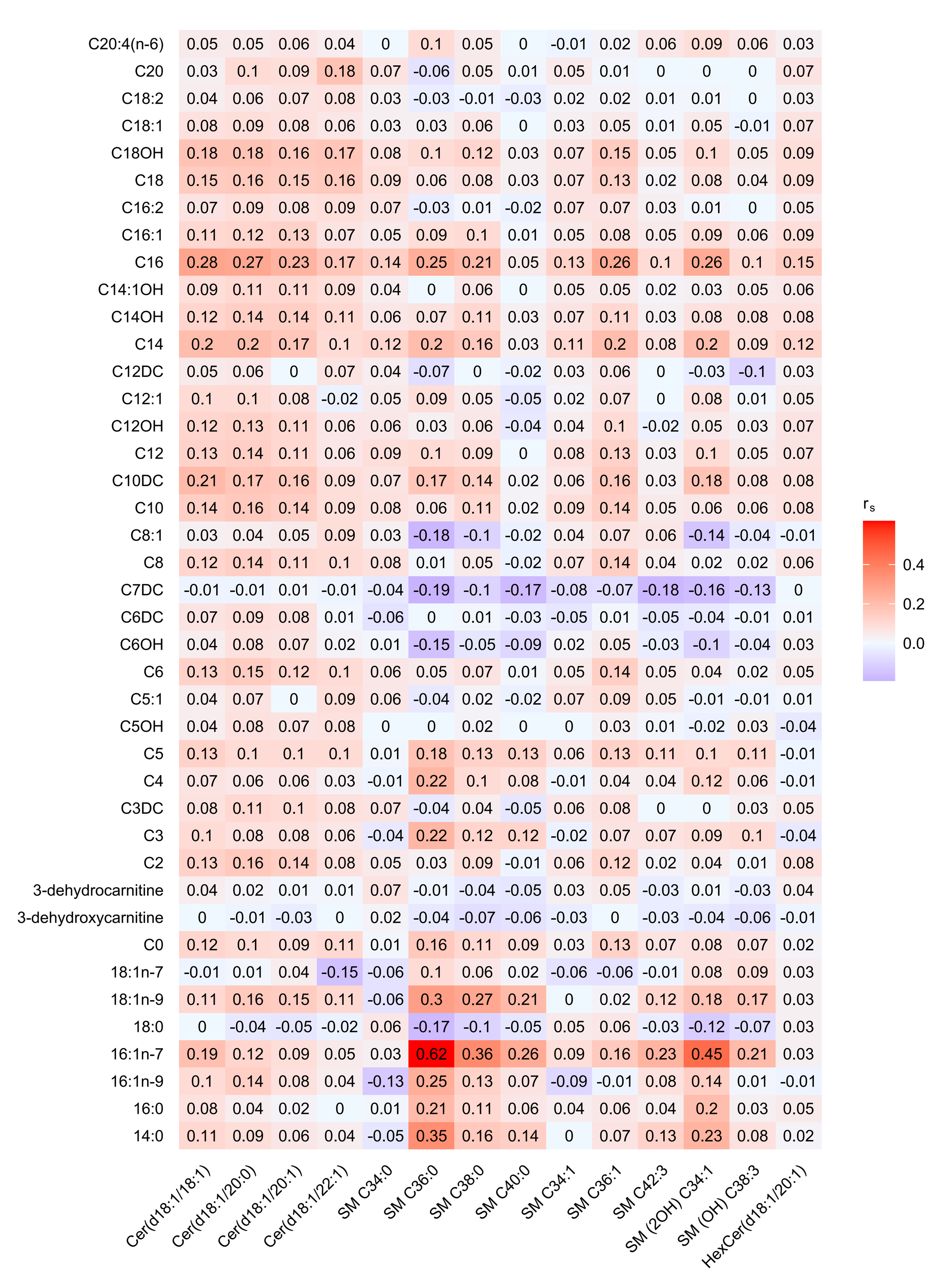

Supplement: S8 Fig — (TIF) [file pmed.1003451.s009.tif]

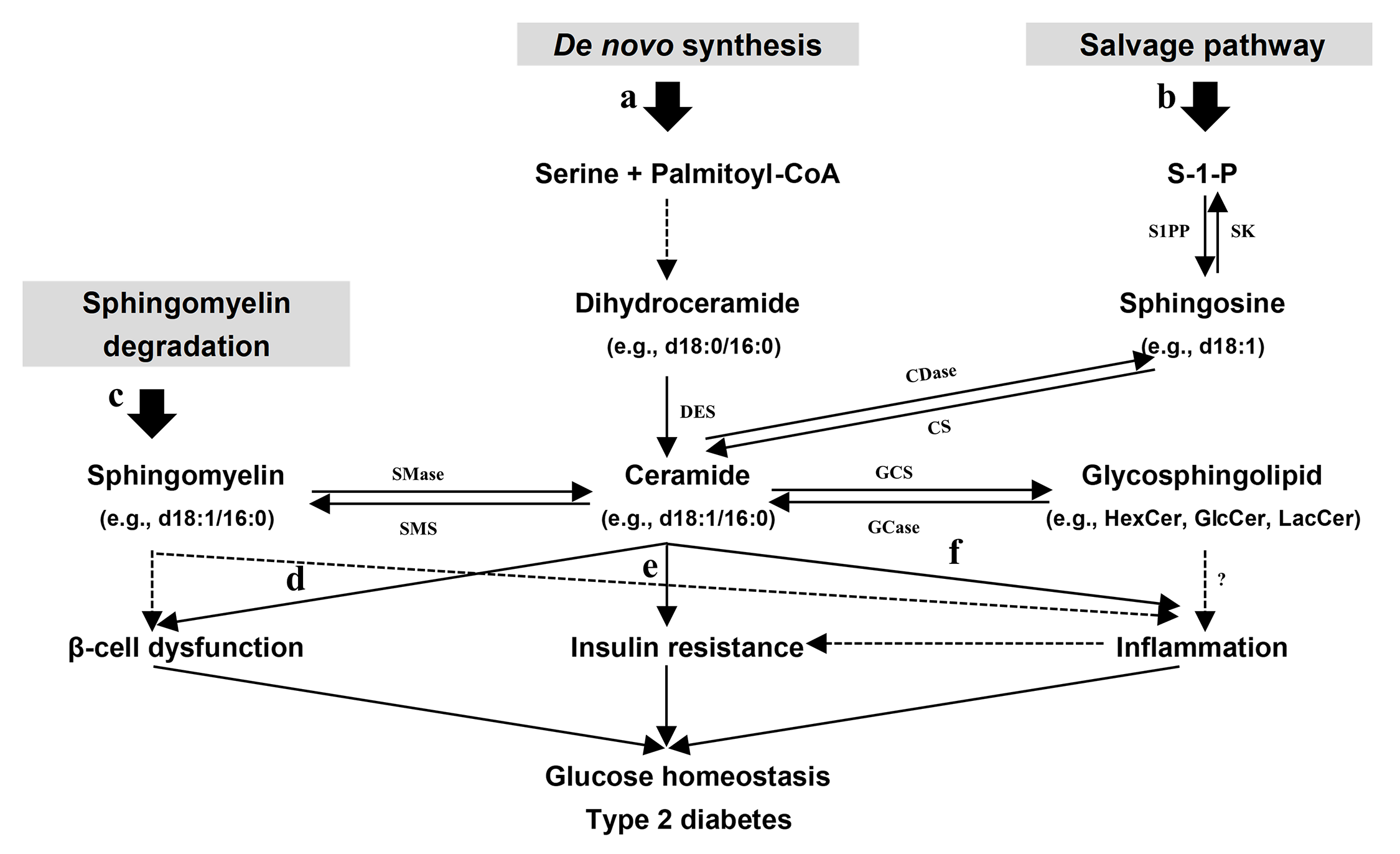

Supplement: S9 Fig — Ceramides can be produced by (a) a de novo pathway initiated from serine and palmitoyl precursors, (b) synthesis via sphingosine-1-phosphate (S-1-P), namely the “salvage pathway,” and (c) degradation of sphingomyelins through sphingomyelinase. As important signal molecules, ceramides can (d) induce pancreatic β-cell apoptosis through increasing endoplasmic reticulum stress, producing reactive oxygen species, (e) promote the development of insulin resistance via activating either protein phosphatase 2 or protein kinase C ζ, leading to attenuated serine/threonine protein kinase, and (f) activate NLR family 3 inflammasome and produce more cytokines. Solid lines represent a 1-step or certain process, whereas dotted lines represent multiple-step or uncertain processes. CDase, ceramidase; CS, ceramide synthase; DES, dihydroceramide synthase; GlcCer, glucosylceramide; GCase, glucosylceramidase; GCS, glucosylceramide synthase; HexCer, hexosylceramide; LacCer, lactosylceramide; S1PP, S-1-P phosphatase; SK, sphingosine kinase; SMase, sphingomyelinase; SMS, sphingomyelin synthase; T2D, type 2 diabetes. (TIF) [file pmed.1003451.s010.tif]
