# Supplementary material for: Associations among circulating sphingolipids, β-cell function, and risk of developing type 2 diabetes: A population-based cohort study in China
Source: PLoS Med. 2020 Dec 9;17(12):e1003451. doi: 10.1371/journal.pmed.1003451 (PMC7725305; doi:10.1371/journal.pmed.1003451)
Supplement: S1 Table — (DOCX) [file pmed.1003451.s011.docx]

**S1 Table. Tandem mass spectrometry parameters for analyzing human plasma sphingolipids^a^.**

| **Sphingolipids** | **Q1** | **Q3** | **Retention time**  **(min)** | **Concentration**  **(mg/L)** | **CV**  **(%)** | **Missing rate (%)** |
| --- | --- | --- | --- | --- | --- | --- |
| **Cers** | | | | | | |
| Cer(d18:1/14:0) (10^-2^) | 510.6 | 264.4 | 1.07 | 14.02 (12.73, 15.43) | 15 | 0 |
| Cer(d18:1/16:0) (10^-2^) | 538.6 | 264.4 | 1.07 | 48.21 (28.49, 81.59) | 17 | 0 |
| Cer(d18:1/18:0) (10^-2^) | 566.7 | 264.4 | 1.06 | 31.97 (18.54, 55.12) | 19 | 0 |
| Cer(d18:1/18:1) (10^-2^) | 564.8 | 264.4 | 1.10 | 5.06 (4.56, 5.62) | 24 | 0 |
| Cer(d18:1/20:0) (10^-2^) | 594.6 | 264.4 | 1.06 | 12.02 (9.84, 14.69) | 19 | 0 |
| Cer(d18:1/20:1) (10^-2^) | 592.6 | 264.4 | 1.06 | 7.06 (6.25, 7.97) | 26 | 0 |
| Cer(d18:1/22:0) | 622.7 | 264.4 | 1.05 | 0.95 (0.93, 0.96) | 17 | 0.04 |
| Cer(d18:1/22:1) | 620.7 | 264.4 | 1.05 | 0.55 (0.54, 0.55) | 24 | 0 |
| Cer(d18:1/24:0) | 650.8 | 264.4 | 1.04 | 2.80 (2.65, 2.95) | 16 | 0.40 |
| Cer(d18:1/24:1) | 648.8 | 264.4 | 1.05 | 3.03 (2.90, 3.15) | 17 | 0 |
| Cer(d18:1/26:0) | 678.9 | 264.4 | 1.05 | 0.58 (0.57, 0.59) | 25 | 0 |
| Cer(d18:1/26:1) (10^-2^) | 676.9 | 264.4 | 1.05 | 4.13 (3.76, 4.53) | 30 | 0 |
| **dhCers** | | | | | | |
| Cer(d18:0/16:0) (10^-2^) | 540.6 | 266.4 | 1.07 | 2.24 (2.16, 2.31) | 25 | 0 |
| Cer(d18:0/18:0) (10^-2^) | 568.7 | 266.4 | 1.07 | 1.59 (1.54, 1.65) | 27 | 0 |
| Cer(d18:0/18:1) (10^-2^) | 566.8 | 266.4 | 1.07 | 1.08 (1.05, 1.11) | 26 | 0 |
| Cer(d18:0/20:0) (10^-2^) | 596.7 | 266.4 | 1.07 | 0.89 (0.87, 0.91) | 28 | 0 |
| Cer(d18:0/20:1) (10^-2^) | 594.4 | 266.4 | 1.06 | 0.74 (0.73, 0.75) | 21 | 0 |
| Cer(d18:0/22:0) (10^-2^) | 624.8 | 266.4 | 1.05 | 4.88 (4.37, 5.45) | 25 | 0 |
| Cer(d18:0/22:1) (10^-2^) | 620.4 | 266.4 | 1.07 | 11.42 (8.81, 14.81) | 20 | 0 |
| Cer(d18:0/24:0) (10^-2^) | 652.9 | 266.4 | 1.05 | 12.07 (9.35, 15.60) | 22 | 0 |
| Cer(d18:0/24:1) (10^-2^) | 650.9 | 266.4 | 1.04 | 9.89 (8.38, 11.67) | 23 | 0 |
| **Saturated SMs** | | | | | | |
| SM C34:0 | 705.6 | 184.1 | 6.93 | 16.27 (13.64, 19.40) | 19 | 0.67 |
| SM C36:0 | 733.6 | 184.1 | 5.80 | 9.84 (8.19, 11.82) | 9 | 0 |
| SM C38:0 | 761.6 | 184.1 | 5.75 | 55.41 (34.47, 89.08) | 8 | 0 |
| SM C40:0 | 789.6 | 184.1 | 5.64 | 50.82 (39.42, 65.50) | 14 | 0 |
| SM C42:0 | 817.9 | 184.1 | 5.59 | 50.04 (41.70, 60.05) | 6 | 0 |
| **Unsaturated SMs** | | | | | | |
| SM C32:1 | 675.6 | 184.1 | 7.02 | 5.19 (4.78, 5.63) | 25 | 0 |
| SM C34:1 | 703.6 | 184.1 | 6.94 | 41.16 (29.21, 58.00) | 21 | 0 |
| SM C36:1 | 731.7 | 184.1 | 6.86 | 14.94 (12.85, 17.37) | 10 | 0 |
| SM C38:1 | 759.6 | 184.1 | 5.78 | 46.61 (30.37, 71.53) | 16 | 0 |
| SM C40:1 | 787.7 | 184.1 | 5.69 | 45.95 (29.90, 70.61) | 7 | 0 |
| SM C42:1 | 815.7 | 184.1 | 5.56 | 46.65 (37.46, 58.11) | 7 | 0 |
| SM C44:1 | 843.7 | 184.1 | 5.66 | 3.36 (3.20, 3.53) | 19 | 0 |
| SM C34:2 | 701.6 | 184.1 | 6.95 | 13.29 (11.61, 15.22) | 14 | 0 |

**S1 Table. Continued.**

| **Sphingolipids** | **Q1** | **Q3** | **Retention time (min)** | **Concentration**  **(mg/L)** | **CV**  **(%)** | **Missing rate (%)** |
| --- | --- | --- | --- | --- | --- | --- |
| SM C36:2 | 729.7 | 184.1 | 6.86 | 8.20 (7.40, 9.08) | 14 | 0 |
| SM C42:2 | 813.7 | 184.1 | 6.66 | 38.12 (28.19, 51.54) | 7 | 0 |
| SM C42:3 | 811.6 | 184.1 | 5.53 | 43.29 (34.94, 53.63) | 28 | 0 |
| SM C44:3 | 839.6 | 184.1 | 5.51 | 6.75 (6.17, 7.38) | 22 | 0 |
| **Hydroxyl-SM with one additional hydroxyl** | | | | | | |
| SM (OH) C32:2 | 689.6 | 184.1 | 6.98 | 3.54 (3.35, 3.74) | 16 | 0 |
| SM (OH) C34:0 | 721.6 | 184.1 | 5.85 | 2.91 (2.80, 3.01) | 0 | 0 |
| SM (OH) C34:1 | 719.5 | 184.1 | 7.08 | 1.41 (1.38, 1.44) | 24 | 0 |
| SM (OH) C34:2 | 717.6 | 184.1 | 6.90 | 1.88 (1.83, 1.93) | 17 | 0 |
| SM (OH) C36:1 | 747.6 | 184.1 | 5.78 | 7.09 (6.71, 7.49) | 10 | 0 |
| SM (OH) C36:2 | 745.7 | 184.1 | 5.79 | 8.86 (8.20, 9.56) | 8 | 0 |
| SM (OH) C36:3 | 743.5 | 184.1 | 5.81 | 9.45 (8.99, 9.94) | 19 | 0 |
| SM (OH) C38:1 | 775.6 | 184.1 | 5.70 | 4.35 (4.15, 4.57) | 11 | 0 |
| SM (OH) C38:2 | 773.6 | 184.1 | 5.71 | 11.69 (10.90, 12.53) | 6 | 0 |
| SM (OH) C38:3 | 771.5 | 184.1 | 5.73 | 24.57 (21.72, 27.78) | 19 | 0 |
| SM (OH) C40:1 | 803.7 | 184.1 | 6.67 | 3.57 (3.35, 3.81) | 14 | 0 |
| SM (OH) C40:2 | 801.7 | 184.1 | 6.67 | 12.6 (10.54, 15.07) | 23 | 0 |
| SM (OH) C40:3 | 799.7 | 184.1 | 5.78 | 8.49 (7.85, 9.19) | 14 | 0 |
| SM (OH) C40:4 | 797.6 | 184.1 | 5.59 | 16.62 (15.07, 18.33) | 6 | 0 |
| SM (OH) C42:2 | 829.7 | 184.1 | 5.76 | 2.43 (2.37, 2.48) | 5 | 0.04 |
| SM (OH) C42:3 | 827.7 | 184.1 | 5.76 | 6.18 (5.82, 6.57) | 13 | 0.04 |
| SM (OH) C42:4 | 825.8 | 184.1 | 6.65 | 0.58 (0.57, 0.58) | 14 | 0 |
| SM (OH) C44:0 | 861.6 | 184.1 | 5.45 | 1.08 (1.05, 1.10) | 16 | 0.04 |
| SM (OH) C44:1 | 859.7 | 184.1 | 5.46 | 1.21 (1.19, 1.23) | 22 | 0 |
| SM (OH) C44:3 | 855.6 | 184.1 | 5.57 | 1.85 (1.82, 1.88) | 19 | 0 |
| **Hydroxyl-SM with two additional hydroxyls** | | | | | | |
| SM (2OH) C30:2 | 677.6 | 184.1 | 7.02 | 1.34 (1.31, 1.38) | 20 | 0 |
| SM (2OH) C32:1 | 707.6 | 184.1 | 6.88 | 2.30 (2.23, 2.38) | 21 | 0 |
| SM (2OH) C34:1 | 735.6 | 184.1 | 5.82 | 14.55 (13.00, 16.29) | 19 | 0 |
| SM (2OH) C40:0 | 821.7 | 184.1 | 5.58 | 20.78 (19.92, 21.69) | 8 | 0 |
| SM (2OH) C40:1 | 819.7 | 184.1 | 5.59 | 45.55 (39.27, 52.83) | 21 | 0 |
| SM (2OH) C42:4 | 841.7 | 184.1 | 5.59 | 5.01 (4.72, 5.33) | 11 | 0.13 |
| **GSLs** | | | | | | |
| HexCer(d18:1/12:0) (10^-2^) | 644.5 | 264.4 | 1.06 | 3.67 (3.40, 3.96) | 27 | 0 |
| HexCer(d18:1/16:0) | 700.7 | 264.4 | 1.14 | 1.12 (1.10, 1.14) | 21 | 0 |
| HexCer(d18:1/18:0) | 728.8 | 264.4 | 1.16 | 0.62 (0.61, 0.62) | 25 | 0 |
| HexCer(d18:1/20:0) (10^-2^) | 756.7 | 264.4 | 1.14 | 14.95 (14.91, 14.99) | 22 | 0 |
| HexCer(d18:1/20:1) (10^-2^) | 754.7 | 264.4 | 1.15 | 10.24 (10.22, 10.26) | 23 | 0 |

**S1 Table. Continued.**

| **Sphingolipids** | **Q1** | **Q3** | **Retention time**  **(min)** | **Concentration**  **(mg/L)** | **CV**  **(%)** | **Missing rate (%)** |
| --- | --- | --- | --- | --- | --- | --- |
| HexCer(d18:1/22:0) | 784.9 | 264.4 | 1.14 | 1.22 (1.19, 1.25) | 30 | 0 |
| HexCer(d18:1/22:1) | 782.8 | 264.4 | 1.13 | 0.67 (0.66, 0.68) | 27 | 0 |
| HexCer(d18:1/24:0) | 812.9 | 264.4 | 1.13 | 1.61 (1.56, 1.66) | 28 | 0 |
| HexCer(d18:1/24:1) | 810.9 | 264.4 | 1.14 | 2.17 (2.09, 2.26) | 28 | 0 |
| GlcCer(d18:0/24:0) (10^-2^) | 814.9 | 266.4 | 1.14 | 6.34 (5.57, 7.23) | 26 | 0 |
| GlcCer(d18:0/24:1) (10^-2^) | 812.9 | 266.4 | 1.13 | 4.23 (3.88, 4.61) | 26 | 0 |
| LacCer(d18:1/20:1) (10^-2^) | 916.2 | 264.4 | 1.00 | 2.82 (2.70, 2.96) | 22 | 0.04 |

^a^Values are geometric means (95% confidential intervals). Q1, precursor ion m/z. Q3, second product ion m/z.

Abbreviations: CV, coefficients of variation; Cer, ceramide; dhCer, dihydroceramide; SM, sphingomyelin; SM (OH), hydroxyl-sphingomyelin (with one additional hydroxyl); SM (2OH), hydroxyl-sphingomyelin (with two additional hydroxyls); GSL, glycosphingolipid; HexCer, hexosylceramide; GlcCer, glucosylceramide; LacCer, lactosylceramide.
