# Supplementary material for: Associations among circulating sphingolipids, β-cell function, and risk of developing type 2 diabetes: A population-based cohort study in China
Source: PLoS Med. 2020 Dec 9;17(12):e1003451. doi: 10.1371/journal.pmed.1003451 (PMC7725305; doi:10.1371/journal.pmed.1003451)
Supplement: S2 Table — (DOCX) [file pmed.1003451.s012.docx]

**S2 Table.** **Baseline sphingolipids between incident T2D cases and non-cases^a^**.

| **Sphingolipids (mg/L)** | **Incident T2D** | | ***P* value** |
| --- | --- | --- | --- |
|  | **Non-cases (*n* = 1,445)** | **Cases (*n* = 529)** |  |
| **Cers** | | | |
| Cer(d18:1/14:0) (10^-2^) | 13.97 (12.52, 15.59) | 14.22 (11.89, 17.00) | 1.85×10^-1^ |
| Cer(d18:1/16:0) (10^-2^) | 47.67 (26.42, 86.02) | 49.57 (18.21, 134.91) | 1.67×10^-2^ |
| Cer(d18:1/18:0) (10^-2^) | 31.39 (17.27, 57.07) | 33.42 (11.22, 99.60) | 2.38×10^-2^ |
| Cer(d18:1/18:1) (10^-2^) | 4.91 (4.39, 5.51) | 5.38 (4.34, 6.68) | **3.18×10^-6^** |
| Cer(d18:1/20:0) (10^-2^) | 11.76 (9.52, 14.51) | 12.52 (8.13, 19.27) | **7.38×10^-6^** |
| Cer(d18:1/20:1) (10^-2^) | 6.85 (6.01, 7.8) | 7.51 (5.85, 9.66) | **1.07×10^-7^** |
| Cer(d18:1/22:0) | 0.92 (0.90, 0.94) | 1.03 (0.99, 1.06) | **5.83×10^-4^** |
| Cer(d18:1/22:1) | 0.53 (0.52, 0.53) | 0.61 (0.59, 0.62) | **3.99×10^-7^** |
| Cer(d18:1/24:0) | 2.73 (2.57, 2.9) | 3.10 (2.80, 3.42) | 8.24×10^-3^ |
| Cer(d18:1/24:1) | 2.98 (2.85, 3.11) | 3.16 (2.90, 3.44) | **1.77×10^-4^** |
| Cer(d18:1/26:0) | 0.57 (0.57, 0.58) | 0.58 (0.57, 0.59) | 3.84×10^-3^ |
| Cer(d18:1/26:1) (10^-2^) | 4.11 (3.68, 4.60) | 4.21 (3.65, 4.85) | 9.97×10^-1^ |
| **dhCers** | | | |
| Cer(d18:0/16:0) (10^-2^) | 2.20 (2.12, 2.28) | 2.34 (2.19, 2.49) | 1.61×10^-2^ |
| Cer(d18:0/18:0) (10^-2^) | 1.54 (1.49, 1.6) | 1.72 (1.61, 1.84) | **3.28×10^-5^** |
| Cer(d18:0/18:1) (10^-2^) | 1.05 (1.02, 1.09) | 1.13 (1.07, 1.20) | 5.26×10^-3^ |
| Cer(d18:0/20:0) (10^-2^) | 0.86 (0.84, 0.88) | 0.97 (0.93, 1.02) | **1.40×10^-5^** |
| Cer(d18:0/20:1) (10^-2^) | 0.72 (0.71, 0.73) | 0.78 (0.76, 0.80) | 8.88×10^-4^ |
| Cer(d18:0/22:0) (10^-2^) | 4.70 (4.17, 5.30) | 5.40 (4.33, 6.74) | **3.40×10^-5^** |
| Cer(d18:0/22:1) (10^-2^) | 11.10 (8.25, 14.94) | 12.52 (7.84, 19.99) | 4.69×10^-3^ |
| Cer(d18:0/24:0) (10^-2^) | 11.67 (8.78, 15.51) | 13.57 (8.31, 22.17) | **2.00×10^-4^** |
| Cer(d18:0/24:1) (10^-2^) | 9.67 (8.07, 11.59) | 10.42 (7.43, 14.63) | **3.07×10^-5^** |
| **Saturated SMs** | | | |
| SM C34:0 | 15.98 (13.15, 19.41) | 17.29 (12.31, 24.28) | **1.44×10^-4^** |
| SM C36:0 | 9.60 (7.80, 11.81) | 10.65 (7.50, 15.10) | **1.63×10^-7^** |
| SM C38:0 | 54.57 (32.22, 92.42) | 58.31 (23.37, 145.50) | **7.03×10^-9^** |
| SM C40:0 | 50.41 (37.85, 67.12) | 52.24 (32.66, 83.56) | **7.72×10^-7^** |
| SM C42:0 | 49.80 (40.56, 61.15) | 51.00 (36.13, 71.99) | 1.24×10^-3^ |
| **Unsaturated SMs** | | | |
| SM C32:1 | 5.04 (4.60, 5.52) | 5.61 (4.78, 6.58) | **2.60×10^-5^** |
| SM C34:1 | 40.52 (27.84, 58.97) | 43.43 (22.06, 85.47) | **3.25×10^-7^** |
| SM C36:1 | 14.63 (12.45, 17.20) | 15.73 (11.55, 21.42) | **3.59×10^-8^** |
| SM C38:1 | 46.06 (28.30, 74.97) | 48.38 (22.04, 106.18) | **4.04×10^-5^** |
| SM C40:1 | 45.29 (28.08, 73.04) | 48.1 (20.89, 110.78) | **5.88×10^-6^** |
| SM C42:1 | 46.42 (35.99, 59.86) | 47.36 (32.44, 69.14) | **3.08×10^-4^** |
| SM C44:1 | 3.32 (3.13, 3.51) | 3.53 (3.26, 3.82) | 1.05×10^-1^ |
| SM C34:2 | 13.03 (11.20, 15.15) | 14.18 (11.01, 18.28) | **1.92×10^-6^** |

**S2 Table. Continued.**

| **Sphingolipids (mg/L)** | **Incident T2D** | | ***P* value** |
| --- | --- | --- | --- |
|  | **Non-cases (*n* = 1,445)** | **Cases (*n* = 529)** |  |
| SM C36:2 | 8.01 (7.17, 8.95) | 8.66 (7.06, 10.62) | **1.20×10^-6^** |
| SM C42:2 | 37.78 (27.04, 52.78) | 39.05 (21.78, 70.00) | **6.50×10^-4^** |
| SM C42:3 | 42.93 (33.91, 54.34) | 44.53 (29.24, 67.80) | **1.27×10^-6^** |
| SM C44:3 | 6.65 (6.00, 7.37) | 7.16 (6.05, 8.48) | 3.42×10^-3^ |
| **Hydroxyl-SM with one additional hydroxyl** | | | |
| SM (OH) C32:2 | 3.47 (3.26, 3.69) | 3.75 (3.38, 4.18) | 4.66×10^-2^ |
| SM (OH) C34:0 | 2.86 (2.74, 2.98) | 3.05 (2.84, 3.27) | 1.46×10^-2^ |
| SM (OH) C34:1 | 1.38 (1.35, 1.42) | 1.49 (1.44, 1.55) | 8.40×10^-2^ |
| SM (OH) C34:2 | 1.86 (1.81, 1.92) | 1.93 (1.84, 2.04) | 6.39×10^-1^ |
| SM (OH) C36:1 | 7.09 (6.66, 7.55) | 7.07 (6.38, 7.84) | 3.02×10^-1^ |
| SM (OH) C36:2 | 8.82 (8.06, 9.66) | 8.94 (7.86, 10.17) | 6.84×10^-1^ |
| SM (OH) C36:3 | 9.38 (8.85, 9.94) | 9.76 (8.92, 10.68) | 8.36×10^-4^ |
| SM (OH) C38:1 | 4.37 (4.14, 4.62) | 4.26 (3.87, 4.70) | 1.65×10^-2^ |
| SM (OH) C38:2 | 11.68 (10.77, 12.67) | 11.70 (10.33, 13.25) | 6.42×10^-3^ |
| SM (OH) C38:3 | 24.34 (21.19, 27.95) | 25.44 (20.23, 32.01) | **2.14×10^-7^** |
| SM (OH) C40:1 | 3.45 (3.22, 3.71) | 3.94 (3.49, 4.46) | **3.22×10^-5^** |
| SM (OH) C40:2 | 12.3 (10.07, 15.03) | 13.52 (9.70, 18.85) | 1.32×10^-3^ |
| SM (OH) C40:3 | 8.40 (7.69, 9.18) | 8.84 (7.57, 10.33) | 3.00×10^-1^ |
| SM (OH) C40:4 | 16.50 (14.75, 18.45) | 17.13 (14.33, 20.49) | 9.01×10^-2^ |
| SM (OH) C42:2 | 2.41 (2.35, 2.47) | 2.48 (2.38, 2.58) | 8.08×10^-1^ |
| SM (OH) C42:3 | 6.14 (5.73, 6.58) | 6.40 (5.75, 7.12) | 3.49×10^-1^ |
| SM (OH) C42:4 | 0.57 (0.56, 0.57) | 0.61 (0.60, 0.62) | 1.55×10^-1^ |
| SM (OH) C44:0 | 1.08 (1.05, 1.12) | 1.04 (1.00, 1.08) | 4.68×10^-1^ |
| SM (OH) C44:1 | 1.21 (1.19, 1.23) | 1.23 (1.19, 1.26) | 9.97×10^-1^ |
| SM (OH) C44:3 | 1.85 (1.81, 1.88) | 1.85 (1.79, 1.90) | 3.95×10^-1^ |
| **Hydroxyl-SM with two additional hydroxyls** | | | |
| SM (2OH) C30:2 | 1.30 (1.26, 1.33) | 1.47 (1.40, 1.55) | **1.23×10^-5^** |
| SM (2OH) C32:1 | 2.25 (2.17, 2.33) | 2.48 (2.33, 2.64) | **6.20×10^-4^** |
| SM (2OH) C34:1 | 14.35 (12.67, 16.25) | 15.22 (12.21, 18.97) | **1.84×10^-8^** |
| SM (2OH) C40:0 | 20.75 (19.77, 21.78) | 20.89 (19.31, 22.60) | 2.17×10^-1^ |
| SM (2OH) C40:1 | 45.36 (38.38, 53.60) | 46.32 (35.00, 61.32) | **1.04×10^-4^** |
| SM (2OH) C42:4 | 4.98 (4.63, 5.35) | 5.19 (4.71, 5.72) | 1.10×10^-1^ |
| **GSLs** | | | |
| HexCer(d18:1/12:0) (10^-2^) | 3.59 (3.29, 3.92) | 3.86 (3.33, 4.46) | **3.32×10^-4^** |
| HexCer(d18:1/16:0) | 1.09 (1.07, 1.12) | 1.20 (1.15, 1.25) | 7.12×10^-3^ |
| HexCer(d18:1/18:0) | 0.60 (0.59, 0.61) | 0.67 (0.65, 0.69) | **4.41×10^-4^** |
| HexCer(d18:1/20:0) (10^-2^) | 0.15 (0.15, 0.15) | 0.15 (0.15, 0.15) | 7.83×10^-1^ |
| HexCer(d18:1/20:1) (10^-2^) | 0.10 (0.10, 0.10) | 0.11 (0.11, 0.11) | **1.32×10^-5^** |

**S2 Table. Continued.**

| **Sphingolipids (mg/L)** | **Incident T2D** | | ***P* value** |
| --- | --- | --- | --- |
|  | **Non-cases (*n* = 1,445)** | **Cases (*n* = 529)** |  |
| HexCer(d18:1/22:0) | 1.19 (1.16, 1.22) | 1.32 (1.27, 1.38) | 5.01×10^-2^ |
| HexCer(d18:1/22:1) | 0.66 (0.65, 0.67) | 0.71 (0.70, 0.73) | 2.34×10^-2^ |
| HexCer(d18:1/24:0) | 1.57 (1.52, 1.63) | 1.78 (1.68, 1.88) | 7.30×10^-3^ |
| HexCer(d18:1/24:1) | 2.14 (2.05, 2.24) | 2.29 (2.11, 2.47) | 9.93×10^-3^ |
| GlcCer(d18:0/24:0) (10^-2^) | 6.18 (5.35, 7.14) | 7.01 (5.43, 9.05) | 2.59×10^-3^ |
| GlcCer(d18:0/24:1) (10^-2^) | 4.20 (3.81, 4.63) | 4.30 (3.64, 5.07) | 1.53×10^-1^ |
| LacCer(d18:1/20:1) (10^-2^) | 2.76 (2.62, 2.91) | 3.03 (2.79, 3.30) | 3.87×10^-3^ |

^a^Values are geometric means (95% confidential intervals). *P* were calculated after adjustment for sex, age, region (Beijing or Shanghai), and residence (urban or rural). Only sphingolipids with Bonferroni-corrected *P* value < 0.05 are labelled bold.

Abbreviations: Cer, ceramide; dhCer, dihydroceramide; SM, sphingomyelin; SM (OH), hydroxyl-sphingomyelin (with one additional hydroxyl); SM (2OH), hydroxyl-sphingomyelin (with two additional hydroxyls); GSL, glycosphingolipid; HexCer, hexosylceramide; GlcCer, glucosylceramide; LacCer, lactosylceramide.
