# Supplementary material for: Associations among circulating sphingolipids, β-cell function, and risk of developing type 2 diabetes: A population-based cohort study in China
Source: PLoS Med. 2020 Dec 9;17(12):e1003451. doi: 10.1371/journal.pmed.1003451 (PMC7725305; doi:10.1371/journal.pmed.1003451)
Supplement: S3 Table — (DOCX) [file pmed.1003451.s013.docx]

**S3 Table.** **Individual sphingolipids and risk of incident T2D.**

| **Sphingolipids** | **Model 1^a^** | | | | **Model 2^b^** | | | | **Model 3^c^** | | | |
| --- | --- | --- | --- | --- | --- | --- | --- | --- | --- | --- | --- | --- |
|  | **RR (95% CI)** | ***P*** | ***P*_FDR** | ***P*_Bon** | **RR (95% CI)** | ***P*** | ***P*_FDR** | ***P*_Bon** | **RR (95% CI)** | ***P*** | ***P*_FDR** | ***P*_Bon** |
| **Cers** | | | | | | | | | | | | |
| Cer(d18:1/14:0) | 1.05 (0.98, 1.14) | 1.72×10^-1^ | 2.04×10^-1^ | 1 | 1.05 (0.97, 1.13) | 2.04×10^-1^ | 2.50×10^-1^ | 1 | 1.06 (0.99, 1.14) | 1.14×10^-1^ | 1.42×10^-1^ | 1 |
| Cer(d18:1/16:0) | 1.10 (1.02, 1.18) | 1.51×10^-2^ | **2.25×10^-2^** | 1 | 1.10 (1.02, 1.18) | 1.52×10^-2^ | **2.31×10^-2^** | 1 | 1.08 (1.01, 1.16) | 3.06×10^-2^ | 5.29×10^-2^ | 1 |
| Cer(d18:1/18:0) | 1.09 (1.01, 1.17) | 2.74×10^-2^ | **3.86×10^-2^** | 1 | 1.08 (1.01, 1.17) | 2.94×10^-2^ | **4.08×10^-2^** | 1 | 1.06 (0.99, 1.14) | 9.79×10^-2^ | 1.31×10^-1^ | 1 |
| Cer(d18:1/18:1) | 1.19 (1.11, 1.28) | 1.72×10^-6^ | **1.01×10^-5^** | **1.31×10^-4^** | 1.19 (1.11, 1.27) | 2.00×10^-6^ | **1.17×10^-5^** | **1.52×10^-4^** | 1.14 (1.06, 1.22) | 5.00×10^-4^ | **2.92×10^-3^** | **3.80×10^-2^** |
| Cer(d18:1/20:0) | 1.18 (1.10, 1.26) | 7.16×10^-6^ | **3.63×10^-5^** | **5.44×10^-4^** | 1.18 (1.10, 1.27) | 5.78×10^-6^ | **2.93×10^-5^** | **4.39×10^-4^** | 1.14 (1.06, 1.22) | 4.00×10^-4^ | **2.53×10^-3^** | **3.04×10^-2^** |
| Cer(d18:1/20:1) | 1.21 (1.13, 1.30) | 4.30×10^-8^ | **8.36×10^-7^** | **3.27×10^-6^** | 1.22 (1.13, 1.30) | 4.70×10^-8^ | **7.09×10^-7^** | **3.57×10^-6^** | 1.18 (1.10, 1.26) | 5.38×10^-6^ | **1.36×10^-4^** | **4.09×10^-4^** |
| Cer(d18:1/22:0) | 1.16 (1.07, 1.27) | 7.00×10^-4^ | **1.56×10^-3^** | 5.32×10^-2^ | 1.17 (1.07, 1.28) | 5.00×10^-4^ | **1.19×10^-3^** | **3.80×10^-2^** | 1.11 (1.02, 1.21) | 1.73×10^-2^ | **3.33×10^-2^** | 1 |
| Cer(d18:1/22:1) | 1.23 (1.13, 1.34) | 6.79×10^-7^ | **5.16×10^-6^** | **5.16×10^-5^** | 1.23 (1.14, 1.34) | 3.88×10^-7^ | **2.95×10^-6^** | **2.95×10^-5^** | 1.17 (1.08, 1.27) | 2.00×10^-4^ | **1.52×10^-3^** | **1.52×10^-2^** |
| Cer(d18:1/24:0) | 1.15 (1.04, 1.27) | 8.40×10^-3^ | **1.33×10^-2^** | 6.38×10^-1^ | 1.16 (1.05, 1.28) | 4.70×10^-3^ | **8.31×10^-3^** | 3.57×10^-1^ | 1.10 (1.00, 1.22) | 6.19×10^-2^ | 8.83×10^-2^ | 1 |
| Cer(d18:1/24:1) | 1.15 (1.06, 1.23) | 3.00×10^-4^ | **7.86×10^-4^** | **2.28×10^-2^** | 1.15 (1.07, 1.24) | 3.00×10^-4^ | **7.60×10^-4^** | **2.28×10^-2^** | 1.11 (1.03, 1.20) | 4.70×10^-3^ | **1.37×10^-2^** | 3.57×10^-1^ |
| Cer(d18:1/26:0) | 1.12 (1.04, 1.21) | 4.80×10^-3^ | **8.48×10^-3^** | 3.65×10^-1^ | 1.12 (1.03, 1.21) | 6.80×10^-3^ | **1.12×10^-2^** | 5.17×10^-1^ | 1.09 (1.01, 1.18) | 3.45×10^-2^ | 5.70×10^-2^ | 1 |
| Cer(d18:1/26:1) | 1.00 (0.93, 1.08) | 9.91×10^-1^ | 9.91×10^-1^ | 1 | 1.00 (0.93, 1.08) | 9.40×10^-1^ | 9.40×10^-1^ | 1 | 1.00 (0.93, 1.07) | 9.50×10^-1^ | 9.50×10^-1^ | 1 |
| **dhCers** | | | | | | | | | | | | |
| Cer(d18:0/16:0) | 1.10 (1.02, 1.19) | 1.73×10^-2^ | **2.53×10^-2^** | 1 | 1.09 (1.01, 1.18) | 2.30×10^-2^ | **3.30×10^-2^** | 1 | 1.07 (1.00, 1.16) | 6.37×10^-2^ | 8.83×10^-2^ | 1 |
| Cer(d18:0/18:0) | 1.17 (1.09, 1.27) | 3.79×10^-5^ | **1.50×10^-4^** | **2.88×10^-3^** | 1.17 (1.08, 1.26) | 1.00×10^-4^ | **2.81×10^-4^** | **7.60×10^-3^** | 1.13 (1.04, 1.22) | 2.50×10^-3^ | **7.92×10^-3^** | 1.90×10^-1^ |
| Cer(d18:0/18:1) | 1.11 (1.03, 1.19) | 4.50×10^-3^ | **8.14×10^-3^** | 3.42×10^-1^ | 1.10 (1.03, 1.18) | 5.70×10^-3^ | **9.63×10^-3^** | 4.33×10^-1^ | 1.10 (1.02, 1.18) | 9.60×10^-3^ | **2.15×10^-2^** | 7.30×10^-1^ |
| Cer(d18:0/20:0) | 1.18 (1.09, 1.28) | 3.95×10^-5^ | **1.50×10^-4^** | **3.00×10^-3^** | 1.18 (1.09, 1.28) | 4.57×10^-5^ | **1.70×10^-4^** | **3.47×10^-3^** | 1.14 (1.06, 1.24) | 1.00×10^-3^ | **4.00×10^-3^** | 7.60×10^-2^ |
| Cer(d18:0/20:1) | 1.14 (1.05, 1.23) | 1.00×10^-3^ | **2.05×10^-3^** | 7.60×10^-2^ | 1.13 (1.05, 1.22) | 1.40×10^-3^ | **2.80×10^-3^** | 1.06×10^-1^ | 1.09 (1.01, 1.17) | 2.06×10^-2^ | **3.73×10^-2^** | 1 |
| Cer(d18:0/22:0) | 1.20 (1.10, 1.31) | 1.00×10^-4^ | **3.04×10^-4^** | **7.60×10^-3^** | 1.20 (1.10, 1.32) | 1.00×10^-4^ | **2.81×10^-4^** | **7.60×10^-3^** | 1.13 (1.03, 1.24) | 7.90×10^-3^ | **1.94×10^-2^** | 6.00×10^-1^ |
| Cer(d18:0/22:1) | 1.13 (1.04, 1.23) | 3.20×10^-3^ | **5.93×10^-3^** | 2.43×10^-1^ | 1.13 (1.04, 1.23) | 4.10×10^-3^ | **7.42×10^-3^** | 3.12×10^-1^ | 1.08 (1.00, 1.18) | 6.12×10^-2^ | 8.83×10^-2^ | 1 |
| Cer(d18:0/24:0) | 1.21 (1.09, 1.34) | 3.00×10^-4^ | **7.86×10^-4^** | **2.28×10^-2^** | 1.21 (1.09, 1.34) | 2.00×10^-4^ | **5.43×10^-4^** | **1.52×10^-2^** | 1.14 (1.03, 1.26) | 1.26×10^-2^ | **2.59×10^-2^** | 9.58×10^-1^ |
| Cer(d18:0/24:1) | 1.16 (1.08, 1.25) | 4.33×10^-5^ | **1.57×10^-4^** | **3.29×10^-3^** | 1.16 (1.08, 1.25) | 1.00×10^-4^ | **2.81×10^-4^** | **7.60×10^-3^** | 1.10 (1.02, 1.19) | 1.02×10^-2^ | **2.15×10^-2^** | 7.75×10^-1^ |
| **Saturated SMs** | | | | | | | | | | | | |
| SM C34:0 | 1.18 (1.08, 1.29) | 2.00×10^-4^ | **5.63×10^-4^** | **1.52×10^-2^** | 1.19 (1.09, 1.29) | 1.00×10^-4^ | **2.81×10^-4^** | **7.60×10^-3^** | 1.16 (1.06, 1.26) | 6.00×10^-4^ | **3.26×10^-3^** | **4.56×10^-2^** |
| SM C36:0 | 1.22 (1.13, 1.32) | 1.51×10^-7^ | **1.91×10^-6^** | **1.15×10^-5^** | 1.23 (1.14, 1.33) | 5.60×10^-8^ | **7.09×10^-7^** | **4.26×10^-6^** | 1.17 (1.08, 1.26) | 1.00×10^-4^ | **8.44×10^-4^** | **7.60×10^-3^** |
| SM C38:0 | 1.22 (1.14, 1.31) | 2.30×10^-9^ | **1.75×10^-7^** | **1.75×10^-7^** | 1.23 (1.15, 1.31) | 6.30×10^-10^ | **4.79×10^-8^** | **4.79×10^-8^** | 1.18 (1.10, 1.26) | 2.00×10^-6^ | **7.60×10^-5^** | **1.52×10^-4^** |

**S3 Table. Continued.**

| **Sphingolipids** | **Model 1^a^** | | | | **Model 2^b^** | | | | **Model 3^c^** | | | |
| --- | --- | --- | --- | --- | --- | --- | --- | --- | --- | --- | --- | --- |
|  | **RR (95% CI)** | ***P*** | ***P*_FDR** | ***P*_Bon** | **RR (95% CI)** | ***P*** | ***P*_FDR** | ***P*_Bon** | **RR (95% CI)** | ***P*** | ***P*_FDR** | ***P*_Bon** |
| SM C40:0 | 1.19 (1.11, 1.27) | 4.10×10^-7^ | **3.90×10^-6^** | **3.12×10^-5^** | 1.19 (1.12, 1.27) | 1.14×10^-7^ | **1.22×10^-6^** | **8.66×10^-6^** | 1.14 (1.07, 1.22) | 1.00×10^-4^ | **8.44×10^-4^** | **7.60×10^-3^** |
| SM C42:0 | 1.14 (1.05, 1.23) | 1.00×10^-3^ | **2.05×10^-3^** | 7.60×10^-2^ | 1.15 (1.06, 1.24) | 6.00×10^-4^ | **1.34×10^-3^** | **4.56×10^-2^** | 1.09 (1.00, 1.18) | 4.66×10^-2^ | 6.94×10^-2^ | 1 |
| **Unsaturated SMs** | | | | | | | | | | | | |
| SM C32:1 | 1.21 (1.10, 1.32) | 3.38×10^-5^ | **1.43×10^-4^** | **2.57×10^-3^** | 1.20 (1.10, 1.32) | 5.19×10^-5^ | **1.79×10^-4^** | **3.94×10^-3^** | 1.12 (1.02, 1.22) | 1.95×10^-2^ | **3.61×10^-2^** | 1 |
| SM C34:1 | 1.23 (1.14, 1.33) | 2.47×10^-7^ | **2.68×10^-6^** | **1.88×10^-5^** | 1.23 (1.14, 1.33) | 1.28×10^-7^ | **1.22×10^-6^** | **9.73×10^-6^** | 1.18 (1.09, 1.27) | 2.84×10^-5^ | **5.40×10^-4^** | **2.16×10^-3^** |
| SM C36:1 | 1.24 (1.15, 1.34) | 4.40×10^-8^ | **8.36×10^-7^** | **3.34×10^-6^** | 1.24 (1.15, 1.34) | 4.50×10^-8^ | **7.09×10^-7^** | **3.42×10^-6^** | 1.17 (1.08, 1.26) | 1.00×10^-4^ | **8.44×10^-4^** | **7.60×10^-3^** |
| SM C38:1 | 1.14 (1.07, 1.22) | 4.82×10^-5^ | **1.62×10^-4^** | **3.66×10^-3^** | 1.15 (1.08, 1.22) | 3.08×10^-5^ | **1.30×10^-4^** | **2.34×10^-3^** | 1.11 (1.04, 1.19) | 1.60×10^-3^ | **5.79×10^-3^** | 1.22×10^-1^ |
| SM C40:1 | 1.16 (1.09, 1.24) | 5.73×10^-6^ | **3.11×10^-5^** | **4.35×10^-4^** | 1.17 (1.09, 1.25) | 2.88×10^-6^ | **1.56×10^-5^** | **2.19×10^-4^** | 1.12 (1.05, 1.20) | 8.00×10^-4^ | **3.80×10^-3^** | 6.08×10^-2^ |
| SM C42:1 | 1.13 (1.06, 1.21) | 2.00×10^-4^ | **5.63×10^-4^** | **1.52×10^-2^** | 1.14 (1.07, 1.22) | 1.00×10^-4^ | **2.81×10^-4^** | **7.60×10^-3^** | 1.09 (1.02, 1.16) | 1.69×10^-2^ | **3.33×10^-2^** | 1 |
| SM C44:1 | 1.10 (1.01, 1.21) | 3.83×10^-2^ | 5.20×10^-2^ | 1 | 1.10 (1.00, 1.20) | 4.45×10^-2^ | 6.04×10^-2^ | 1 | 1.10 (1.00, 1.21) | 3.97×10^-2^ | 6.03×10^-2^ | 1 |
| SM C34:2 | 1.26 (1.15, 1.38) | 1.58×10^-6^ | **1.00×10^-5^** | **1.20×10^-4^** | 1.26 (1.15, 1.39) | 7.43×10^-7^ | **5.13×10^-6^** | **5.65×10^-5^** | 1.17 (1.07, 1.28) | 9.00×10^-4^ | **3.80×10^-3^** | 6.84×10^-2^ |
| SM C36:2 | 1.22 (1.13, 1.32) | 1.20×10^-6^ | **8.29×10^-6^** | **9.12×10^-5^** | 1.22 (1.13, 1.33) | 1.12×10^-6^ | **7.09×10^-6^** | **8.51×10^-5^** | 1.15 (1.06, 1.24) | 9.00×10^-4^ | **3.80×10^-3^** | 6.84×10^-2^ |
| SM C42:2 | 1.14 (1.06, 1.24) | 9.00×10^-4^ | **1.95×10^-3^** | 6.84×10^-2^ | 1.15 (1.06, 1.24) | 7.00×10^-4^ | **1.48×10^-3^** | 5.32×10^-2^ | 1.12 (1.04, 1.21) | 3.40×10^-3^ | **1.03×10^-2^** | 2.58×10^-1^ |
| SM C42:3 | 1.20 (1.12, 1.29) | 6.34×10^-7^ | **5.16×10^-6^** | **4.82×10^-5^** | 1.20 (1.12, 1.29) | 2.48×10^-7^ | **2.09×10^-6^** | **1.88×10^-5^** | 1.14 (1.06, 1.23) | 4.00×10^-4^ | **2.53×10^-3^** | **3.04×10^-2^** |
| SM C44:3 | 1.13 (1.04, 1.22) | 2.50×10^-3^ | **4.87×10^-3^** | 1.90×10^-1^ | 1.14 (1.05, 1.23) | 1.40×10^-3^ | **2.80×10^-3^** | 1.06×10^-1^ | 1.09 (1.01, 1.18) | 3.31×10^-2^ | 5.59×10^-2^ | 1 |
| **Hydroxyl-SM with 1 additional hydroxyl** | | | | | | | | | | | | |
| SM (OH) C32:2 | 1.10 (1.00, 1.20) | 4.96×10^-2^ | 6.50×10^-2^ | 1 | 1.09 (1.00, 1.20) | 5.91×10^-2^ | 7.70×10^-2^ | 1 | 1.06 (0.97, 1.16) | 1.90×10^-1^ | 2.29×10^-1^ | 1 |
| SM (OH) C34:0 | 1.11 (1.02, 1.20) | 1.32×10^-2^ | **2.01×10^-2^** | 1 | 1.11 (1.02, 1.20) | 1.11×10^-2^ | **1.72×10^-2^** | 8.44×10^-1^ | 1.10 (1.02, 1.19) | 1.75×10^-2^ | **3.33×10^-2^** | 1 |
| SM (OH) C34:1 | 1.08 (0.99, 1.19) | 8.85×10^-2^ | 1.10×10^-1^ | 1 | 1.08 (0.99, 1.19) | 8.69×10^-2^ | 1.08×10^-1^ | 1 | 1.08 (0.98, 1.18) | 1.05×10^-1^ | 1.33×10^-1^ | 1 |
| SM (OH) C34:2 | 1.02 (0.94, 1.11) | 6.39×10^-1^ | 6.84×10^-1^ | 1 | 1.02 (0.94, 1.10) | 7.10×10^-1^ | 7.49×10^-1^ | 1 | 1.02 (0.94, 1.11) | 5.93×10^-1^ | 6.44×10^-1^ | 1 |
| SM (OH) C36:1 | 0.96 (0.89, 1.04) | 3.05×10^-1^ | 3.51×10^-1^ | 1 | 0.97 (0.90, 1.04) | 3.54×10^-1^ | 4.01×10^-1^ | 1 | 1.00 (0.92, 1.08) | 9.50×10^-1^ | 9.50×10^-1^ | 1 |
| SM (OH) C36:2 | 0.98 (0.91, 1.06) | 6.73×10^-1^ | 7.10×10^-1^ | 1 | 0.98 (0.91, 1.06) | 6.79×10^-1^ | 7.27×10^-1^ | 1 | 0.99 (0.91, 1.07) | 7.33×10^-1^ | 7.63×10^-1^ | 1 |
| SM (OH) C36:3 | 1.18 (1.07, 1.30) | 6.00×10^-4^ | **1.38×10^-3^** | **4.56×10^-2^** | 1.19 (1.08, 1.31) | 4.00×10^-4^ | **9.81×10^-4^** | **3.04×10^-2^** | 1.13 (1.03, 1.24) | 9.70×10^-3^ | **2.15×10^-2^** | 7.37×10^-1^ |
| SM (OH) C38:1 | 0.92 (0.85, 0.99) | 2.89×10^-2^ | **3.99×10^-2^** | 1 | 0.91 (0.85, 0.98) | 1.74×10^-2^ | **2.54×10^-2^** | 1 | 0.94 (0.87, 1.01) | 9.99×10^-2^ | 1.31×10^-1^ | 1 |
| SM (OH) C38:2 | 0.89 (0.82, 0.97) | 5.80×10^-3^ | **9.80×10^-3^** | 4.41×10^-1^ | 0.89 (0.82, 0.96) | 4.90×10^-3^ | **8.46×10^-3^** | 3.72×10^-1^ | 0.93 (0.85, 1.00) | 6.39×10^-2^ | 8.83×10^-2^ | 1 |
| SM (OH) C38:3 | 1.25 (1.15, 1.36) | 9.00×10^-8^ | **1.37×10^-6^** | **6.84×10^-6^** | 1.26 (1.16, 1.37) | 3.10×10^-8^ | **7.09×10^-7^** | **2.36×10^-6^** | 1.19 (1.09, 1.29) | 5.87×10^-5^ | **7.44×10^-4^** | **4.46×10^-3^** |
| SM (OH) C40:1 | 1.24 (1.12, 1.37) | 4.91×10^-5^ | **1.62×10^-4^** | **3.73×10^-3^** | 1.24 (1.12, 1.37) | 4.71×10^-5^ | **1.70×10^-4^** | **3.58×10^-3^** | 1.15 (1.04, 1.27) | 5.90×10^-3^ | **1.55×10^-2^** | 4.48×10^-1^ |

**S3 Table. Continued.**

| **Sphingolipids** | **Model 1^a^** | | | | **Model 2^b^** | | | | **Model 3^c^** | | | |
| --- | --- | --- | --- | --- | --- | --- | --- | --- | --- | --- | --- | --- |
|  | **RR (95% CI)** | ***P*** | ***P*_FDR** | ***P*_Bon** | **RR (95% CI)** | ***P*** | ***P*_FDR** | ***P*_Bon** | **RR (95% CI)** | ***P*** | ***P*_FDR** | ***P*_Bon** |
| SM (OH) C40:2 | 1.18 (1.06, 1.31) | 1.60×10^-3^ | **3.20×10^-3^** | 1.22×10^-1^ | 1.18 (1.07, 1.31) | 1.50×10^-3^ | **2.92×10^-3^** | 1.14×10^-1^ | 1.11 (1.01, 1.23) | 3.88×10^-2^ | 6.03×10^-2^ | 1 |
| SM (OH) C40:3 | 1.04 (0.96, 1.13) | 3.68×10^-1^ | 4.11×10^-1^ | 1 | 1.04 (0.96, 1.13) | 3.59×10^-1^ | 4.01×10^-1^ | 1 | 1.04 (0.96, 1.14) | 3.11×10^-1^ | 3.56×10^-1^ | 1 |
| SM (OH) C40:4 | 1.09 (0.99, 1.20) | 8.83×10^-2^ | 1.10×10^-1^ | 1 | 1.09 (0.99, 1.21) | 8.07×10^-2^ | 1.02×10^-1^ | 1 | 1.08 (0.98, 1.19) | 1.17×10^-1^ | 1.43×10^-1^ | 1 |
| SM (OH) C42:2 | 1.01 (0.90, 1.13) | 8.60×10^-1^ | 8.83×10^-1^ | 1 | 1.01 (0.91, 1.13) | 8.07×10^-1^ | 8.40×10^-1^ | 1 | 1.02 (0.92, 1.13) | 7.29×10^-1^ | 7.63×10^-1^ | 1 |
| SM (OH) C42:3 | 1.04 (0.96, 1.13) | 3.46×10^-1^ | 3.92×10^-1^ | 1 | 1.04 (0.96, 1.13) | 3.51×10^-1^ | 4.01×10^-1^ | 1 | 1.04 (0.96, 1.13) | 3.26×10^-1^ | 3.64×10^-1^ | 1 |
| SM (OH) C42:4 | 1.06 (0.98, 1.15) | 1.66×10^-1^ | 2.00×10^-1^ | 1 | 1.05 (0.97, 1.14) | 2.33×10^-1^ | 2.77×10^-1^ | 1 | 1.04 (0.97, 1.13) | 2.81×10^-1^ | 3.28×10^-1^ | 1 |
| SM (OH) C44:0 | 0.97 (0.89, 1.06) | 4.66×10^-1^ | 5.06×10^-1^ | 1 | 0.97 (0.89, 1.06) | 4.89×10^-1^ | 5.31×10^-1^ | 1 | 1.02 (0.93, 1.11) | 6.49×10^-1^ | 6.95×10^-1^ | 1 |
| SM (OH) C44:1 | 1.00 (0.93, 1.08) | 9.77×10^-1^ | 9.90×10^-1^ | 1 | 1.00 (0.93, 1.08) | 9.04×10^-1^ | 9.16×10^-1^ | 1 | 1.04 (0.96, 1.12) | 3.14×10^-1^ | 3.56×10^-1^ | 1 |
| SM (OH) C44:3 | 0.97 (0.89, 1.05) | 4.03×10^-1^ | 4.44×10^-1^ | 1 | 0.96 (0.89, 1.04) | 3.67×10^-1^ | 4.05×10^-1^ | 1 | 0.99 (0.92, 1.07) | 8.57×10^-1^ | 8.80×10^-1^ | 1 |
| **Hydroxyl-SM with 2 additional hydroxyls** | | | | | | | | | | | | |
| SM (2OH) C30:2 | 1.21 (1.11, 1.31) | 1.62×10^-5^ | **7.24×10^-5^** | **1.23×10^-3^** | 1.20 (1.10, 1.31) | 2.32×10^-5^ | **1.04×10^-4^** | **1.76×10^-3^** | 1.12 (1.03, 1.22) | 1.00×10^-2^ | **2.15×10^-2^** | 7.60×10^-1^ |
| SM (2OH) C32:1 | 1.18 (1.08, 1.29) | 4.00×10^-4^ | **9.50×10^-4^** | **3.04×10^-2^** | 1.18 (1.08, 1.30) | 3.00×10^-4^ | **7.60×10^-4^** | **2.28×10^-2^** | 1.16 (1.06, 1.26) | 8.00×10^-4^ | **3.80×10^-3^** | 6.08×10^-2^ |
| SM (2OH) C34:1 | 1.25 (1.16, 1.35) | 8.40×10^-9^ | **3.19×10^-7^** | **6.38×10^-7^** | 1.25 (1.16, 1.35) | 3.00×10^-9^ | **1.14×10^-7^** | **2.28×10^-7^** | 1.21 (1.12, 1.30) | 9.61×10^-7^ | **7.30×10^-5^** | **7.30×10^-5^** |
| SM (2OH) C40:0 | 1.05 (0.97, 1.15) | 2.21×10^-1^ | 2.58×10^-1^ | 1 | 1.05 (0.96, 1.14) | 2.61×10^-1^ | 3.05×10^-1^ | 1 | 1.04 (0.96, 1.13) | 3.72×10^-1^ | 4.10×10^-1^ | 1 |
| SM (2OH) C40:1 | 1.15 (1.07, 1.23) | 1.00×10^-4^ | **3.04×10^-4^** | **7.60×10^-3^** | 1.16 (1.08, 1.24) | 3.59×10^-5^ | **1.44×10^-4^** | **2.73×10^-3^** | 1.10 (1.02, 1.19) | 8.80×10^-3^ | **2.09×10^-2^** | 6.69×10^-1^ |
| SM (2OH) C42:4 | 1.07 (0.99, 1.15) | 7.55×10^-2^ | 9.73×10^-2^ | 1 | 1.07 (1.00, 1.16) | 5.98×10^-2^ | 7.70×10^-2^ | 1 | 1.07 (0.99, 1.15) | 1.03×10^-1^ | 1.32×10^-1^ | 1 |
| **GSLs** | | | | | | | | | | | | |
| HexCer(d18:1/12:0) | 1.15 (1.06, 1.24) | 4.00×10^-4^ | **9.50×10^-4^** | **3.04×10^-2^** | 1.14 (1.06, 1.23) | 7.00×10^-4^ | **1.48×10^-3^** | 5.32×10^-2^ | 1.13 (1.04, 1.21) | 2.40×10^-3^ | **7.92×10^-3^** | 1.82×10^-1^ |
| HexCer(d18:1/16:0) | 1.12 (1.03, 1.21) | 6.80×10^-3^ | **1.10×10^-2^** | 5.17×10^-1^ | 1.12 (1.03, 1.21) | 8.20×10^-3^ | **1.30×10^-2^** | 6.23×10^-1^ | 1.11 (1.03, 1.20) | 6.90×10^-3^ | **1.75×10^-2^** | 5.24×10^-1^ |
| HexCer(d18:1/18:0) | 1.15 (1.07, 1.25) | 4.00×10^-4^ | **9.50×10^-4^** | **3.04×10^-2^** | 1.15 (1.06, 1.25) | 6.00×10^-4^ | **1.34×10^-3^** | **4.56×10^-2^** | 1.13 (1.05, 1.22) | 1.60×10^-3^ | **5.79×10^-3^** | 1.22×10^-1^ |
| HexCer(d18:1/20:0) | 1.01 (0.94, 1.09) | 7.71×10^-1^ | 8.02×10^-1^ | 1 | 1.01 (0.94, 1.08) | 8.41×10^-1^ | 8.64×10^-1^ | 1 | 1.04 (0.97, 1.12) | 2.76×10^-1^ | 3.28×10^-1^ | 1 |
| HexCer(d18:1/20:1) | 1.18 (1.10, 1.27) | 1.31×10^-5^ | **6.22×10^-5^** | **9.96×10^-4^** | 1.18 (1.09, 1.27) | 2.28×10^-5^ | **1.04×10^-4^** | **1.73×10^-3^** | 1.17 (1.08, 1.26) | 3.67×10^-5^ | **5.58×10^-4^** | **2.79×10^-3^** |
| HexCer(d18:1/22:0) | 1.09 (1.00, 1.19) | 4.62×10^-2^ | 6.16×10^-2^ | 1 | 1.09 (1.00, 1.19) | 5.05×10^-2^ | 6.73×10^-2^ | 1 | 1.09 (1.01, 1.19) | 3.53×10^-2^ | 5.71×10^-2^ | 1 |
| HexCer(d18:1/22:1) | 1.09 (1.01, 1.17) | 2.40×10^-2^ | **3.44×10^-2^** | 1 | 1.09 (1.01, 1.17) | 2.95×10^-2^ | **4.08×10^-2^** | 1 | 1.08 (1.00, 1.16) | 3.89×10^-2^ | 6.03×10^-2^ | 1 |
| HexCer(d18:1/24:0) | 1.14 (1.04, 1.24) | 6.50×10^-3^ | **1.07×10^-2^** | 4.94×10^-1^ | 1.13 (1.03, 1.24) | 7.40×10^-3^ | **1.20×10^-2^** | 5.62×10^-1^ | 1.13 (1.04, 1.24) | 5.40×10^-3^ | **1.52×10^-2^** | 4.10×10^-1^ |
| HexCer(d18:1/24:1) | 1.11 (1.02, 1.20) | 1.20×10^-2^ | **1.86×10^-2^** | 9.12×10^-1^ | 1.10 (1.02, 1.20) | 1.64×10^-2^ | **2.44×10^-2^** | 1 | 1.12 (1.03, 1.21) | 5.70×10^-3^ | **1.55×10^-2^** | 4.33×10^-1^ |
| GlcCer(d18:0/24:0) | 1.14 (1.05, 1.25) | 2.70×10^-3^ | **5.13×10^-3^** | 2.05×10^-1^ | 1.14 (1.05, 1.25) | 3.00×10^-3^ | **5.70×10^-3^** | 2.28×10^-1^ | 1.14 (1.05, 1.24) | 2.50×10^-3^ | **7.92×10^-3^** | 1.90×10^-1^ |

**S3 Table. Continued.**

| **Sphingolipids** | **Model 1^a^** | | | | **Model 2^b^** | | | | **Model 3^c^** | | | |
| --- | --- | --- | --- | --- | --- | --- | --- | --- | --- | --- | --- | --- |
|  | **RR (95% CI)** | ***P*** | ***P*_FDR** | ***P*_Bon** | **RR (95% CI)** | ***P*** | ***P*_FDR** | ***P*_Bon** | **RR (95% CI)** | ***P*** | ***P*_FDR** | ***P*_Bon** |
| GlcCer(d18:0/24:1) | 1.06 (0.98, 1.14) | 1.60×10^-1^ | 1.96×10^-1^ | 1 | 1.05 (0.97, 1.13) | 2.25×10^-1^ | 2.71×10^-1^ | 1 | 1.07 (0.99, 1.16) | 7.46×10^-2^ | 1.01×10^-1^ | 1 |
| LacCer(d18:1/20:1) | 1.13 (1.04, 1.24) | 5.20×10^-3^ | **8.98×10^-3^** | 3.95×10^-1^ | 1.14 (1.04, 1.25) | 3.50×10^-3^ | **6.49×10^-3^** | 2.66×10^-1^ | 1.11 (1.01, 1.21) | 2.36×10^-2^ | **4.17×10^-2^** | 1 |

Data are RRs (95% CIs) and *P* values calculated using log-Poisson regression. Significance are labelled bold.

^a^Model 1: Age, sex, region (Beijing or Shanghai), and residence (urban or rural).

^b^Model 2: Model 1 plus educational attainment (0-6 years, 7-9 years, ≥10 years), current smoking (yes or no), current alcohol drinking (yes or no), physical activity (low, moderate, or high), and family history of diabetes (yes or no).

^c^Model 3: Model 2 plus BMI.

Cer, ceramide; Bon, Bonferroni; dhCer, dihydroceramide; FDR, false discovery rate; GSL, glycosphingolipid; GlcCer, glucosylceramide; HexCer, hexosylceramide; LacCer, lactosylceramide; RR, relative risk; SM, sphingomyelin; SM (OH), hydroxyl-sphingomyelin with 1 additional hydroxyl; SM (2OH), hydroxyl-sphingomyelin with 2 additional hydroxyls; T2D, type 2 diabetes.
