# Supplementary material for: Associations among circulating sphingolipids, β-cell function, and risk of developing type 2 diabetes: A population-based cohort study in China
Source: PLoS Med. 2020 Dec 9;17(12):e1003451. doi: 10.1371/journal.pmed.1003451 (PMC7725305; doi:10.1371/journal.pmed.1003451)
Supplement: S4 Table — (DOCX) [file pmed.1003451.s014.docx]

**S4 Table. Conditional analysis of plasma sphingolipids with incident T2D.**

| **Sphingolipids** | **Model 1^a^** | | **Model 2^b^** | | **Model 3^c^** | | **Model 4^d^** | |
| --- | --- | --- | --- | --- | --- | --- | --- | --- |
|  | **RR (95% CI)** | ***P*** | **RR (95% CI)** | ***P*** | **RR (95% CI)** | ***P*** | **RR (95% CI)** | ***P*** |
| Cer(d18:1/18:1) | 1.13 (1.05, 1.22) | 9.00×10^-4^ | 1.14 (1.06, 1.22) | **5.00×10^-4^** | 1.13 (1.05, 1.21) | 9.00×10^-4^ | 1.13 (1.05, 1.21) | 8.00×10^-4^ |
| Cer(d18:1/20:0) | 1.14 (1.06, 1.23) | 7.00×10^-4^ | 1.14 (1.06, 1.23) | **4.00×10^-4^** | 1.13 (1.05, 1.22) | 8.00×10^-4^ | 1.14 (1.06, 1.22) | **4.00×10^-4^** |
| Cer(d18:1/20:1) | 1.18 (1.10, 1.27) | **9.55×10^-6^** | 1.18 (1.10, 1.26) | **4.64×10^-6^** | 1.17 (1.09, 1.26) | **1.11×10^-5^** | 1.17 (1.09, 1.25) | **1.20×10^-5^** |
| Cer(d18:1/22:1) | 1.18 (1.08, 1.29) | **2.00×10^-4^** | 1.17 (1.08, 1.27) | **1.00×10^-4^** | 1.16 (1.07, 1.27) | **3.00×10^-4^** | 1.16 (1.07, 1.26) | **3.00×10^-4^** |
| SM C34:0 | 1.17 (1.07, 1.28) | 9.00×10^-4^ | 1.18 (1.08, 1.29) | **2.00×10^-4^** | 1.16 (1.07, 1.26) | **5.00×10^-4^** | 1.15 (1.06, 1.25) | **9.00×10^-4^** |
| SM C36:0 | 1.16 (1.08, 1.25) | **1.00×10^-4^** | 1.17 (1.08, 1.26) | **1.00×10^-4^** | 1.17 (1.08, 1.27) | **2.00×10^-4^** | 1.16 (1.07, 1.25) | **2.00×10^-4^** |
| SM C38:0 | 1.17 (1.10, 1.26) | **3.51×10^-6^** | 1.18 (1.10, 1.26) | **1.38×10^-6^** | 1.18 (1.10, 1.26) | **4.92×10^-6^** | 1.17 (1.09, 1.25) | **5.20×10^-6^** |
| SM C40:0 | 1.14 (1.06, 1.22) | **2.00×10^-4^** | 1.14 (1.07, 1.22) | **2.00×10^-4^** | 1.14 (1.06, 1.22) | **4.00×10^-4^** | 1.13 (1.06, 1.21) | **4.00×10^-4^** |
| SM C34:1 | 1.18 (1.09, 1.28) | **5.01×10^-5^** | 1.19 (1.10, 1.29) | **1.28×10^-5^** | 1.18 (1.09, 1.27) | **3.54×10^-5^** | 1.17 (1.08, 1.26) | **5.48×10^-5^** |
| SM C36:1 | 1.19 (1.09, 1.29) | **1.00×10^-4^** | 1.18 (1.09, 1.28) | **4.01×10^-5^** | 1.17 (1.08, 1.26) | **1.00×10^-4^** | 1.17 (1.08, 1.26) | **1.00×10^-4^** |
| SM C42:3 | 1.14 (1.06, 1.22) | 7.00×10^-4^ | 1.14 (1.06, 1.23) | **4.00×10^-4^** | 1.21 (1.12, 1.31) | **2.34×10^-6^** | 1.13 (1.05, 1.21) | 1.20×10^-3^ |
| SM (2OH) C34:1 | 1.20 (1.12, 1.30) | **1.53×10^-6^** | 1.21 (1.12, 1.31) | **4.89×10^-7^** | 1.19 (1.09, 1.29) | **1.00×10^-4^** | 1.19 (1.11, 1.29) | **3.49×10^-6^** |
| SM (OH) C38:3 | 1.19 (1.09, 1.29) | **1.00×10^-4^** | 1.19 (1.10, 1.30) | **4.69×10^-5^** | 1.14 (1.05, 1.23) | 1.00×10^-3^ | 1.18 (1.09, 1.28) | **1.00×10^-4^** |
| HexCer(d18:1/20:1) | 1.17 (1.08, 1.27) | **5.80×10^-5^** | 1.18 (1.09, 1.27) | **1.68×10^-5^** | 1.17 (1.09, 1.26) | **2.95×10^-5^** | 1.16 (1.08, 1.25) | **5.73×10^-5^** |
| Module yellow | 1.15 (1.07, 1.23) | **1.00×10^-4^** | 1.15 (1.07, 1.23) | **1.00×10^-4^** | 1.14 (1.07, 1.23) | **2.00×10^-4^** | 1.14 (1.06, 1.22) | **3.00×10^-4^** |
| Module turquoise | 1.20 (1.10, 1.31) | **4.18×10^-5^** | 1.19 (1.10, 1.29) | **1.73×10^-5^** | 1.17 (1.08, 1.27) | **1.00×10^-4^** | 1.17 (1.08, 1.27) | **1.00×10^-4^** |
| Module green | 1.17 (1.06, 1.29) | **2.20×10^-3^** | 1.16 (1.06, 1.28) | **1.30×10^-3^** | 1.15 (1.05, 1.27) | **2.70×10^-3^** | 1.15 (1.05, 1.26) | **2.50×10^-3^** |
| Module brown | 1.13 (1.04, 1.22) | **3.10×10^-3^** | 1.14 (1.05, 1.23) | **1.00×10^-3^** | 1.13 (1.05, 1.22) | **1.20×10^-3^** | 1.12 (1.04, 1.21) | **3.00×10^-3^** |

**S4 Table. Continued.**

| **Sphingolipids** | **Model 5^e^** | | **Model 6^f^** | | **Model 7^g^** | | **Model 8^h^** | |
| --- | --- | --- | --- | --- | --- | --- | --- | --- |
|  | **RR (95% CI)** | ***P*** | **RR (95% CI)** | ***P*** | **RR (95% CI)** | ***P*** | **RR (95% CI)** | ***P*** |
| Cer(d18:1/18:1) | 1.03 (0.95, 1.10) | 4.81×10^-1^ | 1.13 (1.05, 1.21) | 1.10×10^-3^ | 1.12 (1.04, 1.20) | 2.80×10^-3^ | 1.13 (1.05, 1.22) | 1.20×10^-3^ |
| Cer(d18:1/20:0) | 1.03 (0.96, 1.10) | 4.22×10^-1^ | 1.13 (1.05, 1.22) | 8.00×10^-4^ | 1.11 (1.03, 1.19) | 3.70×10^-3^ | 1.13 (1.05, 1.22) | 1.20×10^-3^ |
| Cer(d18:1/20:1) | 1.03 (0.96, 1.10) | 4.62×10^-1^ | 1.17 (1.09, 1.25) | **1.57×10^-5^** | 1.14 (1.06, 1.22) | **3.00×10^-4^** | 1.17 (1.09, 1.26) | **1.29×10^-5^** |
| Cer(d18:1/22:1) | 0.96 (0.88, 1.03) | 2.66×10^-1^ | 1.16 (1.07, 1.26) | **5.00×10^-4^** | 1.13 (1.04, 1.22) | 4.40×10^-3^ | 1.17 (1.08, 1.27) | **2.00×10^-4^** |
| SM C34:0 | 0.91 (0.84, 0.98) | 1.46×10^-2^ | 1.15 (1.06, 1.25) | 9.00×10^-4^ | 1.10 (1.01, 1.20) | 2.89×10^-2^ | 1.18 (1.08, 1.28) | **1.00×10^-4^** |
| SM C36:0 | 1.11 (1.03, 1.19) | 6.90×10^-3^ | 1.15 (1.07, 1.25) | **2.00×10^-4^** | 1.17 (1.09, 1.27) | **3.30×10^-5^** | 1.16 (1.07, 1.25) | **2.00×10^-4^** |
| SM C38:0 | 0.96 (0.89, 1.03) | 2.91×10^-1^ | 1.16 (1.09, 1.25) | **1.20×10^-5^** | 1.14 (1.07, 1.22) | **1.00×10^-4^** | 1.17 (1.09, 1.25) | **1.01×10^-5^** |
| SM C40:0 | 0.96 (0.89, 1.03) | 2.45×10^-1^ | 1.12 (1.05, 1.20) | 1.00×10^-3^ | 1.12 (1.04, 1.20) | 1.40×10^-3^ | 1.13 (1.05, 1.21) | 9.00×10^-4^ |
| SM C34:1 | 0.90 (0.83, 0.97) | 6.90×10^-3^ | 1.17 (1.08, 1.26) | **1.00×10^-4^** | 1.13 (1.04, 1.22) | 3.90×10^-3^ | 1.18 (1.10, 1.28) | **2.00×10^-5^** |
| SM C36:1 | 1.01 (0.94, 1.08) | 8.62×10^-1^ | 1.16 (1.08, 1.26) | **1.00×10^-4^** | 1.13 (1.04, 1.22) | 2.30×10^-3^ | 1.17 (1.08, 1.27) | **1.00×10^-4^** |
| SM C42:3 | 0.98 (0.91, 1.06) | 5.95×10^-1^ | 1.13 (1.04, 1.21) | 1.90×10^-3^ | 1.12 (1.04, 1.20) | 3.70×10^-3^ | 1.13 (1.05, 1.22) | 1.80×10^-3^ |
| SM (2OH) C34:1 | 1.08 (1.01, 1.16) | 3.21×10^-2^ | 1.19 (1.11, 1.29) | **2.81×10^-6^** | 1.19 (1.10, 1.28) | **4.64×10^-6^** | 1.21 (1.13, 1.31) | **4.40×10^-7^** |
| SM (OH) C38:3 | 0.95 (0.87, 1.03) | 2.28×10^-1^ | 1.18 (1.08, 1.28) | **2.00×10^-4^** | 1.15 (1.05, 1.25) | 1.70×10^-3^ | 1.18 (1.08, 1.29) | **2.00×10^-4^** |
| HexCer(d18:1/20:1) | 0.96 (0.90, 1.04) | 3.37×10^-1^ | 1.16 (1.08, 1.25) | **1.00×10^-4^** | 1.12 (1.04, 1.21) | 3.20×10^-3^ | 1.18 (1.09, 1.27) | **1.68×10^-5^** |
| Module yellow | 1.03 (0.95, 1.10) | 4.81×10^-1^ | 1.13 (1.05, 1.22) | **6.00×10^-4^** | 1.12 (1.04, 1.20) | **2.00×10^-3^** | 1.14 (1.06, 1.22) | **3.00×10^-4^** |
| Module turquoise | 1.03 (0.96, 1.10) | 4.22×10^-1^ | 1.17 (1.08, 1.26) | **1.00×10^-4^** | 1.13 (1.04, 1.22) | **2.40×10^-3^** | 1.17 (1.08, 1.26) | **1.00×10^-4^** |
| Module green | 1.03 (0.96, 1.10) | 4.62×10^-1^ | 1.14 (1.04, 1.25) | **4.30×10^-3^** | 1.13 (1.03, 1.23) | **1.10×10^-2^** | 1.15 (1.05, 1.26) | **3.50×10^-3^** |
| Module brown | 0.96 (0.88, 1.03) | 2.66×10^-1^ | 1.12 (1.04, 1.21) | **2.80×10^-3^** | 1.08 (1.00, 1.17) | **4.30×10^-2^** | 1.13 (1.05, 1.22) | **1.80×10^-3^** |

Conventional factors: age, sex, region (Beijing or Shanghai), residence (urban or rural), educational attainment (0-6 years, 7-9 years, or ≥10 years), current smoking (yes or no), current alcohol drinking (yes or no), physical activity (low, moderate, or high), family history of diabetes (yes or no), and BMI. Significance are labelled bold.

^a^Model 1: conventional factors plus LDL-cholesterol.

^b^Model 2: conventional factors plus HDL-cholesterol.

^c^Model 3: conventional factors plus triglycerides.

^d^Model 4: conventional factors plus systolic blood pressure and diastolic blood pressure.

^e^Model 5: conventional factors plus fasting glucose.

^f^Model 6: conventional factors plus HOMA-IR.

^g^Model 7: conventional factors plus HOMA-B.

^h^Model 8: conventional factors plus hsCRP and adiponectin.

Cer, ceramide; HOMA-IR, homeostatic model assessment of insulin resistance; HOMA-B, homeostatic model assessment of β-cell function; hsCRP, high-sensitivity C-reactive protein; HexCer, hexosylceramide; RR, relative risk; SM, sphingomyelin; SM (OH), hydroxyl-sphingomyelin with 1 additional hydroxyl; SM (2OH), hydroxyl-sphingomyelin with 2 additional hydroxyls; T2D, type 2 diabetes.
