# Supplementary material for: Associations among circulating sphingolipids, β-cell function, and risk of developing type 2 diabetes: A population-based cohort study in China
Source: PLoS Med. 2020 Dec 9;17(12):e1003451. doi: 10.1371/journal.pmed.1003451 (PMC7725305; doi:10.1371/journal.pmed.1003451)
Supplement: S5 Table — (DOCX) [file pmed.1003451.s015.docx]

**S5 Table. Stratified analyses of the associations between plasma sphingolipids and incident T2D.**

|  | **N** | **Cer(d18:1/18:1)** | ***P*** | ***P-_inter_*** | **Cer(d18:1/20:0)** | ***P*** | ***P-_inter_*** | **Cer(d18:1/20:1)** | ***P*** | ***P-_inter_*** | **Cer(d18:1/22:1)** | ***P*** | ***P-_inter_*** |
| --- | --- | --- | --- | --- | --- | --- | --- | --- | --- | --- | --- | --- | --- |
| Age, y |  |  |  |  |  |  |  |  |  |  |  |  |  |
| 50-59 | 1176 | 1.04 (0.94, 1.14) | 0.464 | **0.011** | 1.07 (0.97, 1.18) | 0.207 | 0.042 | 1.15 (1.05, 1.27) | 0.004 | 0.493 | 1.11 (0.99, 1.24) | 0.067 | 0.430 |
| 60-70 | 798 | 1.26 (1.13, 1.40) | <0.001 |  | 1.23 (1.11, 1.36) | <0.001 |  | 1.19 (1.07, 1.32) | 0.001 |  | 1.25 (1.10, 1.41) | <0.001 |  |
| Sex |  |  |  |  |  |  |  |  |  |  |  |  |  |
| Men | 826 | 1.15 (1.03, 1.29) | 0.011 | 0.687 | 1.15 (1.04, 1.28) | 0.01 | 0.986 | 1.24 (1.12, 1.37) | <0.001 | 0.241 | 1.13 (0.99, 1.28) | 0.071 | 0.743 |
| Women | 1148 | 1.12 (1.02, 1.23) | 0.023 |  | 1.12 (1.02, 1.24) | 0.019 |  | 1.12 (1.02, 1.24) | 0.02 |  | 1.19 (1.07, 1.33) | 0.001 |  |
| Region |  |  |  |  |  |  |  |  |  |  |  |  |  |
| Beijing | 935 | 1.08 (0.99, 1.18) | 0.101 | 0.060 | 1.07 (0.97, 1.17) | 0.183 | **0.007** | 1.13 (1.03, 1.23) | 0.007 | 0.128 | 1.10 (1.01, 1.21) | 0.035 | 0.123 |
| Shanghai | 1039 | 1.20 (1.07, 1.34) | 0.002 |  | 1.26 (1.12, 1.41) | <0.001 |  | 1.23 (1.10, 1.38) | <0.001 |  | 1.24 (1.08, 1.41) | 0.002 |  |
| Residence |  |  |  |  |  |  |  |  |  |  |  |  |  |
| Rural | 1154 | 1.10 (1.00, 1.21) | 0.062 | 0.109 | 1.12 (1.02, 1.24) | 0.016 | 0.537 | 1.15 (1.05, 1.26) | 0.003 | 0.303 | 1.22 (1.10, 1.36) | <0.001 | 0.749 |
| Urban | 820 | 1.19 (1.07, 1.32) | 0.001 |  | 1.16 (1.04, 1.29) | 0.009 |  | 1.20 (1.08, 1.33) | 0.001 |  | 1.11 (0.99, 1.25) | 0.077 |  |
| Smoking |  |  |  |  |  |  |  |  |  |  |  |  |  |
| Yes | 549 | 1.05 (0.92, 1.21) | 0.435 | 0.337 | 1.08 (0.94, 1.25) | 0.27 | 0.439 | 1.17 (1.03, 1.33) | 0.018 | 0.925 | 1.06 (0.91, 1.24) | 0.464 | 0.521 |
| No | 1425 | 1.16 (1.07, 1.26) | <0.001 |  | 1.16 (1.06, 1.26) | 0.001 |  | 1.17 (1.08, 1.27) | <0.001 |  | 1.21 (1.10, 1.33) | <0.001 |  |
| Alcohol |  |  |  |  |  |  |  |  |  |  |  |  |  |
| Yes | 482 | 1.13 (0.98, 1.29) | 0.088 | 0.804 | 1.16 (1.00, 1.34) | 0.052 | 0.847 | 1.28 (1.11, 1.46) | <0.001 | 0.317 | 1.10 (0.94, 1.29) | 0.241 | 0.572 |
| No | 1492 | 1.13 (1.04, 1.23) | 0.003 |  | 1.14 (1.04, 1.23) | 0.003 |  | 1.15 (1.06, 1.25) | 0.001 |  | 1.20 (1.09, 1.32) | <0.001 |  |
| Physical activity |  |  |  |  |  |  |  |  |  |  |  |  |  |
| Low | 136 | 1.31 (0.94, 1.84) | 0.109 | 0.120 | 1.21 (0.87, 1.67) | 0.252 | 0.838 | 1.06 (0.77, 1.45) | 0.721 | 0.901 | 1.15 (0.84, 1.57) | 0.376 | 0.511 |
| Moderate | 759 | 1.21 (1.08, 1.35) | 0.001 |  | 1.14 (1.02, 1.29) | 0.024 |  | 1.19 (1.06, 1.33) | 0.003 |  | 1.11 (0.98, 1.25) | 0.09 |  |
| High | 1079 | 1.07 (0.97, 1.18) | 0.15 |  | 1.13 (1.03, 1.24) | 0.013 |  | 1.18 (1.07, 1.29) | 0 |  | 1.21 (1.07, 1.36) | 0.002 |  |
| BMI, kg/m^2^ |  |  |  |  |  |  |  |  |  |  |  |  |  |
| <24 | 943 | 1.05 (0.92, 1.21) | 0.433 | 0.215 | 1.14 (0.99, 1.31) | 0.064 | 0.952 | 1.12 (0.98, 1.29) | 0.084 | 0.467 | 1.16 (0.99, 1.35) | 0.07 | 0.833 |
| ≥24 | 1031 | 1.18 (1.09, 1.29) | <0.001 |  | 1.14 (1.05, 1.24) | 0.002 |  | 1.20 (1.11, 1.30) | <0.001 |  | 1.18 (1.08, 1.30) | <0.001 |  |
| HOMA-B |  |  |  |  |  |  |  |  |  |  |  |  |  |
| <150.9 | 986 | 1.12 (1.02, 1.23) | 0.02 | 0.679 | 1.14 (1.04, 1.25) | 0.007 | 0.922 | 1.21 (1.11, 1.33) | <0.001 | 0.224 | 1.11 (1.00, 1.24) | 0.053 | 0.851 |
| ≥150.9 | 986 | 1.13 (1.02, 1.26) | 0.024 |  | 1.10 (0.99, 1.22) | 0.083 |  | 1.09 (0.98, 1.21) | 0.125 |  | 1.19 (1.05, 1.34) | 0.008 |  |

**S5 Table. Continued.**

|  | **N** | **SM C34:0** | ***P*** | ***P-_inter_*** | **SM C36:0** | ***P*** | ***P-_inter_*** | **SM C38:0** | ***P*** | ***P-_inter_*** | **SM C40:0** | ***P*** | ***P-_inter_*** |
| --- | --- | --- | --- | --- | --- | --- | --- | --- | --- | --- | --- | --- | --- |
| Age, y |  |  |  |  |  |  |  |  |  |  |  |  |  |
| 50-59 | 1176 | 1.15 (1.03, 1.29) | 0.012 | 0.414 | 1.15 (1.04, 1.27) | 0.007 | 0.857 | 1.14 (1.04, 1.25) | 0.004 | 0.978 | 1.13 (1.03, 1.23) | 0.008 | 0.501 |
| 60-70 | 798 | 1.15 (1.02, 1.30) | 0.024 |  | 1.15 (1.02, 1.29) | 0.018 |  | 1.22 (1.10, 1.35) | <0.001 |  | 1.16 (1.05, 1.29) | 0.004 |  |
| Sex |  |  |  |  |  |  |  |  |  |  |  |  |  |
| Men | 826 | 1.18 (1.04, 1.35) | 0.011 | 0.399 | 1.13 (1.01, 1.26) | 0.038 | 0.410 | 1.13 (1.02, 1.25) | 0.021 | 0.529 | 1.05 (0.94, 1.16) | 0.396 | 0.157 |
| Women | 1148 | 1.13 (1.02, 1.26) | 0.025 |  | 1.22 (1.09, 1.35) | <0.001 |  | 1.22 (1.12, 1.33) | <0.001 |  | 1.22 (1.11, 1.34) | <0.001 |  |
| Region |  |  |  |  |  |  |  |  |  |  |  |  |  |
| Beijing | 935 | 1.11 (1.02, 1.22) | 0.02 | 0.608 | 1.09 (0.99, 1.20) | 0.082 | **0.009** | 1.16 (1.06, 1.26) | 0.001 | 0.256 | 1.15 (1.06, 1.26) | 0.001 | 0.986 |
| Shanghai | 1039 | 1.16 (1.03, 1.30) | 0.015 |  | 1.30 (1.14, 1.48) | <0.001 |  | 1.19 (1.08, 1.32) | 0.001 |  | 1.11 (1.00, 1.23) | 0.052 |  |
| Residence |  |  |  |  |  |  |  |  |  |  |  |  |  |
| Rural | 1154 | 1.20 (1.08, 1.34) | 0.001 | 0.824 | 1.13 (1.02, 1.24) | 0.015 | 0.147 | 1.15 (1.05, 1.25) | 0.002 | 0.300 | 1.10 (1.01, 1.20) | 0.028 | 0.130 |
| Urban | 820 | 1.11 (0.98, 1.25) | 0.114 |  | 1.21 (1.08, 1.37) | 0.002 |  | 1.23 (1.10, 1.37) | <0.001 |  | 1.21 (1.08, 1.34) | 0.001 |  |
| Smoking |  |  |  |  |  |  |  |  |  |  |  |  |  |
| Yes | 549 | 1.11 (0.95, 1.30) | 0.186 | 0.810 | 1.04 (0.91, 1.20) | 0.539 | 0.132 | 1.04 (0.91, 1.19) | 0.574 | 0.149 | 1.00 (0.87, 1.14) | 0.98 | 0.160 |
| No | 1425 | 1.17 (1.06, 1.29) | 0.001 |  | 1.24 (1.13, 1.36) | <0.001 |  | 1.24 (1.15, 1.34) | <0.001 |  | 1.20 (1.11, 1.30) | <0.001 |  |
| Alcohol |  |  |  |  |  |  |  |  |  |  |  |  |  |
| Yes | 482 | 1.19 (1.00, 1.41) | 0.054 | 0.504 | 1.07 (0.93, 1.24) | 0.357 | 0.150 | 1.07 (0.94, 1.23) | 0.318 | 0.188 | 1.06 (0.92, 1.21) | 0.411 | 0.511 |
| No | 1492 | 1.14 (1.04, 1.26) | 0.005 |  | 1.21 (1.10, 1.32) | <0.001 |  | 1.21 (1.12, 1.31) | <0.001 |  | 1.16 (1.07, 1.25) | <0.001 |  |
| Physical activity |  |  |  |  |  |  |  |  |  |  |  |  |  |
| Low | 136 | 1.12 (0.81, 1.54) | 0.491 | 0.419 | 1.18 (0.84, 1.67) | 0.341 | 0.602 | 1.22 (0.83, 1.79) | 0.311 | 0.364 | 1.24 (0.87, 1.78) | 0.239 | 0.301 |
| Moderate | 759 | 1.08 (0.95, 1.23) | 0.249 |  | 1.21 (1.07, 1.36) | 0.002 |  | 1.20 (1.08, 1.34) | 0.001 |  | 1.17 (1.05, 1.31) | 0.004 |  |
| High | 1079 | 1.21 (1.08, 1.36) | 0.001 |  | 1.14 (1.03, 1.26) | 0.012 |  | 1.15 (1.05, 1.25) | 0.002 |  | 1.11 (1.02, 1.22) | 0.018 |  |
| BMI, kg/m^2^ |  |  |  |  |  |  |  |  |  |  |  |  |  |
| <24 | 943 | 1.13 (0.96, 1.32) | 0.137 | 0.990 | 1.14 (0.98, 1.32) | 0.088 | 0.686 | 1.18 (1.04, 1.34) | 0.01 | 0.795 | 1.11 (0.98, 1.26) | 0.096 | 0.701 |
| ≥24 | 1031 | 1.18 (1.07, 1.30) | 0.001 |  | 1.19 (1.09, 1.29) | <0.001 |  | 1.19 (1.10, 1.29) | <0.001 |  | 1.17 (1.08, 1.27) | <0.001 |  |
| HOMA-B |  |  |  |  |  |  |  |  |  |  |  |  |  |
| <150.9 | 986 | 1.06 (0.95, 1.19) | 0.269 | 0.490 | 1.20 (1.09, 1.32) | <0.001 | 0.841 | 1.14 (1.04, 1.24) | 0.03 | 0.836 | 1.12 (1.02, 1.23) | 0.049 | 0.500 |
| ≥150.9 | 986 | 1.18 (1.05, 1.33) | 0.006 |  | 1.14 (1.01, 1.29) | 0.036 |  | 1.18 (1.07, 1.30) | <0.001 |  | 1.14 (1.03, 1.26) | 0.001 |  |

**S5 Table. Continued.**

|  | **N** | **SM C34:1** | ***P*** | ***P-_inter_*** | **SM C36:1** | ***P*** | ***P-_inter_*** | **SM C42:3** | ***P*** | ***P-_inter_*** | **SM (2OH) C34:1** | ***P*** | ***P-_inter_*** |
| --- | --- | --- | --- | --- | --- | --- | --- | --- | --- | --- | --- | --- | --- |
| Age, y |  |  |  |  |  |  |  |  |  |  |  |  |  |
| 50-59 | 1176 | 1.15 (1.04, 1.28) | 0.007 | 0.549 | 1.11 (1.00, 1.24) | 0.042 | 0.347 | 1.12 (1.02, 1.24) | 0.023 | 0.391 | 1.18 (1.07, 1.30) | 0.001 | 0.855 |
| 60-70 | 798 | 1.22 (1.08, 1.37) | 0.001 |  | 1.24 (1.10, 1.39) | <0.001 |  | 1.17 (1.05, 1.31) | 0.004 |  | 1.20 (1.07, 1.34) | 0.001 |  |
| Sex |  |  |  |  |  |  |  |  |  |  |  |  |  |
| Men | 826 | 1.16 (1.03, 1.31) | 0.015 | 0.801 | 1.20 (1.07, 1.36) | 0.003 | 0.415 | 1.07 (0.95, 1.19) | 0.256 | 0.489 | 1.18 (1.05, 1.33) | 0.006 | 0.603 |
| Women | 1148 | 1.19 (1.08, 1.32) | 0.001 |  | 1.15 (1.04, 1.27) | 0.007 |  | 1.21 (1.10, 1.33) | <0.001 |  | 1.24 (1.13, 1.37) | <0.001 |  |
| Region |  |  |  |  |  |  |  |  |  |  |  |  |  |
| Beijing | 935 | 1.14 (1.05, 1.25) | 0.002 | 0.420 | 1.13 (1.03, 1.24) | 0.012 | 0.219 | 1.17 (1.08, 1.28) | <0.001 | 0.775 | 1.11 (1.01, 1.22) | 0.024 | **0.009** |
| Shanghai | 1039 | 1.17 (1.05, 1.30) | 0.005 |  | 1.21 (1.07, 1.37) | 0.003 |  | 1.07 (0.95, 1.21) | 0.287 |  | 1.33 (1.18, 1.50) | <0.001 |  |
| Residence |  |  |  |  |  |  |  |  |  |  |  |  |  |
| Rural | 1154 | 1.19 (1.07, 1.31) | 0.001 | 0.398 | 1.16 (1.05, 1.29) | 0.005 | 0.214 | 1.11 (1.00, 1.22) | 0.04 | 0.215 | 1.20 (1.09, 1.32) | <0.001 | 0.364 |
| Urban | 820 | 1.18 (1.05, 1.33) | 0.006 |  | 1.19 (1.06, 1.34) | 0.003 |  | 1.17 (1.06, 1.30) | 0.002 |  | 1.21 (1.07, 1.36) | 0.002 |  |
| Smoking |  |  |  |  |  |  |  |  |  |  |  |  |  |
| Yes | 549 | 1.08 (0.93, 1.25) | 0.319 | 0.712 | 1.14 (0.98, 1.32) | 0.093 | 0.825 | 1.02 (0.87, 1.19) | 0.799 | 0.775 | 1.08 (0.94, 1.25) | 0.284 | 0.209 |
| No | 1425 | 1.22 (1.12, 1.34) | <0.001 |  | 1.18 (1.08, 1.29) | <0.001 |  | 1.19 (1.09, 1.29) | <0.001 |  | 1.27 (1.16, 1.38) | <0.001 |  |
| Alcohol |  |  |  |  |  |  |  |  |  |  |  |  |  |
| Yes | 482 | 1.14 (0.98, 1.34) | 0.096 | 0.981 | 1.19 (1.01, 1.40) | 0.040 | 0.738 | 1.06 (0.91, 1.24) | 0.437 | 0.511 | 1.13 (0.98, 1.30) | 0.094 | 0.305 |
| No | 1492 | 1.19 (1.09, 1.30) | <0.001 |  | 1.16 (1.06, 1.27) | 0.001 |  | 1.17 (1.07, 1.27) | <0.001 |  | 1.24 (1.13, 1.35) | <0.001 |  |
| Physical activity |  |  |  |  |  |  |  |  |  |  |  |  |  |
| Low | 136 | 1.17 (0.83, 1.63) | 0.366 | 0.541 | 1.47 (1.09, 1.99) | 0.012 | 0.112 | 1.36 (0.86, 2.13) | 0.19 | 0.189 | 1.36 (0.96, 1.93) | 0.084 | 0.602 |
| Moderate | 759 | 1.15 (1.02, 1.30) | 0.021 |  | 1.15 (1.02, 1.30) | 0.021 |  | 1.15 (1.03, 1.29) | 0.013 |  | 1.24 (1.10, 1.39) | <0.001 |  |
| High | 1079 | 1.19 (1.07, 1.33) | 0.001 |  | 1.16 (1.04, 1.29) | 0.007 |  | 1.11 (1.01, 1.23) | 0.035 |  | 1.18 (1.06, 1.30) | 0.002 |  |
| BMI, kg/m^2^ |  |  |  |  |  |  |  |  |  |  |  |  |  |
| <24 | 943 | 1.19 (1.03, 1.37) | 0.021 | 0.597 | 1.12 (0.97, 1.30) | 0.131 | 0.565 | 1.19 (1.03, 1.36) | 0.017 | 0.234 | 1.19 (1.03, 1.37) | 0.017 | 0.975 |
| ≥24 | 1031 | 1.20 (1.09, 1.31) | <0.001 |  | 1.21 (1.10, 1.32) | <0.001 |  | 1.13 (1.04, 1.23) | 0.004 |  | 1.22 (1.12, 1.33) | <0.001 |  |
| HOMA-B |  |  |  |  |  |  |  |  |  |  |  |  |  |
| <150.9 | 986 | 1.08 (0.98, 1.21) | 0.131 | 0.369 | 1.14 (1.03, 1.27) | 0.114 | 0.983 | 1.13 (1.03, 1.25) | 0.016 | 0.059 | 1.17 (1.06, 1.29) | 0.002 | 0.301 |
| ≥150.9 | 986 | 1.21 (1.09, 1.35) | 0.001 |  | 1.14 (1.01, 1.28) | <0.001 |  | 1.12 (1.00, 1.26) | 0.007 |  | 1.24 (1.10, 1.39) | <0.001 |  |

**S5 Table. Continued.**

|  | **N** | **SM (OH) C38:3** | ***P*** | ***P-_inter_*** | **HexCer C20:1** | ***P*** | ***P-_inter_*** |
| --- | --- | --- | --- | --- | --- | --- | --- |
| Age, y |  |  |  |  |  |  |  |
| 50-59 | 1176 | 1.22 (1.09, 1.37) | 0.001 | 0.063 | 1.15 (1.04, 1.28) | 0.006 | 0.813 |
| 60-70 | 798 | 1.14 (1.00, 1.29) | 0.051 |  | 1.17 (1.06, 1.30) | 0.003 |  |
| Sex |  |  |  |  |  |  |  |
| Men | 826 | 1.13 (1.00, 1.28) | 0.060 | 0.794 | 1.14 (1.02, 1.27) | 0.017 | 0.739 |
| Women | 1148 | 1.25 (1.11, 1.41) | <0.001 |  | 1.17 (1.06, 1.30) | 0.001 |  |
| Region |  |  |  |  |  |  |  |
| Beijing | 935 | 1.13 (1.03, 1.24) | 0.010 | 0.400 | 1.15 (1.05, 1.26) | 0.003 | 0.648 |
| Shanghai | 1039 | 1.19 (1.07, 1.34) | 0.002 |  | 1.17 (1.04, 1.31) | 0.010 |  |
| Residence |  |  |  |  |  |  |  |
| Rural | 1154 | 1.16 (1.05, 1.30) | 0.006 | 0.336 | 1.15 (1.05, 1.27) | 0.004 | 0.440 |
| Urban | 820 | 1.24 (1.08, 1.42) | 0.002 |  | 1.18 (1.06, 1.32) | 0.003 |  |
| Smoking |  |  |  |  |  |  |  |
| Yes | 549 | 1.16 (0.98, 1.36) | 0.081 | 0.574 | 1.14 (0.99, 1.31) | 0.070 | 0.952 |
| No | 1425 | 1.21 (1.10, 1.34) | <0.001 |  | 1.17 (1.08, 1.28) | <0.001 |  |
| Alcohol |  |  |  |  |  |  |  |
| Yes | 482 | 1.11 (0.95, 1.30) | 0.181 | 0.663 | 1.21 (1.05, 1.40) | 0.007 | 0.654 |
| No | 1492 | 1.21 (1.10, 1.34) | <0.001 |  | 1.15 (1.06, 1.26) | 0.001 |  |
| Physical activity |  |  |  |  |  |  |  |
| Low | 136 | 1.32 (0.90, 1.92) | 0.155 | 0.806 | 1.13 (0.87, 1.47) | 0.352 | 0.867 |
| Moderate | 759 | 1.25 (1.10, 1.43) | 0.001 |  | 1.19 (1.06, 1.33) | 0.002 |  |
| High | 1079 | 1.16 (1.03, 1.30) | 0.013 |  | 1.16 (1.04, 1.28) | 0.006 |  |
| BMI, kg/m^2^ |  |  |  |  |  |  |  |
| <24 | 943 | 1.19 (1.03, 1.39) | 0.022 | 0.982 | 1.09 (0.95, 1.26) | 0.207 | 0.703 |
| ≥24 | 1031 | 1.21 (1.10, 1.34) | <0.001 |  | 1.19 (1.09, 1.30) | <0.001 |  |
| HOMA-B |  |  |  |  |  |  |  |
| <150.9 | 986 | 1.15 (1.04, 1.28) | 0.032 | 0.101 | 1.13 (1.02, 1.24) | 0.175 | 0.497 |
| ≥150.9 | 986 | 1.17 (1.03, 1.34) | 0.002 |  | 1.14 (1.02, 1.28) | <0.001 |  |

Model was adjusted for age, sex, region (Beijing or Shanghai), residence (urban or rural), educational attainment (0-6 years, 7-9 years, or ≥10 years), current smoking (yes or no), current alcohol drinking (yes or no), physical activity (low, moderate, or high), family history of diabetes (yes or no), and BMI.

Cer, ceramide; HOMA-B, homeostatic model assessment of β-cell function; HexCer, hexosylceramide; SM, sphingomyelin; SM (OH), hydroxyl-sphingomyelin with 1 additional hydroxyl; SM (2OH), hydroxyl-sphingomyelin with 2 additional hydroxyls; T2D, type 2 diabetes.
