# Supplementary material for: Associations among circulating sphingolipids, β-cell function, and risk of developing type 2 diabetes: A population-based cohort study in China
Source: PLoS Med. 2020 Dec 9;17(12):e1003451. doi: 10.1371/journal.pmed.1003451 (PMC7725305; doi:10.1371/journal.pmed.1003451)
Supplement: S6 Table — (DOCX) [file pmed.1003451.s016.docx]

**S6 Table.** **Associations of sphingolipids with insulin resistance, β-cell function, hsCRP, and adiponectin.**

|  | **Fasting insulin** | | **HOMA-IR** | | **HOMA-B** | | **hsCRP** | | **Adiponectin** | |
| --- | --- | --- | --- | --- | --- | --- | --- | --- | --- | --- |
|  | **β (95%CI)** | ***P* value** | **β (95%CI)** | ***P* value** | **β (95%CI)** | ***P* value** | **β (95%CI)** | ***P* value** | **β (95%CI)** | ***P* value** |
| Cer(d18:1/18:1) | 0.013 (-0.008, 0.035) | 2.20×10^-1^ | 0.019 (-0.002, 0.040) | 8.20×10^-2^ | -0.035 (-0.051, -0.019) | **1.86×10^-5^** | 0.206 (0.158, 0.254) | **9.80×10^-17^** | -0.096 (-0.127, -0.066) | **7.70×10^-10^** |
| Cer(d18:1/20:0) | -0.006 (-0.027, 0.015) | 5.87×10^-1^ | 0.000 (-0.021, 0.021) | 9.71×10^-1^ | -0.047 (-0.063, -0.031) | **8.80×10^-9^** | 0.183 (0.135, 0.232) | **1.70×10^-13^** | -0.076 (-0.106, -0.045) | **1.36×10^-6^** |
| Cer(d18:1/20:1) | -0.004 (-0.025, 0.018) | 7.34×10^-1^ | 0.004 (-0.017, 0.025) | 7.20×10^-1^ | -0.063 (-0.079, -0.047) | **1.50×10^-14^** | 0.155 (0.106, 0.203) | **5.10×10^-10^** | -0.082 (-0.112, -0.051) | **1.87×10^-7^** |
| Cer(d18:1/22:1) | 0.013 (-0.012, 0.037) | 3.05×10^-1^ | 0.023 (-0.001, 0.047) | 5.59×10^-2^ | -0.078 (-0.096, -0.060) | **6.00×10^-17^** | 0.142 (0.087, 0.197) | **5.48×10^-7^** | -0.093 (-0.127, -0.058) | **1.93×10^-7^** |
| SM C34:0 | -0.013 (-0.037, 0.011) | 2.92×10^-1^ | 0.000 (-0.024, 0.024) | 9.92×10^-1^ | -0.109 (-0.127, -0.092) | **1.50×10^-32^** | 0.063 (0.008, 0.119) | 2.57×10^-2^ | 0.000 (-0.035, 0.035) | 9.95×10^-1^ |
| SM C36:0 | 0.046 (0.024, 0.068) | **4.53×10^-5^** | 0.048 (0.026, 0.069) | **1.50×10^-5^** | 0.007 (-0.010, 0.023) | 4.44×10^-1^ | 0.124 (0.074, 0.175) | **1.38×10^-6^** | -0.061 (-0.093, -0.030) | **1.00×10^-4^** |
| SM C38:0 | 0.034 (0.011, 0.056) | 3.80×10^-3^ | 0.044 (0.022, 0.067) | **1.00×10^-4^** | -0.066 (-0.083, -0.049) | **4.50×10^-14^** | 0.060 (0.008, 0.113) | 2.41×10^-2^ | -0.099 (-0.131, -0.066) | **3.80×10^-9^** |
| SM C40:0 | 0.053 (0.030, 0.075) | **5.40×10^-6^** | 0.060 (0.038, 0.082) | **1.11×10^-7^** | -0.036 (-0.053, -0.019) | **4.19×10^-5^** | 0.071 (0.019, 0.123) | 7.40×10^-3^ | -0.096 (-0.128, -0.063) | **8.90×10^-9^** |
| SM C34:1 | 0.011 (-0.013, 0.036) | 3.66×10^-1^ | 0.026 (0.001, 0.050) | 3.82×10^-2^ | -0.106 (-0.124, -0.088) | **6.30×10^-30^** | 0.064 (0.007, 0.120) | 2.67×10^-2^ | -0.042 (-0.077, -0.006) | 2.16×10^-2^ |
| SM C36:1 | -0.009 (-0.031, 0.013) | 4.30×10^-1^ | 0.000 (-0.022, 0.022) | 9.97×10^-1^ | -0.074 (-0.091, -0.058) | **1.90×10^-18^** | 0.149 (0.098, 0.199) | **9.60×10^-9^** | -0.064 (-0.096, -0.032) | **1.00×10^-4^** |
| SM C42:3 | 0.040 (0.016, 0.064) | 1.00×10^-3^ | 0.047 (0.023, 0.071) | **1.00×10^-4^** | -0.035 (-0.053, -0.017) | **2.00×10^-4^** | 0.072 (0.017, 0.128) | 1.04×10^-2^ | -0.069 (-0.104, -0.035) | **1.00×10^-4^** |
| SM (2OH) C34:1 | 0.017 (-0.005, 0.039) | 1.28×10^-1^ | 0.023 (0.001, 0.045) | 3.79×10^-2^ | -0.037 (-0.054, -0.020) | **1.47×10^-5^** | 0.121 (0.070, 0.171) | **3.15×10^-6^** | -0.030 (-0.062, 0.002) | 6.56×10^-2^ |
| SM (OH) C38:3 | 0.036 (0.011, 0.062) | 4.90×10^-3^ | 0.048 (0.023, 0.073) | **2.00×10^-4^** | -0.073 (-0.091, -0.054) | **1.00×10^-13^** | 0.044 (-0.014, 0.102) | 1.40×10^-1^ | -0.072 (-0.109, -0.036) | **1.00×10^-4^** |
| HexCer(d18:1/20:1) | -0.008 (-0.030, 0.014) | 4.65×10^-1^ | 0.002 (-0.019, 0.024) | 8.20×10^-1^ | -0.087 (-0.103, -0.071) | **4.40×10^-26^** | 0.038 (-0.012, 0.087) | 1.34×10^-1^ | -0.026 (-0.057, 0.005) | 1.01×10^-1^ |

Model was adjusted for age, sex, region (Beijing or Shanghai), residence (urban or rural), educational attainment (0-6 years, 7-9 years, or ≥10 years), current smoking (yes or no), current alcohol drinking (yes or no), physical activity (low, moderate, or high), family history of diabetes (yes or no), and BMI. Significance are labelled bold.

Cer, ceramide; HOMA-IR, homeostatic model assessment of insulin resistance; HOMA-B, homeostatic model assessment of β-cell function; hsCRP, high-sensitivity C-reactive protein; HexCer, hexosylceramide; SM, sphingomyelin; SM (OH), hydroxyl-sphingomyelin with 1 additional hydroxyl; SM (2OH), hydroxyl-sphingomyelin with 2 additional hydroxyls.
