# Supplementary material for: Associations among circulating sphingolipids, β-cell function, and risk of developing type 2 diabetes: A population-based cohort study in China
Source: PLoS Med. 2020 Dec 9;17(12):e1003451. doi: 10.1371/journal.pmed.1003451 (PMC7725305; doi:10.1371/journal.pmed.1003451)
Supplement: S7 Table — (DOCX) [file pmed.1003451.s017.docx]

**S7 Table.** **Multiple mediation model of fasting glucose, hsCRP, and adiponectin in the associations between sphingolipids and incident T2D.**

| **Sphingolipid or module** | **SP on mediator**  **(Path a: X-M)** | ***P* value** | **Direct effect**  **(Path c': X-Y, adj M)** | ***P* value** | **Indirect effect**  **(Path a*b: X-M-Y)** | ***P* value** | **Proportion**  **mediated^a^ (%)** |
| --- | --- | --- | --- | --- | --- | --- | --- |
| **Fasting glucose** |  |  |  |  |  |  |  |
| Cer(d18:1/18:1) | 0.123 (0.100, 0.147) | <0.001 | 0.037 (-0.077, 0.152) | 0.52 | 0.148 (0.111, 0.193) | <0.001 | 79.1 |
| Cer(d18:1/20:0) | 0.119 (0.096, 0.143) | <0.001 | 0.039 (-0.072, 0.150) | 0.49 | 0.143 (0.110, 0.189) | <0.001 | 80.4 |
| Cer(d18:1/20:1) | 0.167 (0.144, 0.190) | <0.001 | 0.029 (-0.087, 0.144) | 0.63 | 0.200 (0.155, 0.247) | <0.001 | 85.4 |
| Cer(d18:1/22:1) | 0.239 (0.214, 0.264) | <0.001 | -0.095 (-0.228, 0.038) | 0.16 | 0.307 (0.244, 0.376) | <0.001 | 139.8 |
| SM C34:0 | 0.280 (0.256, 0.305) | <0.001 | -0.199 (-0.338, -0.060) | 0.005 | 0.391 (0.315, 0.483) | <0.001 | 179.9 |
| SM C36:0 | 0.068 (0.043, 0.093) | <0.001 | 0.144 (0.029, 0.260) | 0.015 | 0.081 (0.047, 0.116) | <0.001 | 34.9 |
| SM C38:0 | 0.252 (0.229, 0.275) | <0.001 | -0.081 (-0.209, 0.047) | 0.21 | 0.325 (0.251, 0.391) | <0.001 | 128.5 |
| SM C40:0 | 0.206 (0.182, 0.230) | <0.001 | -0.082 (-0.208, 0.044) | 0.20 | 0.264 (0.205, 0.336) | <0.001 | 141.3 |
| SM C34:1 | 0.322 (0.298, 0.346) | <0.001 | -0.217 (-0.363, -0.070) | 0.004 | 0.460 (0.374, 0.551) | <0.001 | 168.2 |
| SM C36:1 | 0.190 (0.166, 0.213) | <0.001 | -0.017 (-0.138, 0.105) | 0.79 | 0.233 (0.181, 0.286) | <0.001 | 99.2 |
| SM C42:3 | 0.177 (0.151, 0.203) | <0.001 | -0.033 (-0.160, 0.095) | 0.62 | 0.219 (0.169, 0.277) | <0.001 | 118.5 |
| SM (2OH) C34:1 | 0.135 (0.111, 0.159) | <0.001 | 0.104 (-0.014, 0.223) | 0.085 | 0.158 (0.117, 0.207) | <0.001 | 58.7 |
| SM (OH) C38:3 | 0.274 (0.248, 0.300) | <0.001 | -0.123 (-0.265, 0.020) | 0.093 | 0.360 (0.280, 0.437) | <0.001 | 136.2 |
| HexCer(d18:1/20:1) | 0.225 (0.202, 0.247) | <0.001 | -0.078 (-0.198, 0.042) | 0.20 | 0.289 (0.229, 0.355) | <0.001 | 128.4 |
| Module yellow | 0.243 (0.219, 0.267) | <0.001 | -0.118 (-0.254, 0.017) | 0.09 | 0.320 (0.245, 0.395) | <0.001 | 152.4 |
| Module turquoise | 0.234 (0.211, 0.258) | <0.001 | -0.082 (-0.209, 0.044) | 0.20 | 0.302 (0.240, 0.367) | <0.001 | 136.0 |
| Module green | 0.217 (0.189, 0.245) | <0.001 | -0.091 (-0.233, 0.051) | 0.21 | 0.276 (0.213, 0.343) | <0.001 | 148.4 |
| Module brown | 0.201 (0.177, 0.224) | <0.001 | -0.100 (-0.222, 0.022) | 0.11 | 0.260 (0.205, 0.321) | <0.001 | 162.5 |
| **hsCRP** |  |  |  |  |  |  |  |
| Cer(d18:1/18:1) | 0.187 (0.146, 0.228) | <0.001 | 0.185 (0.076, 0.294) | <0.001 | 0.001 (-0.020, 0.023) | 0.95 | 0.00 |
| Cer(d18:1/20:0) | 0.162 (0.121, 0.204) | <0.001 | 0.184 (0.076, 0.291) | <0.001 | 0.001 (-0.019, 0.018) | 0.90 | 0.54 |
| Cer(d18:1/20:1) | 0.141 (0.099, 0.183) | <0.001 | 0.232 (0.124, 0.339) | <0.001 | 0.001 (-0.140, 0.019) | 0.93 | 0.00 |
| Cer(d18:1/22:1) | 0.218 (0.096, 0.339) | 0.0004 | 0.218 (0.096, 0.339) | 0.0004 | 0.002 (-0.011, 0.016) | 0.75 | 0.91 |
| SM C34:0 | 0.058 (0.010, 0.106) | 0.02 | 0.200 (0.079, 0.321) | 0.001 | 0.002 (-0.003, 0.011) | 0.63 | 0.99 |
| SM C36:0 | 0.109 (0.066, 0.152) | <0.001 | 0.213 (0.102, 0.325) | <0.001 | 0.002 (-0.011, 0.015) | 0.77 | 0.93 |
| SM C38:0 | 0.057 (0.012, 0.102) | 0.01 | 0.244 (0.135, 0.352) | <0.001 | 0.002 (-0.005, 0.011) | 0.66 | 0.81 |

**S7 Table. Continued.**

|  | **SP on mediator**  **(Path a: X-M)** | ***P* value** | **Direct effect**  **(Path c': X-Y, adj M)** | ***P* value** | **Indirect effect**  **(Path a*b: X-M-Y)** | ***P* value** | **Proportion**  **mediated^a^ (%)** |
| --- | --- | --- | --- | --- | --- | --- | --- |
| SM C40:0 | 0.067 (0.022, 0.111) | 0.004 | 0.192 (0.084, 0.300) | <0.001 | 0.002 (-0.005, 0.011) | 0.63 | 1.03 |
| SM C34:1 | 0.244 (0.125, 0.363) | <0.001 | 0.244 (0.125, 0.363) | <0.001 | 0.002 (-0.006, 0.010) | 0.66 | 0.81 |
| SM C36:1 | 0.135 (0.091, 0.178) | <0.001 | 0.221 (0.107, 0.334) | <0.002 | 0.001 (-0.014, 0.017) | 0.86 | 0.45 |
| SM C42:3 | 0.061 (0.014, 0.109) | 0.01 | 0.195 (0.078, 0.313) | 0.001 | 0.002 (-0.004, 0.012) | 0.62 | 1.02 |
| SM(2OH) C34:1 | 0.105 (0.061, 0.148) | <0.001 | 0.266 (0.151, 0.380) | <0.001 | 0.001 (-0.010, 0.013) | 0.86 | 0.37 |
| SM (OH) C38:3 | 0.045 (-0.005, 0.095) | 0.08 | 0.247 (0.121, 0.372) | <0.001 | 0.002 (-0.003, 0.010) | 0.61 | 0.80 |
| HexCer(d18:1/20:1) | 0.036 (-0.007, 0.079) | 0.10 | 0.220 (0.113, 0.327) | <0.001 | 0.001 (-0.002, 0.008) | 0.65 | 0.45 |
| Module yellow | 0.070 (0.016, 0.123) | 0.01 | 0.210 (0.098, 0.323) | <0.001 | 0.002 (-0.005, 0.012) | 0.60 | 0.94 |
| Module turquoise | 0.168 (0.117, 0.218) | <0.001 | 0.231 (0.116, 0.346) | <0.001 | 0.001 (-0.015, 0.018) | 0.89 | 0.43 |
| Module green | 0.166 (0.107, 0.225) | <0.001 | 0.196 (0.064, 0.329) | 0.004 | 0.003 (-0.016, 0.021) | 0.70 | 1.51 |
| Module brown | 0.062 (0.011, 0.112) | 0.02 | 0.169 (0.058, 0.280) | 0.003 | 0.002 (-0.004, 0.009) | 0.60 | 1.17 |
| **Adiponectin** |  |  |  |  |  |  |  |
| Cer(d18:1/18:1) | -0.136 (-0.178, -0.094) | <0.001 | 0.171 (0.063, 0.279) | 0.002 | 0.014 (-0.001, 0.031) | 0.09 | 7.57 |
| Cer(d18:1/20:0) | -0.102 (-0.144, -0.060) | <0.001 | 0.173 (0.066, 0.280) | 0.002 | 0.011 (0.001, 0.025) | 0.08 | 5.98 |
| Cer(d18:1/20:1) | -0.114 (-0.155, -0.072) | <0.001 | 0.022 (0.114, 0.323) | <0.001 | 0.011 (-0.002, 0.026) | 0.10 | 33.33 |
| Cer(d18:1/22:1) | -0.012 (-0.164, -0.069) | <0.001 | 0.207 (0.086, 0.328) | 0.0008 | 0.013 (-0.0007, 0.030) | 0.08 | 5.91 |
| SM C34:0 | 0.007 (-0.041, 0.055) | 0.77 | 0.202 (0.081, 0.323) | 0.001 | -0.0009 (-0.010, 0.010) | 0.79 | 0.00 |
| SM C36:0 | -0.084 (-0.128, -0.041) | <0.001 | 0.205 (0.094, 0.316) | <0.001 | 0.009 (0.001, 0.024) | 0.09 | 4.21 |
| SM C38:0 | -0.134 (-0.178, -0.089) | <0.001 | 0.233 (0.123, 0.342) | <0.001 | 0.013 (-0.002, 0.031) | 0.10 | 5.28 |
| SM C40:0 | -0.128 (-0.173, -0.084) | <0.001 | 0.181 (0.072, 0.290) | 0.001 | 0.014 (-0.001, 0.031) | 0.08 | 7.18 |
| SM C34:1 | -0.053 (-0.101, -0.004) | 0.03 | 0.239 (0.121, 0.358) | <0.001 | 0.006 (0.0004, 0.020) | 0.16 | 2.45 |
| SM C36:1 | -0.086 (-0.130, -0.043) | <0.001 | 0.211 (-0.099, 0.324) | <0.001 | 0.009 (0.001, 0.025) | 0.09 | 4.09 |
| SM C42:3 | -0.089 (-0.137, -0.042) | <0.001 | 0.187 (0.070, 0.305) | 0.002 | 0.010 (0.002, 0.025) | 0.08 | 5.08 |
| SM(2OH) C34:1 | -0.038 (-0.082, 0.006) | 0.09 | 0.260 (0.147, 0.375) | <0.001 | 0.004 (-0.0001, 0.020) | 0.22 | 1.52 |
| SM (OH) C38:3 | -0.101 (-0.151, -0.051) | <0.001 | 0.238 (0.112, 0.363) | <0.001 | 0.011 (0.001, 0.027) | 0.08 | 4.42 |
| HexCer(d18:1/20:1) | -0.036 (-0.078, 0.007) | 0.10 | 0.217 (0.110, 0.325) | <0.001 | 0.004 (-0.0002, 0.010) | 0.22 | 1.81 |
| Module yellow | -0.090 (-0.124, -0.057) | <0.001 | 0.199 (0.086, 0.312) | <0.001 | 0.014 (0.000, 0.033) | 0.07 | 6.57 |

**S7 Table. Continued.**

|  | **SP on mediator**  **(Path a: X-M)** | ***P* value** | **Direct effect**  **(Path c': X-Y adj M)** | ***P* value** | **Indirect effect**  **(Path a*b: X-M-Y)** | ***P* value** | **Proportion**  **Mediated^a^ (%)** |
| --- | --- | --- | --- | --- | --- | --- | --- |
| Module turquoise | -0.058 (-0.090, -0.026) | <0.001 | 0.222 (0.108, 0.336) | <0.001 | 0.009 (0.001, 0.023) | 0.08 | 3.90 |
| Module green | -0.074 (-0.112, -0.037) | <0.001 | 0.186 (0.055, 0.318) | 0.006 | 0.012 (0.002, 0.029) | 0.07 | 6.06 |
| Module brown | 0.008 (-0.023, 0.040) | 0.61 | 0.173 (0.062, 0.284) | 0.002 | -0.002 (-0.010, 0.004) | 0.64 | 0.00 |

Model was adjusted for age, sex, region (Beijing or Shanghai), residence (urban or rural), educational attainment (0-6 years, 7-9 years, or ≥10 years), current smoking (yes or no), current alcohol drinking (yes or no), physical activity (low, moderate, or high), family history of diabetes (yes or no), and BMI.

^a^This estimate was a pseudo-proportion because its value was not restricted to between 0 and 100.

Cer, ceramide; hsCRP, high-sensitivity C-reactive protein; HexCer, hexosylceramide; SP, sphingolipid; SM, sphingomyelin; SM (OH), hydroxyl-sphingomyelin with 1 additional hydroxyl; SM (2OH), hydroxyl-sphingomyelin with 2 additional hydroxyls; T2D, type 2 diabetes.
