# Supplementary material for: Associations among circulating sphingolipids, β-cell function, and risk of developing type 2 diabetes: A population-based cohort study in China
Source: PLoS Med. 2020 Dec 9;17(12):e1003451. doi: 10.1371/journal.pmed.1003451 (PMC7725305; doi:10.1371/journal.pmed.1003451)
Supplement: S8 Table — (DOCX) [file pmed.1003451.s018.docx]

**S8 Table.** **Characteristics of genetic instruments for sphingolipids.**

| **SNP** | **Nearest gene** | **Chr** | **Position** | **EA** | **NEA** | **EAF** | **Exposure** | | | | **T2D** | | |
| --- | --- | --- | --- | --- | --- | --- | --- | --- | --- | --- | --- | --- | --- |
|  |  |  |  |  |  |  | **β** | **SE** | ***P*** | ***F*** | **β** | **SE** | ***P*** |
| **Cer(d18:1/20:0)** |  |  |  |  |  |  |  |  |  |  |  |  |  |
| rs76415391 | ***CERS4*** | 19 | 8318959 | T | C | 0.176 | -0.092 | 0.015 | **2.88×10^-9^** | 38 | -0.007 | 0.023 | 0.770 |
| rs79224144 | *GPR75-ASB3* | 2 | 53996555 | C | T | 0.078 | -0.100 | 0.020 | 1.08×10^-6^ | 25 | -0.013 | 0.017 | 0.470 |
| rs34192714 | *ATP9B* | 18 | 76958587 | C | T | 0.208 | 0.064 | 0.014 | 3.02×10^-6^ | 21 | 0.020 | 0.110 | 0.860 |
| rs1896315 | *RP11-411G2.2* | 12 | 115401716 | A | G | 0.248 | -0.058 | 0.012 | 3.64×10^-6^ | 23 | 0.000 | 0.006 | 0.990 |
| rs36114260 | *MAP6* | 11 | 75394429 | T | C | 0.072 | 0.096 | 0.021 | 3.68×10^-6^ | 21 | 0.002 | 0.010 | 0.810 |
| rs10054303 | *LOC101929710* | 5 | 95509602 | T | C | 0.259 | 0.058 | 0.012 | 4.30×10^-6^ | 23 | -0.001 | 0.007 | 0.930 |
| rs12189372 | *RP11-231G15.1* | 5 | 107907196 | C | G | 0.348 | -0.053 | 0.011 | 4.87×10^-6^ | 23 | -0.001 | 0.007 | 0.940 |
| rs13030314 | *GFPT1* | 2 | 69520795 | A | G | 0.248 | 0.061 | 0.013 | 4.88×10^-6^ | 22 | -0.011 | 0.006 | 0.085 |
| **Cer(d18:1/20:1)** |  |  |  |  |  |  |  |  |  |  |  |  |  |
| rs11881630 | ***CERS4*** | 19 | 8319455 | T | C | 0.177 | -0.085 | 0.015 | **3.81×10^-8^** | 32 | -0.007 | 0.023 | 0.760 |
| rs6496103 | *LINC01197* | 15 | 95891921 | C | G | 0.364 | -0.055 | 0.011 | 6.92×10^-7^ | 25 | -0.015 | 0.008 | 0.045 |
| rs4838314 | *RP11-343J18.2* | 9 | 128796578 | G | T | 0.186 | -0.069 | 0.014 | 7.99×10^-7^ | 24 | -0.007 | 0.007 | 0.290 |
| rs11017173 | *DOCK1* | 10 | 129095694 | T | C | 0.045 | -0.128 | 0.027 | 2.86×10^-6^ | 22 | -0.025 | 0.011 | 0.024 |
| rs72897064 | *LRRC4C* | 11 | 40490450 | C | A | 0.138 | 0.073 | 0.016 | 4.00×10^-6^ | 21 | -0.002 | 0.009 | 0.860 |
| **SM C34:0** |  |  |  |  |  |  |  |  |  |  |  |  |  |
| rs9889726 | ***LASP1*** | 17 | 37047658 | A | G | 0.287 | 0.038 | 0.007 | **1.18****×10^-8^** | 29 | -0.006 | 0.007 | 0.340 |
| rs1127267 | *ARNT2* | 15 | 80889953 | C | T | 0.122 | 0.044 | 0.009 | 1.76×10^-6^ | 24 | -0.005 | 0.012 | 0.690 |
| rs61746884 | *NFATC1* | 18 | 77306934 | T | C | 0.018 | -0.101 | 0.022 | 3.56×10^-6^ | 21 | 0.016 | 0.019 | 0.400 |
| rs11666165 | *IL12RB1* | 19 | 18167463 | A | G | 0.103 | -0.045 | 0.010 | 4.28×10^-6^ | 20 | -0.004 | 0.008 | 0.610 |
| **SM C36:0** |  |  |  |  |  |  |  |  |  |  |  |  |  |
| rs79371681 | ***LRRC4C*** | 11 | 41434470 | A | C | 0.111 | -0.073 | 0.013 | **4.63×10^-8^** | 32 | -0.005 | 0.012 | 0.660 |
| rs11881630 | *CERS4* | 19 | 8319455 | T | C | 0.177 | -0.061 | 0.012 | 5.21×10^-7^ | 26 | -0.007 | 0.023 | 0.760 |
| rs76500530 | *CACHD1* | 1 | 64934264 | T | C | 0.024 | -0.133 | 0.028 | 1.81×10^-6^ | 23 | 0.009 | 0.022 | 0.700 |
| rs80032050 | *LOC101929147* | 1 | 119765192 | T | G | 0.048 | 0.097 | 0.020 | 1.81×10^-6^ | 24 | -0.013 | 0.009 | 0.150 |
| rs9889726 | *LASP1* | 17 | 37047658 | A | G | 0.287 | 0.042 | 0.009 | 4.31×10^-6^ | 22 | -0.006 | 0.007 | 0.340 |
| rs7174883 | *ARNT2* | 15 | 80904460 | A | G | 0.106 | 0.064 | 0.014 | 4.36×10^-6^ | 21 | -0.130 | 0.069 | 0.061 |

**S8 Table. Continued.**

| **SNP** | **Nearest gene** | **Chr** | **Position** | **EA** | **NEA** | **EAF** | **Exposure** | | | | **T2D** | | |
| --- | --- | --- | --- | --- | --- | --- | --- | --- | --- | --- | --- | --- | --- |
|  |  |  |  |  |  |  | **β** | **SE** | ***P*** | ***F*** | **β** | **SE** | ***P*** |
| **SM C34:1** |  |  |  |  |  |  |  |  |  |  |  |  |  |
| rs72790056 | ***PCDHGA12*** | 5 | 140838943 | A | G | 0.033 | -0.136 | 0.024 | **1.39×10^-8^** | 32 | -0.015 | 0.019 | 0.430 |
| rs79224144 | *GPR75-ASB3* | 2 | 53996555 | C | T | 0.078 | -0.079 | 0.016 | 1.82×10^-6^ | 24 | -0.013 | 0.017 | 0.470 |
| rs588136 | *LIPC* | 15 | 58730498 | C | T | 0.358 | 0.043 | 0.009 | 2.49×10^-6^ | 23 | -0.004 | 0.008 | 0.580 |
| rs2354319 | *LINC00644* | 14 | 62603838 | C | T | 0.362 | 0.041 | 0.009 | 4.78×10^-6^ | 21 | 0.004 | 0.007 | 0.570 |
| rs73617300 | *CFAP61,C20orf26* | 20 | 20312119 | A | G | 0.239 | -0.046 | 0.010 | 4.92×10^-6^ | 21 | -0.220 | 0.270 | 0.410 |
| **SM C42:3** |  |  |  |  |  |  |  |  |  |  |  |  |  |
| rs174535 | ***MYRF*** | 11 | 61551356 | C | T | 0.356 | -0.024 | 0.003 | **3.30×10^-13^** | 64 | -0.028 | 0.007 | <0.001 |
| rs7125097 | *PKNOX2* | 11 | 125192524 | G | A | 0.165 | 0.021 | 0.004 | 1.01×10^-6^ | 28 | -0.012 | 0.020 | 0.530 |
| rs9785210 | *PLGRKT* | 9 | 5372048 | A | C | 0.378 | 0.016 | 0.003 | 1.14×10^-6^ | 28 | -0.002 | 0.007 | 0.780 |
| rs501981 | *NTM* | 11 | 131809462 | G | A | 0.050 | -0.035 | 0.007 | 1.58×10^-6^ | 25 | 0.003 | 0.007 | 0.670 |
| rs11086580 | *RP13-379L11.3* | 20 | 55478973 | C | T | 0.495 | -0.015 | 0.003 | 2.13×10^-6^ | 25 | -0.013 | 0.006 | 0.050 |
| rs111791140 | *AMN1* | 12 | 31858972 | T | C | 0.010 | 0.075 | 0.016 | 2.29×10^-6^ | 22 | 0.002 | 0.013 | 0.890 |
| rs2359181 | *RNU6-443P* | 18 | 41315375 | G | A | 0.084 | 0.027 | 0.006 | 3.63×10^-6^ | 20 | 0.580 | 0.350 | 0.094 |
| rs9822399 | *LMCD1-AS1* | 3 | 8388333 | A | G | 0.192 | 0.020 | 0.004 | 4.35×10^-6^ | 25 | -0.003 | 0.006 | 0.630 |
| **HexCer(18:1/20:1)** | |  |  |  |  |  |  |  |  |  |  |  |  |
| rs13106043 | ***ATP10D*** | 4 | 47561141 | A | G | 0.234 | -0.093 | 0.014 | **1.03×10^-10^** | 44 | 0.001 | 0.006 | 0.830 |
| rs2648408 | *LHFPL4* | 3 | 9579609 | T | C | 0.015 | 0.233 | 0.048 | 1.24×10^-6^ | 24 | -0.003 | 0.013 | 0.820 |
| rs11339221 | *CDC14A* | 1 | 100826655 | T | A | 0.090 | 0.101 | 0.022 | 3.31×10^-6^ | 21 | 0.080 | 0.390 | 0.840 |
| rs151140560 | *CAMKMT* | 2 | 44754503 | T | C | 0.108 | 0.093 | 0.020 | 3.35×10^-6^ | 22 | 0.023 | 0.036 | 0.530 |
| rs16954224 | *RNU6-745P* | 15 | 70481307 | T | C | 0.273 | -0.069 | 0.015 | 3.36×10^-6^ | 21 | -0.005 | 0.008 | 0.490 |
| rs10411408 | *FAM90A28P* | 19 | 53808016 | T | C | 0.036 | 0.161 | 0.035 | 3.76×10^-6^ | 21 | 0.018 | 0.019 | 0.320 |
| rs899437 | *EMP1* | 12 | 13428022 | C | T | 0.460 | 0.057 | 0.012 | 4.22×10^-6^ | 23 | 0.002 | 0.007 | 0.740 |

Data are based on genome-wide significant SNPs using linear regression analysis under an additive genetic model, adjusted for age, sex, region (Beijing or Shanghai), and the first four principal components (PCs) (*n* = 1,976). Data source: diabetes case (*n* = 748)-control (*n* = 4,983) study based on the China Health and Nutrition Survey (CHNS).

Chr, chromosome; Cer, ceramide; EA, effect allele; EAF, effect allele frequency; HexCer, hexosylceramide; NEA, non-effect allele; SE, standard error; SNP, single nucleotide polymorphism; SM, sphingomyelin; SM (OH), hydroxyl-sphingomyelin with 1 additional hydroxyl; SM (2OH), hydroxyl-sphingomyelin with 2 additional hydroxyls; T2D, type 2 diabetes.
