# Supplementary material for: Associations among circulating sphingolipids, β-cell function, and risk of developing type 2 diabetes: A population-based cohort study in China
Source: PLoS Med. 2020 Dec 9;17(12):e1003451. doi: 10.1371/journal.pmed.1003451 (PMC7725305; doi:10.1371/journal.pmed.1003451)
Supplement: S9 Table — (DOCX) [file pmed.1003451.s019.docx]

**S9 Table****. Associations of genetically instrumented sphingolipids with T2D using Mendelian randomization.**

| **Exposure** | **OR (95%CI)** | ***P*** | ***P_FDR*** | **Heterogeneity**  **test *P*** | **MR Egger intercept** | **MR Egger intercept *P*** |
| --- | --- | --- | --- | --- | --- | --- |
| **Cer(d18:1/20:0)** |  |  |  |  |  |  |
| Penalized robust IVW | 1.02 (0.94, 1.10) | 0.652 | 0.79 | 0.99 | -0.007 | 0.608 |
| Penalized weighted median | 1.00 (0.89, 1.13) | 0.968 |  |  |  |  |
| Penalized robust MR-Egger | 1.13 (0.72, 1.79) | 0.601 |  |  |  |  |
| MBE | 1.01 (0.86, 1.18) | 0.924 |  |  |  |  |
| **Cer(d18:1/20:1)** |  |  |  |  |  |  |
| Penalized robust IVW | 1.15 (1.05, 1.26) | **0.002** | **0.007** | 0.28 | -0.003 | 0.795 |
| Penalized weighted median | 1.16 (1.01, 1.33) | **0.032** |  |  |  |  |
| Penalized robust MR-Egger | 1.19 (0.94, 1.51) | 0.140 |  |  |  |  |
| MBE | 1.17 (1.01, 1.37) | **0.039** |  |  |  |  |
| **SM C34:0** |  |  |  |  |  |  |
| Penalized robust IVW | 0.86 (0.79, 0.92) | **<0.001** | **<0.001** | 0.98 | 0.004 | 0.579 |
| Penalized weighted median | 0.87 (0.69, 1.09) | 0.226 |  |  |  |  |
| Penalized robust MR-Egger | 0.86 (0.71, 1.03) | 0.108 |  |  |  |  |
| MBE | 0.85 (0.62, 1.16) | 0.309 |  |  |  |  |
| **SM C36:0** |  |  |  |  |  |  |
| Penalized robust IVW | 0.91 (0.85, 0.98) | **0.013** | **0.03** | 0.41 | -0.002 | 0.545 |
| Penalized weighted median | 0.89 (0.76, 1.05) | 0.164 |  |  |  |  |
| Penalized robust MR-Egger | 0.94 (0.83, 1.05) | 0.273 |  |  |  |  |
| MBE | 0.89 (0.77, 1.04) | 0.134 |  |  |  |  |
| **SM C34:1** |  |  |  |  |  |  |
| Penalized robust IVW | 1.08 (0.95, 1.22) | 0.235 | 0.41 | 0.67 | -0.006 | 0.174 |
| Penalized weighted median | 1.10 (0.91, 1.35) | 0.326 |  |  |  |  |
| Penalized robust MR-Egger | 1.19 (1.08, 1.31) | **<0.001** |  |  |  |  |
| MBE | 1.12 (0.90, 1.39) | 0.317 |  |  |  |  |
| **SM C42:3** |  |  |  |  |  |  |
| Penalized robust IVW | 0.97 (0.78, 1.20) | 0.771 | 0.79 | 0.16 | 0.004 | 0.607 |
| Penalized weighted median | 0.95 (0.72, 1.27) | 0.746 |  |  |  |  |

**S9 Table. Continued.**

| **Exposure** | **OR (95%CI)** | ***P*** | ***P_FDR*** | **Heterogeneity**  **test *P*** | **MR Egger intercept** | **MR Egger intercept *P*** |
| --- | --- | --- | --- | --- | --- | --- |
| Penalized robust MR-Egger | 0.92 (0.67, 1.25) | 0.582 |  |  |  |  |
| MBE | 0.95 (0.76, 1.18) | 0.634 |  |  |  |  |
| **HexCer(d18:1/20:1)** |  |  |  |  |  |  |
| Penalized robust IVW | 0.99 (0.91, 1.08) | 0.790 | 0.79 | 0.84 | 0.004 | 0.244 |
| Penalized weighted median | 1.01 (0.96, 1.07) | 0.610 |  |  |  |  |
| Penalized robust MR-Egger | 0.98 (0.93, 1.04) | 0.508 |  |  |  |  |
| MBE | 1.00 (0.92, 1.09) | 0.948 |  |  |  |  |

Data source and sample size: diabetes case (*n* = 748)-control (*n* = 4,983) study based on the China Health and Nutrition Survey (CHNS).

Cer, ceramide; FDR, false discovery rate; HexCer, hexosylceramide; IVW, inverse variance weighting; MR, Mendelian randomization; MBE, mode-based estimation; OR, odds ratio; SM, sphingomyelin; SM (OH), hydroxyl-sphingomyelin with 1 additional hydroxyl; SM (2OH), hydroxyl-sphingomyelin with 2 additional hydroxyls; T2D, type 2 diabetes; WM, weighted median.
