# Supplementary material for: Associations among circulating sphingolipids, β-cell function, and risk of developing type 2 diabetes: A population-based cohort study in China
Source: PLoS Med. 2020 Dec 9;17(12):e1003451. doi: 10.1371/journal.pmed.1003451 (PMC7725305; doi:10.1371/journal.pmed.1003451)
Supplement: S1 Text — (DOCX) [file pmed.1003451.s020.docx]

Statistical Analysis Plan

Associations between plasma sphingolipids and incident type 2 diabetes (T2D)

Date: August 2018

**Research question:**

- Are plasma sphingolipids associated with incident T2D?
- Could sphingolipids improve the predictivity for future T2D beyond conventional risk factors?

**Exposures:** 76 sphingolipid species

**Outcome:** Incident T2D

**Timescale:** 6-year follow-up

**Exclusions:**

- loss to follow-up;
- having T2D at baseline;
- lack of lipidomics data.

**Covariates:**

Age, sex, region, residence, education attainment, current smoking, current alcohol drinking, physical activity, family history of diabetes, and BMI.

**Main analyses:**

- Baseline characteristics: to compare population characteristics between incident T2D cases and non-cases, as well as differences in sphingolipid distribution.
- Correlations analysis: to calculate the correlations among the sphingolipids, as well as sphingolipids with metabolic traits including glycemic traits, blood lipids, CRP, and adiponectin.
- Associations between sphingolipids and incident T2D:

1. RR per SD: to examinine the effect sizes of incident T2D per SD increment of sphingolipids;
2. Quartile analysis: to compare the effect sizes of T2D risk based on quartiles of sphingolipids;
3. Exploratory analysis: to further controlling systolic blood pressure, blood lipids (mainly LDL-cholesterol), HOMA-IR, HOMA-B, fasting glucose, inflammatory factors (e.g., CRP, IL-6), adipocytokines (e.g., adiponectin), and liver function (GGT) in the mode and to explore the potential dependent and/or mediation effects.

- Stratified analysis: to investigate whether age (< 59 y or ≥ 59 y), sex (men or women), region (Beijing or Shanghai), residence (urban or rural), smoking (yes or no), alcohol drinking (yes or no), physical activity (low, moderate, or high), and BMI (< 24 kg/m^2^ or ≥ 24 kg/m^2^) could modify sphingolipid-T2D associations.
- Sensitivity analysis: define T2D further including HbA1c ≥ 6.5% as a diagnosis criteria.
- WGCNA network analysis: to identify sphingolipid sub-networks associated with incident T2D.
- Elastic net and/or lasso model for T2D prediction: to identify sphingolipids using elastic or lasso regression and assess its predictive ability for future T2D beyond the traditional risk factors.

**Subsequent analyses:**

After the initial main analyses, we also planned to investigate whether the identified sphingolipid-T2D relationships are causal using genetical instruments. In addition, we sought to explore and compare the possible mediation effects by different mediators such as insulin resistance, β-cell function, inflammatory factors, and adipocytokines.
