# Supplementary material for: Associations among circulating sphingolipids, β-cell function, and risk of developing type 2 diabetes: A population-based cohort study in China
Source: PLoS Med. 2020 Dec 9;17(12):e1003451. doi: 10.1371/journal.pmed.1003451 (PMC7725305; doi:10.1371/journal.pmed.1003451)
Supplement: S2 Text — (DOCX) [file pmed.1003451.s021.docx]

**Lipidomic measurement**

**Lipid extraction.** Lipids were extracted following a modified methyl tert-butyl ether (MTBE) protocol [1]. Briefly, a total of 10 μL thawed plasma was mixed with 60 μL methanol and 200 μL MTBE in a 1.5 mL Eppendorf tube. The mixture was shaken at room temperature for 30 min. Next, 50 μL deionized water was added and vortexed for 1 min, and then centrifuged at 3000 g for 10 min. After centrifugation, the upper organic phase in each tube was transferred into a new 1.8 mL glass vial, spiked with 30 µL of multiple internal standards (Sciex, Foster City, CA), and then dried under gentle nitrogen stream. All the lipid extracts were re-dissolved in 200 μL dichloromethane-methanol (1:1, v/v) mixture with 10 mM ammonium acetate prior to analysis.

**Mass spectrometry analysis.** Lipidomics analysis was performed on Nexera X2 LC-30AD system (Shimadzu Scientific Instruments, Marlborough, MA) combined with Sciex 5500 QTRAP Mass Spectrometer (Applied Biosystems/Sciex). ACQUITY UPLC BEH HILIC Column (130Å, 2.1 X 100 mm, 1.7 µm; Waters Corp) was used for chromatographic separation. Analyst 1.6.3 software (AB Sciex, Foster City, CA) was applied for data acquisition. Plasma samples were randomly analyzed and quality control samples were injected every 10 samples to assess the data repeatability. Sample injection volume in both ionization modes was 5 μL. The sample temperature and column temperature were maintained at 4 °C and 40 °C, respectively. The mobile phases for eluting lipids contained 50:50 (v/v) acetonitrile /water with 10 mM ammonium acetate (pH 8.0) (A) and pure acetonitrile (B). The flow rate of mobile phases was 300 μL/min. The gradient elution program was performed as follow: 0.1 min, 85% B; 7.5 min, 65% B; 8.5 min, 5% B; 11 min, 5% B; 11.1 min, 85% B; 15 min, 85% B.

Mass spectrometry analyses were performed by using electrospray ionization in positive and negative modes. Data were acquired in multiple reaction monitoring (MRM) mode. The curtain gas was set as 35 psi and collision gas was set as medium. The pressure of ion source gas 1 was 40 psi and gas 2 was 50 psi. Ion spray voltage was 5.5 kV in the positive mode and was -4.5 kV in the negative mode. Source gas temperature was 500 °C.

**Data quantification.** Quantitative calculation was based on the peak area ratio of confirmed transitions to their corresponding stable isotope-labeled internal standards (IS) by using MultiQuant 3.0.2 Software (Sciex, Foster City, CA). After excluding those lipids with missing rate > 20%, 1,032 lipid species were quantified. Further excluding species with high coefficients of variations (CV) > 30%, we profiled 728 lipid species including phosphatidylcholine (PC), lysophosphatidylcholine (LPC), phosphatidylethanolamine (PE), alkenylphosphatidylethanolamine (PE-P), alkylphosphatidylethanolamine (PE-O), phosphatidylserine (PS), ceramide (Cer), dihydroceramide (dhCer), sphingomyelin (SM), hexosylceramide (HexCer), glucosylceramide (GlcCer), lactosylceramide (LacCer), cholesterol ester (CE), diacylglycerol (DAG), and triglycerol fraction.

In the current study, we used data of 76 sphingolipids (Cers, dhCers, SMs, and GSLs). Sphingolipid subclasses were annotated according to their chemical structures: 1) Cers as d18:1 sphingoid base:SFA or MUFA; 2) dhCers as d18:0 sphingoid base:SFA or MUFA; 3) SMs as total carbon atoms:total double bonds; and 4) GSL as d18:0 or d18:1 sphingoid base:SFA or MUFA. Regarding the SM, it has been repeatedly reported that the most common sphingoid base in human blood is sphingosine with an 18-carbon chain di-hydroxylated at positions 1 and 3 and with a double bond at position 4, with smaller amounts of other structures (d16:1 to d19:1). The fatty acids vary in chain length (14-26 carbon atoms), of which over half are saturated fatty acid such as C16:0 and C18:0 [2]. Therefore, we can carefully infer that SM C34:1 can be probably named as SM(d18:1/16:0) or SM(d16:1/18:0). Similarly, SM C36:1 can be probably named as SM(d18:1/18:0).

**Genome-wide association study (GWAS)**

Associations between single-nucleotide polymorphisms (SNPs) and individual sphingolipids were determined by linear regression analysis under an additive genetic model, after adjustment for age, sex, region (Beijing or Shanghai), and the first four principal components. Genomic control inflation factors (λ) were calculated to account for under-estimation of the standard errors of effect [3]. The explained variance for identified sphingolipids was calculated by 2*MAF*(1– MAF)**β^2^*_,_ where MAF is the minor allele frequency and *β* is coefficient for one copy of the allele [4]. For the following MR analysis, genetic variants with *P* value below 5×10^-6^ were included. To ensure that contribution of selected SNPs was independent, PLINK clumping function was applied to remove correlated SNPs (four main flags: --clump-p1 5×10^-6^ --clump-p2 1×10^-5^ -clump-r2 0.2 --clump-kb 1000).

**Mendelian randomization (MR) analysis**

To explore the potential causal relationships of identified sphingolipids on risk of T2D, a 2-sample MR design was utilized in the current analysis.

**Genetic variants and data sources.** Summary-level data for associations between genetic variants and sphingolipids were derived from our GWAS study (*n* = 1,976) using linear model adjusted for age, sex, region, and the first four principal components. Considering a relatively small sample size, the sphingolipid-associated SNPs with *P* < 5×10^-6^ were also extracted. *F*-statistics were calculated to assess the strength of genetic instruments using instrumental-variable regression. A cutoff of 10 was used as a “rule of thumb” to distinguish strong instruments from weak instruments [5]. Summary statistics for the associations of selected sphingolipid-associated SNPs with T2D were obtained from the China Health and Nutrition Survey (http://mohlke.web.unc.edu/data/), which involved 748 T2D case and 4,983 controls [6]. GWAS for T2D was adjusted for age, age2, BMI, gender, and principal component 1 and T2D cases in this study were assessed if they met one or more of the following components: *1)* HbA_1c_ ≥ 6.5%; *2)* fasting blood glucose ≥ 7.0 mmol/L; *3)* self-reported; or *4)* reported taking anti-diabetic medication. Ethical approval was not obtained because this summary-level data involved public source.

**Data processing and analysis.** The use of genetic instruments associated with the identified sphingolipids were obtained from our study population. All these SNPs were in different genomic regions and in weak linkage equilibrium by using PLINK clumping (*r^2^* < 0.2). When SNPs were not available in outcome dataset, these variants were further removed. In order to ensure the contribution of selected SNPs were solely associated with T2D via sphingolipids, pleiotropy was checked by using PhenoScanner (http://www.phenoscanner.medschl.cam.ac.uk/). Finally, the remaining SNPs were ranked from the smallest to the largest *P*-values prior to next analysis.

**MR method.** After excluding pleiotropic SNPs, we calculated Wald ratio of each instrument with T2D. Genetic associations of sphingolipids with T2D risk were calculated using penalized robust inverse variance weighting (IVW) method [7]. When number of genetic instruments was 3 or lower, fixed-effect model was used, otherwise, random-effect model was applied. Cochran’s *Q*-test and statistic *I^2^* were utilized here to evaluate the genetic heterogeneity of summarized data. *P* < 0.05 and *I^2^* > 50% were considered as the significant heterogeneity. To account for multiple testing, we used a false discovery rate (FDR) correction. To obtain a robust estimate and avoid potential bias, penalized weighted median, mode-based estimation (MBE), and MR-Egger regression asymmetry test were also conducted in our analysis [8, 9]. All reported associations are shown as odds ratios (ORs) per log-transformed standard deviation (SD) and 95% confidential intervals (CIs).

**Leave-one-out validation.** A leave-one-out validation was performed to test the sensitivity of genetic instruments. In brief, a SNP among the selected SNPs was removed to carry out penalized robust IVW point estimate. The fluctuation of the estimate before and after removing the SNP reflect the effect of this one on conclusion. This process was repeated for each SNP.

All statistical tests were undertaken using the R package of MendelianRandomization (version 0.3.0). Two-sided *P* value < 0.05 was considered as statistically significant.

**References**

1. Matyash V, Liebisch G, Kurzchalia TV, Shevchenko A, Schwudke D. Lipid extraction by methyl-tert-butyl ether for high-throughput lipidomics. J Lipid Res. 2008;49(5):1137-46. doi: 10.1194/jlr.D700041-JLR200. PubMed PMID: 18281723; PubMed Central PMCID: PMCPMC2311442.

2. Quehenberger O, Armando AM, Brown AH, Milne SB, Myers DS, Merrill AH, et al. Lipidomics reveals a remarkable diversity of lipids in human plasma. J Lipid Res. 2010;51(11):3299-305. doi: 10.1194/jlr.M009449. PubMed PMID: 20671299; PubMed Central PMCID: PMCPMC2952570.

3. Devlin B, Roeder K. Genomic control for association studies. Biometrics. 1999;55(4):997-1004. doi: 10.1111/j.0006-341x.1999.00997.x. PubMed PMID: 11315092.

4. Hu Y, Li H, Lu L, Manichaikul A, Zhu J, Chen YD, et al. Genome-wide meta-analyses identify novel loci associated with n-3 and n-6 polyunsaturated fatty acid levels in Chinese and European-ancestry populations. Hum Mol Genet. 2016;25(6):1215-24. doi: 10.1093/hmg/ddw002. PubMed PMID: 26744325; PubMed Central PMCID: PMCPMC4764197.

5. Staiger D, Stock JH. Instrumental variables regression with weak instruments. Econometrica. 1997;65(3):557-86. doi: Doi 10.2307/2171753. PubMed PMID: WOS:A1997WV90300003.

6. Spracklen CN, Shi J, Vadlamudi S, Wu Y, Zou M, Raulerson CK, et al. Identification and functional analysis of glycemic trait loci in the China Health and Nutrition Survey. PLoS Genet. 2018;14(4):e1007275. doi: 10.1371/journal.pgen.1007275. PubMed PMID: 29621232; PubMed Central PMCID: PMCPMC5886383.

7. Cheng L, Zhuang H, Yang S, Jiang HJ, Wang S, Zhang J. Exposing the Causal Effect of C-Reactive Protein on the Risk of Type 2 Diabetes Mellitus: A Mendelian Randomization Study. Front Genet. 2018;9. doi: ARTN 657

10.3389/fgene.2018.00657. PubMed PMID: WOS:000453905700001.

8. Bowden J, Smith GD, Haycock PC, Burgess S. Consistent Estimation in Mendelian Randomization with Some Invalid Instruments Using a Weighted Median Estimator. Genet Epidemiol. 2016;40(4):304-14. doi: 10.1002/gepi.21965. PubMed PMID: WOS:000374542600005.

9. Bowden J, Smith GD, Burgess S. Mendelian randomization with invalid instruments: effect estimation and bias detection through Egger regression. International Journal of Epidemiology. 2015;44(2):512-25. doi: 10.1093/ije/dyv080. PubMed PMID: WOS:000357106100015.
